# Protruding Pt single-sites on hexagonal ZnIn_2_S_4_ to accelerate photocatalytic hydrogen evolution

**DOI:** 10.1038/s41467-022-28995-1

**Published:** 2022-03-11

**Authors:** Xiaowei Shi, Chao Dai, Xin Wang, Jiayue Hu, Junying Zhang, Lingxia Zheng, Liang Mao, Huajun Zheng, Mingshan Zhu

**Affiliations:** 1 Department of Applied Chemistry, Zhejiang University of Technology, 310032 Hangzhou, P.R. China; 2 Guangdong Key Laboratory of Environmental Pollution and Health, School of Environment, Jinan University, 511443 Guangzhou, P.R. China; 3 School of Physics, Beihang University, 100191 Beijing, P.R. China; 4 School of Materials Science and Physics, China University of Mining and Technology, 221116 Xuzhou, P.R. China

**Keywords:** Photocatalysis, Nanoscale materials, Surface chemistry

## Abstract

Single-site cocatalysts engineered on supports offer a cost-efficient pathway to utilize precious metals, yet improving the performance further with minimal catalyst loading is still highly desirable. Here we have conducted a photochemical reaction to stabilize ultralow Pt co-catalysts (0.26 wt%) onto the basal plane of hexagonal ZnIn_2_S_4_ nanosheets (Pt_SS_-ZIS) to form a Pt-S_3_ protrusion tetrahedron coordination structure. Compared with the traditional defect-trapped Pt single-site counterparts, the protruding Pt single-sites on *h*-ZIS photocatalyst enhance the H_2_ evolution yield rate by a factor of 2.2, which could reach 17.5 mmol g^−1^ h^−1^ under visible light irradiation. Importantly, through simple drop-casting, a thin Pt_SS_-ZIS film is prepared, and large amount of observable H_2_ bubbles are generated, providing great potential for practical solar-light-driven H_2_ production. The protruding single Pt atoms in Pt_SS_-ZIS could inhibit the recombination of electron-hole pairs and cause a tip effect to optimize the adsorption/desorption behavior of H through effective proton mass transfer, which synergistically promote reaction thermodynamics and kinetics.

## Abstract (web summary)

An alternative approach to defect-trapped Pt single-sites on a semiconductor is reported. Here, protruding Pt sites inhibit charge recombination and cause a tip effect which enhances H_2_ evolution yield rates with minimal co-catalyst loading.

## Introduction

Water splitting for hydrogen (H_2_) generation through solar light has attracted increasing attention since it supplies a significant carbon-neutral technology for zero-emission renewable energy evolution. The design of a photocatalyst with high efficiency and long durability is a focused task for researchers to scale up H_2_ evolution reaction (HER) in the past decades^1^. Two-dimensional (2D) hexagonal ZnIn_2_S_4_ (*h*-ZIS), a typical ternary chalcogenide with favorable H adsorption features at edge S atom in (110) facet ($\Delta {G}_{{{{{{\rm{H}}}}}}}^{* }$ = −0.16 eV) and robust resistance to photocorrosion, has been regarded as a promising candidate for photocatalytic water splitting^2–6^. The current guiding principles for advancing the catalytic performance of *h*-ZIS are as follows. First, increase the active site density in *h*-ZIS through preferentially exposing the edge sites^6,7^. Unfortunately, unleashing the intrinsically high activity of *h*-ZIS is still retarded by the severe recombination of electron-hole pairs, where only a small quantity of electrons could survive at the active sites. Second, create in-plane sulfur vacancies or dope metallic heteroatoms to substitute Zn atoms^7–10^. The lifetime of photoexcited electrons is prolonged, and the basal-plane S atoms in those *h*-ZIS are also stimulated as centers for HER; however, these S sites suffer from less favorable hydrogen adsorption features ($\Delta {G}_{{{{{{\rm{H}}}}}}}^{* }$ = −0.25 eV) despite the increased site density^9^. Apparently, *h*-ZIS only becomes applicable toward photocatalytic HER when the rapid carrier recombination and limited active site obstacles are simultaneously overcome.

As a lamellar material, the basal plane of *h*-ZIS provides plenty of platforms for noble-metal nanoparticles loading, especially platinum (Pt), while the scarcity and high cost of the noble-metal co-catalysts tremendously inhibit their large-scale implementation^2,11–13^. Alternatively, single-site co-catalysts (SSCs) emerge as a frontier for catalysis science due to their high atom efficiency and outstanding activity^14,15^. The strong metal-support interaction caused by metal atoms and coordinated atoms would affect the charge distributions and introduce the electronic structure modifications, which influence the electron-hole pairs recombination and the adsorption behavior during the catalytic process, and eventually change their catalytic activity and selectivity^16–19^. One of the effective strategies for advanced SSCs is to produce more active sites through increasing metal loading with no aggregation, and accordingly, several Pt single-site (Pt_SS_)-based photocatalysts (Pt loading with 8.7 wt%^20^ or 12.0 wt%^21^) have exhibited exciting H_2_ evolution rate and observable bubbles under visible light irradiation. For practical applications, achieving maximum catalytic performance with minimal noble-metal atoms is essentially required. Recently, Pt SSCs supported on highly curved substrates were successfully prepared as electrocatalyst to mimic the metal sites at the edges and corners of particles^22^. Owing to the accumulation of electrons around Pt regions triggered by the tip effect, an accelerated HER kinetics was achieved. Principally, the generation of tip enhancement is biased onto curvature-rich configurations (typically corner, vertex, or protrusion)^23^. To this end, direct anchoring Pt SSCs onto *h*-ZIS nanosheets might be an effective approach to form tridimensional protrusion that could produce high- and cost-efficient photocatalysts for HER.

In this work, a photochemical route was employed to synthesize *h*-ZIS with Pt single sites (Pt_SS_-ZIS)^24^. The light-induced reaction is more moderate and controllable than traditional annealing methods, so the structure of *h*-ZIS could be well preserved without generating any vacancy defects. During photochemical processes, PtCl_6_^2−^ ions were reduced and concurrently immobilized on the basal plane of *h*-ZIS nanosheets, forming Pt–S_3_ tetrahedron coordination structure with surrounding S atoms. Experiments and simulations jointly manifest that the atomically dispersed Pt atoms could serve as sinks to facilitate the separation of photoexcited electron-hole pairs as well as active centers to enhance the HER performance through the accelerating catalytic kinetics. As a result, the synergetic effect of atomic-level Pt and *h*-ZIS produces a higher photocatalytic activity for H_2_ evolution, where the activity outperforms that of defect-trapped Pt single-site counterpart. In addition, a thin film of Pt_SS_-ZIS on the solid substrate could readily be achieved through a drop-casting approach, and a large amount of H_2_ bubbles are generated during light irradiation (Supplementary Movies 1 and 2).

## Results

### Structure analysis and characterization

Ultrathin *h*-ZIS nanosheets with thickness ranging from 2.46 to 4.94 nm were prepared by a hydrothermal method (Supplementary Fig. 1)^9^. H_2_PtCl_6_ aqueous solution was introduced into *h*-ZIS dispersion with magnetic stirring. The interfacial charges of *h*-ZIS and Pt species (HPtCl_6_^−^ or PtCl_6_^2−^) were opposite, so they would be spontaneously assembled through electrostatic interaction in the solution, with Pt-ZIS mixture generated (Supplementary Fig. 2). After irradiation under visible light for 60 min, Pt sites were immobilized on *h*-ZIS, and the mixture was centrifuged and collected (see the Experimental Section and Supplementary Fig. 3). By alerting the volume of added H_2_PtCl_6_, Pt loading content could be tuned, as quantified by the inductively coupled plasma optical emission spectroscopy (ICP-OES) analysis (Supplementary Table 1). Additionally, the molar ratio of Zn and In in Pt_0.3_-ZIS was calculated to be 0.225:0.451, which is consistent with the theoretical molar ratio of 1:2.

**Fig. 1:**
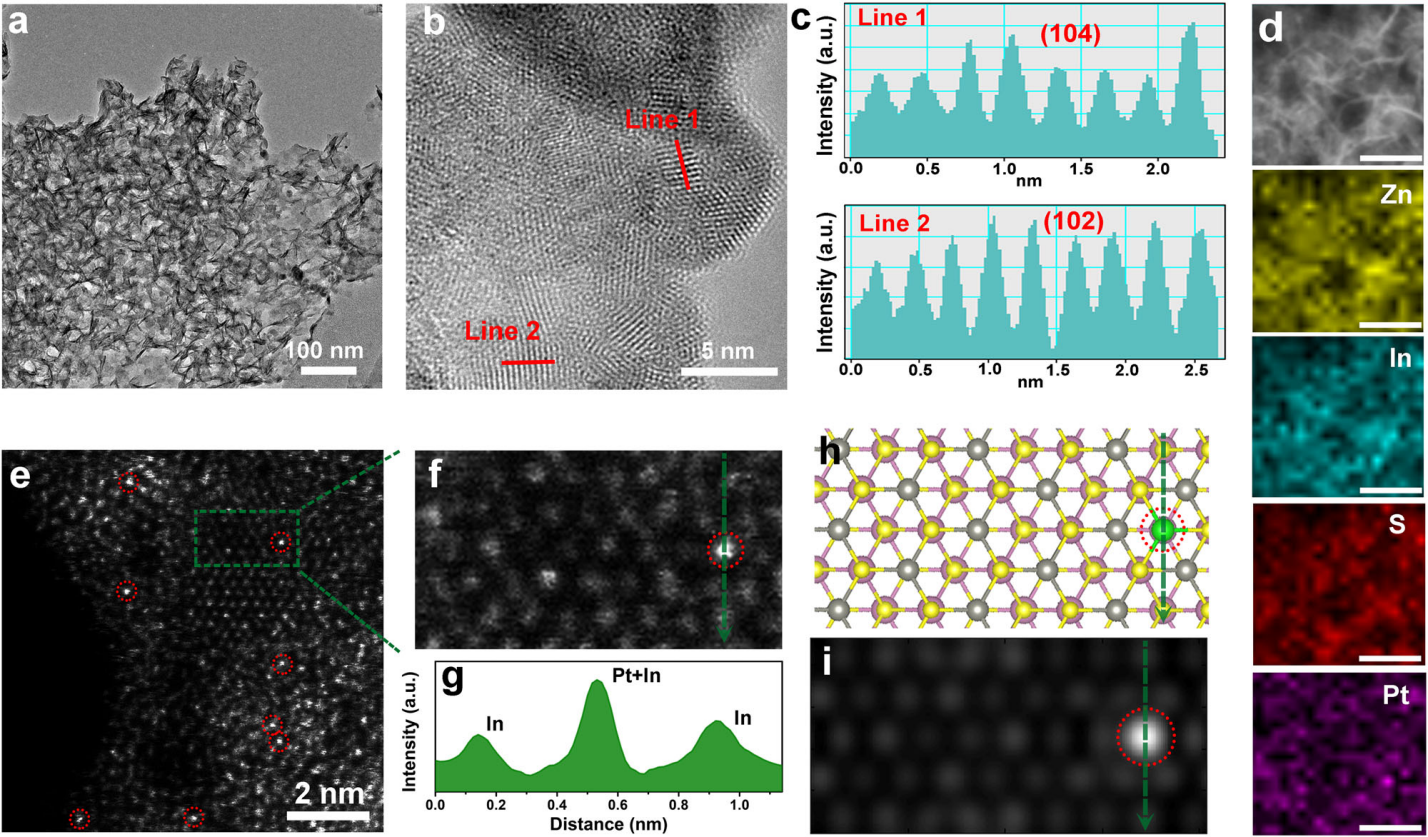
Structural characterization of Pt_0.3_-ZIS. **a** TEM image of Pt_0.3_-ZIS. **b**, **c** HRTEM image of Pt_0.3_-ZIS. **d** Elemental mapping of Pt_0.3_-ZIS. The scale bars are 50 nm. **e** HAADF-STEM image of Pt_0.3_-ZIS. **f** Magnified HAADF-STEM image of Pt_0.3_-ZIS. **g** Strength profiles from the areas labeled by green line. **h** Optimized structure of Pt_0.3_-ZIS. The yellow, gray, pink, and green spheres represent the S, Zn, In, and Pt atom, respectively. **i** Simulated HAADF-STEM image of Pt_SS_-ZIS according to DFT-optimized structure.

X-ray diffraction (XRD) patterns and Raman spectra with negligible changes are observed between *h*-ZIS and Pt-ZIS, suggesting that Pt atoms incorporation does not destroy the crystal structure of *h*-ZIS (Supplementary Fig. 4). Transmission electronmicroscopy (TEM) and high-resolution TEM (HRTEM) images in Supplementary Fig. 5 depict a sheet-like structure of *h*-ZIS and the lattice fringe of 0.41 nm corresponds to the (006) facet. After Pt deposition, the obtained Pt-ZIS nanosheets maintain the thickness (3.10–5.11 nm) of pristine *h*-ZIS recorded by atomic force microscope (AFM) (Supplementary Figs. 6 and 7). As shown in TEM and HRTEM images, no Pt nanoparticles are observed with Pt loading content in the range of 0.1–1.4 wt%, and the energy-dispersive X-ray spectroscopy (EDS) also exhibits homogeneous dispersion of Pt on *h*-ZIS nanosheets without any aggregation (Fig. 1a–d and Supplementary Figs. 8–10). When further increasing the Pt amount to 3.0 wt%, nanoparticles were formed, which is proved by the green circles and corresponding EDS spectrum (Supplementary Fig. 11). In addition, the lattice fringe of 0.293 and 0.196 nm attribute to the *h*-ZIS (104) and Pt (200) facet, respectively. To reveal the configuration of Pt co-catalyst on *h*-ZIS nanosheets, aberration-corrected high angle annular dark field STEM (HAADF-STEM) measurements were carried out on *h*-ZIS and Pt_0.3_-ZIS. Since the contrast in HAADF-STEM image is proportional to the square of atomic number, Pt is much brighter than Zn, In, and S atoms, and the atomically dispersed bright spots (circled) in Fig. 1e and Supplementary Fig. 12 confirm the formation of single Pt atoms^25^. On the contrary, no bright spots could be observed in *h*-ZIS, and the cross-sectional profiles of atom contrast show almost identical intensity (Supplementary Fig. 13). As a result, the cross-sectional intensity in Fig. 1f and g, together with the ICP-OES results and electron spin resonance (ESR) spectra (Supplementary Fig. 14), directly identify that Pt SSCs exist, not as interior dopants substituting for Zn or In in *h*-ZIS skeletons, but as external adatoms conjugating with *h*-ZIS to engender tridimensional protrusions, different from extensively reported planar geometry of metal-N_x_- and defect-trapped-SSCs^13,26^. To illustrate the exact position of Pt single sites, density functional theory (DFT) calculations were carried out to determine the energies of Pt atoms on various sites. Six different locations were established, including Zn-S hollow site, Zn atop, S atop on Zn-S plane, In-S hollow site, In atop, and S atop on In-S plane, respectively, and Zn-S hollow site with the largest adsorption energy is confirmed to be the most stable location for Pt atom occupation (Supplementary Figs. 15–17). The distribution of Pt atoms on *h*-ZIS and their propensity to agglomerate was also examined by calculating the energy difference between an isolated Pt atom and a Pt dimer (Δ*E*_d_), in which the total energy of the isolated configuration was used as reference energy. As shown in Supplementary Fig. 18, it is more favorable for the Pt atoms to be isolated at Zn-S hollow site due to the positive Δ*E*_d_ of 2.03 eV, which fully supports the experimental observations of the Pt single atom in Pt_0.3_-ZIS. Based on the DFT-optimized structure, a STEM simulation on Pt SSCs dispersed *h*-ZIS was performed. The simulated result is in good agreement with the experimental HAADF-STEM image, demonstrating that Pt single sites prefer to chemisorb above the Zn-S hollow site in *h*-ZIS basal plane (Fig. 1h and i).

### Electronic states of atoms in Pt-ZIS

**Fig. 2:**
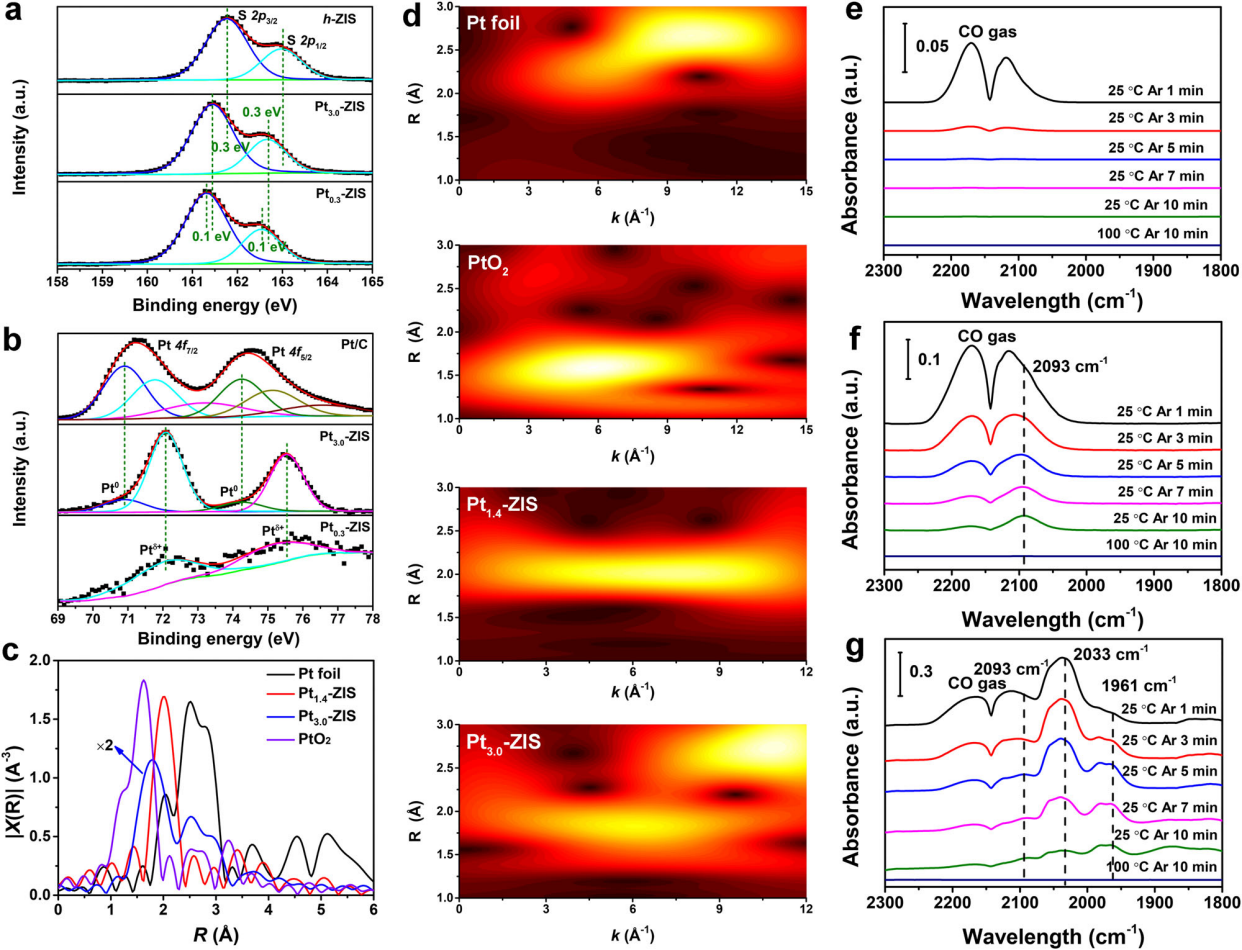
Electronic states of atoms in photocatalysts. **a** High-resolution XPS spectra (S *2p*) of *h*-ZIS, Pt_3.0_-ZIS, and Pt_0.3_-ZIS. **b** High-resolution XPS spectra (Pt *4f*) of Pt/C, Pt_3.0_-ZIS, and Pt_0.3_-ZIS. **c** Fourier transform of *k*^2^-weighted Pt L_3_-edge of the EXAFS spectra for Pt foil, PtO_2_, Pt_1.4_-ZIS, and Pt_3.0_-ZIS. **d** Wavelet transform for the *k*^2^-weighted EXAFS spectra of Pt foil, PtO_2_, Pt_1.4_-ZIS, and Pt_3.0_-ZIS. *R* is the interatomic distance. FTIR spectra of CO adsorbed after the desorption processes for **e** *h*-ZIS, **f** Pt_0.3_-ZIS, and **g** Pt_3.0_-ZIS.

Elemental composition and chemical states of Pt-ZIS were characterized by X-ray photoelectron spectroscopy (XPS). The high-resolution Zn *2p* and In *3d* XPS peaks corresponding to *h*-ZIS, Pt_0.3_-ZIS, and Pt_3.0_-ZIS exhibit little shift (Supplementary Fig. 19). The S *2p* spectrum for *h*-ZIS shows two peaks at 161.8 and 163.0 eV, respectively. After loading Pt single sites, a blue-shift of ~0.4 eV in Pt_0.3_-ZIS is observed, indicating that the electrons are transferred from Pt to *h*-ZIS and enriched around S atom. This also proves that the decoration of Pt single atoms would modulate the electronic structures of *h*-ZIS (Fig. 2a)^20,27^. With the increasing amount of Pt to 3.0 wt%, a smaller blue-shift of about 0.3 eV is detected. For the Pt *4* *f* spectra, Pt/C exhibits three peaks at 71.90, 71.77, and 73.19 eV, which correspond to the Pt^0^, Pt^2+^, and Pt^4+^ state, respectively (Fig. 2b)^16^. In contrast, the Pt_0.3_-ZIS mainly contains Pt^δ+^ species (72.10 eV), revealing the formation of a higher coordination number with the Pt–S bonds than the Pt–Pt bonds^28–30^. Interestingly, both Pt^0^ and Pt^δ+^ peaks appear in the spectrum of Pt_3.0_-ZIS (70.90 and 72.09 eV), which is probably owing to the well-constructed both Pt single atoms and nanoparticles^29^. The detailed information for XPS fits is listed in Supplementary Tables 2–5.

Furthermore, X-ray absorption near-edge structure (XANES) and extended X-ray absorption fine structure spectroscopy (EXAFS) were conducted to investigate the local atomic structure and electronic environment of Pt species in Pt-ZIS. EXAFS results in Fig. 2c show *k*^2^-weighted Fourier transforms from the Pt L_3_-edge EXAFS oscillations of Pt_1.4_-ZIS and Pt_3.0_-ZIS in comparison to that of Pt foil and PtO_2_ (*k*^2^-weighted χ(*k*) signals in Supplementary Fig. 20). The only prominent shell in Pt_1.4_-ZIS locating at near 2.0 Å without any Pt–Pt contribution in the range of 2–3 Å testifies the atomically dispersed Pt on *h*-ZIS^17,29^, whereas an additional peak at about 2.6 Å arises in Pt_3.0_-ZIS, closing to that of Pt–Pt contribution. To gain visual illustrations of Pt coordination conditions, wavelet transform (WT) of the *k*^2^-weighted EXAFS spectra, a reflection of structure information in the resolution of *R* space and *K* space, are shown in Fig. 2d. Pt_1.4_-ZIS exhibits the maximum WT intensity at 1.8–2.1 Å in *R* space and 3–6 Å in *k* space, ascribing to Pt–S bond in the first coordination shell^31,32^. While a new WT intensity maximum near 2.5–2.8 Å and 9–11 Å suggests the coexistence of Pt–S and Pt–Pt bonds in Pt_3.0_-ZIS. The Fourier-transform EXAFS curves and the corresponding fitting results in Supplementary Fig. 21 and Supplementary Table 6 give the Pt–S coordination number of 2.6 for Pt_1.4_-ZIS, implying a similar coordination condition of Pt–S_3_ in Pt_SS_-ZIS as depicted by HAADF-STEM image and DFT simulations. The XANES spectra of Pt L_3_-edge show that the white-line intensity of Pt_1.4_-ZIS is lower than that of PtO_2_, but higher than that of Pt_3.0_-ZIS and Pt foil, demonstrating the Pt atoms are in an oxidation state originating from covalent Pt–S bonds, which is consistent with XPS results (Supplementary Fig. 22)^19^.

Moreover, we investigated the CO adsorption behavior on different photocatalysts using Fourier-transform infrared (FTIR) spectroscopy to provide additional information about the dispersion and chemical state of Pt (Fig. 2e–g). For Pt_0.3_-ZIS, only a weak vibration band appears at 2093 cm^−1^ corresponding to CO adsorption on Pt^δ+^^33,34^. While the adsorption of CO also produces a strong vibration band at 2033 cm^−1^ and another weak band at 1961 cm^−1^ for Pt_3.0_-ZIS. The main band at 2033 cm^−1^ can be ascribed to linearly bonded CO on Pt^0^ sites, and the band at 1961 cm^−1^ is caused by CO adsorbed on the interface between Pt clusters and the support^33^. All these characterizations provide compelling evidence that our protocol affords Pt_0.3_-ZIS with only positively charged Pt single atoms, while Pt_3.0_-ZIS with both single atoms and Pt nanoparticles.

### Photocatalytic H_2_ evolution performances

**Fig. 3:**
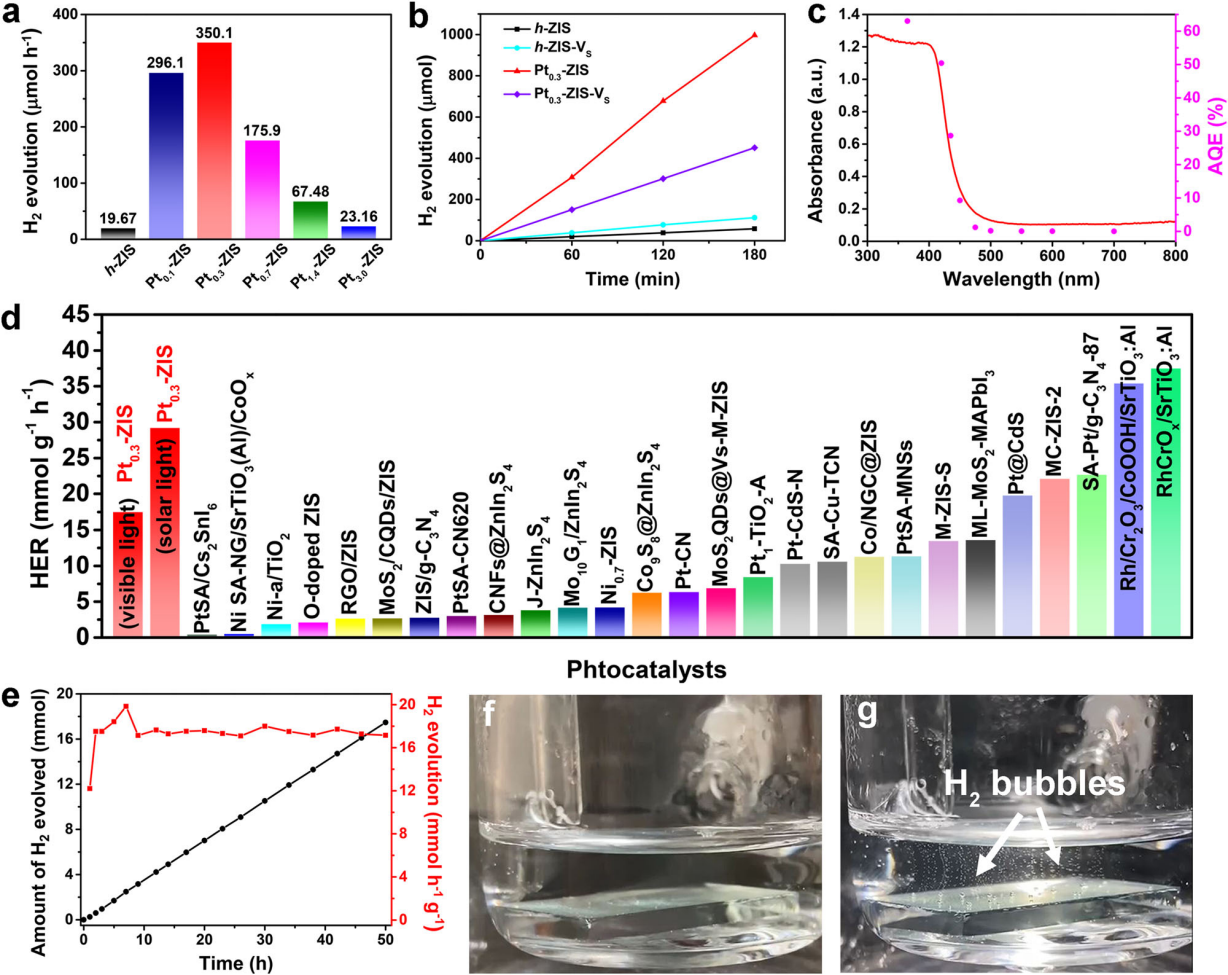
Evaluation of photocatalytic HER performances. **a** Visible light (*λ* > 420 nm) photocatalytic H_2_ evolution activities of *h*-ZIS and Pt-ZIS with different Pt loading content. **b** Visible light (*λ* > 420 nm) photocatalytic H_2_ evolution activity of Pt_0.3_-ZIS in comparison with *h*-ZIS, *h*-ZIS-V_S_, and Pt_0.3_-ZIS-V_S_. **c** Wavelength dependen**c**e of the AQE for Pt_0.3_-ZIS. **d** H_2_ evolution rates for Pt_0.3_-ZIS in this work compared with representative recently reported photocatalysts. **e** Cycling stability t**e**st of Pt_0.3_-ZIS. **f**, **g** Digital photograph of H_2_ evolution using Pt_0.3_-ZIS film without and with visible light irradiation.

With protrusion-shaped SSCs in hand, we next explored their photocatalytic HER activities in an aqueous solution with 10 vol% triethanolamine (TEOA) as the sacrificial agent under visible light (*λ* > 420 nm) irradiation. According to Fig. 3a, all Pt-loaded *h*-ZIS photocatalysts exhibit higher H_2_ evolution performance than the counterpart (*h*-ZIS: 19.67 μmol h^−1^ with 20 mg photocatalyst). The optimized rate (350.1 μmol h^−1^) is acquired at Pt_0.3_-ZIS, which is about 17.8 times enhanced than that of pristine *h*-ZIS. When Pt loading content exceeds 0.3 wt%, the activity experiences a decrease, and the catalytic efficiency of each Pt site is reduced (Supplementary Fig. 23). In addition, *h*-ZIS with sulfur vacancies (*h*-ZIS-V_S_) was synthesized through the treatment of NaBH_4_ in a water bath. Benefiting from the existence of localized states caused by sulfur vacancies, *h*-ZIS-V_S_ performs a narrower bandgap (2.66 eV) and a longer average fluorescence lifetime (5.86 ns) than *h*-ZIS (2.79 eV and 3.02 ns) (Supplementary Figs. 24 and 25). As a result, the photocatalytic activity of *h* -ZIS is enhanced after creating sulfur vacancies. Then Pt_0.3_-ZIS-V_S_ (0.28 wt% Pt) was also prepared by the same photochemical procedure (Supplementary Fig. 26). The obviously decreased ESR signal of Pt_0.3_-ZIS-V_S_ and the calculated adsorption energy reveal that Pt single atoms incline to be trapped at defect sites rather than protrude out of *h*-ZIS-V_S_ surface (Supplementary Figs. 26, 27). The recorded H_2_ generation rate for Pt_0.3_-ZIS-V_S_ is only about half of that for Pt_0.3_-ZIS (Fig. 3b). These results imply that the excellent catalytic activity of Pt_0.3_-ZIS could be mainly attributed to the protrusion-like Pt species on 2D *h*-ZIS. Similar to the trend of visible light, Pt_0.3_-ZIS also displays a boosted activity under simulated solar light with a total H_2_ generation of 3504 μmol within 6 h, whereas only 245.7 μmol H_2_ is formed by *h*-ZIS (Supplementary Fig. 28). Additionally, Pt_0.3_-ZIS could introduce acceptable HER performance even in pure water and the H_2_ evolution rate is ~24.04 μmol h^−1^ (1202 μmol h^−1^ g^−1^) under visible light irradiation (Supplementary Fig. 29). Dependence of apparent quantum efficiency (AQE) at each wavelength for Pt_0.3_-ZIS derived from the amount of generated H_2_ was estimated by various band-pass filters (Fig. 3c and Supplementary Table 7). The AQE matches well with the absorption spectrum of Pt_0.3_-ZIS, and reaches up to 50.4% at 420 nm. Experiments in dark or without photocatalysts show no H_2_ evolution, demonstrating that H_2_ is generated by the photocatalysis processes. Such high AQE and catalytic HER activity of Pt_0.3_-ZIS is far beyond the majority of representative photocatalysts (details see the comparisons in Fig. 3d and Supplementary Table 8). Furthermore, Pt_0.3_-ZIS almost maintains its photocatalytic H_2_ evolution rate at the initial level after continuous irradiation for 50 h (Fig. 3e). The characterizations including XRD, XPS, TEM, and HAADF-STEM, demonstrate that the structures undergo negligible changes, manifesting high stability of Pt_0.3_-ZIS (Supplementary Figs. 30–33).

Due to its outstanding performance, we dispersed Pt_0.3_-ZIS into ethanol solution and then drop-casted onto FTO substrate (1.5 × 2 cm^2^) to form a thin film (~3 μm thick) with excellent transmittance (Supplementary Figs. 34 and 35). As a proof-of-concept, the resultant Pt_0.3_-ZIS film was employed as a photocatalyst for H_2_ production. No H_2_ is generated before irradiation, while small H_2_ bubbles start to appear continuously when turning on the light (Fig. 3f, g and Supplementary Movies 1, 2). The H_2_ generation rate over the film achieves as high as 0.967 L h^−1^ m^−2^ (43.17 mmol h^−1^ m^−2^) under visible light irradiation, outperforming numerous recent reported photocatalysis films, such as C_3_N_4_ film (0.19 L h^−1^ m^−2^) and Pt_SS_-MOF (0.398 L h^−1^ m^–2^), which dictates an enormous potential for real applications^21,35^.

### Insight of the increased photocatalytic activity

**Fig. 4:**
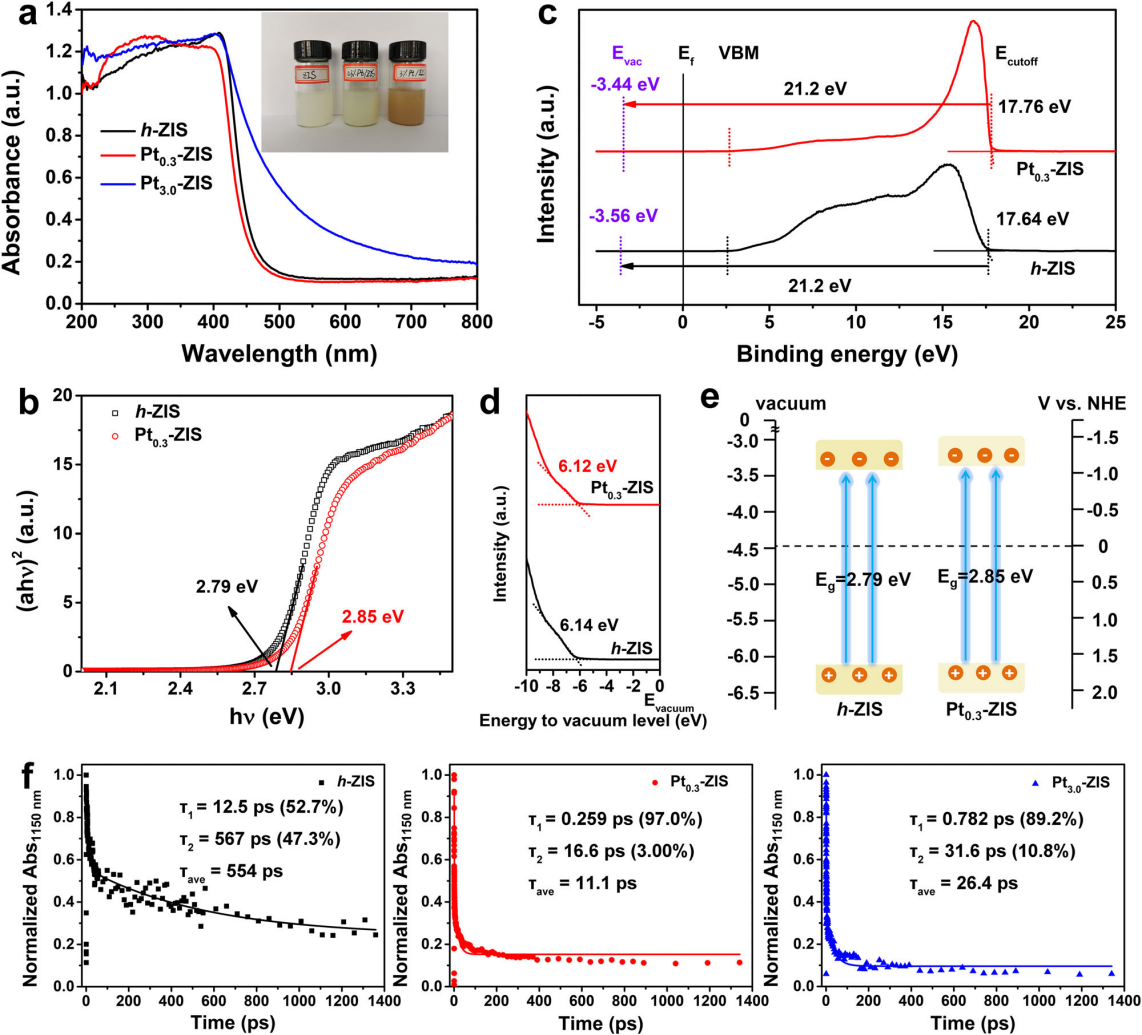
Mechanism insight the photocatalytic H_2_ evolution. **a** UV–vis diffuse reflectance spectra for *h*-ZIS, Pt_0.3_-ZIS, and Pt_3.0_-ZIS, insert: digital images for *h*-ZIS, Pt_0.3_-ZIS, and Pt_3.0_-ZIS, the concentration of suspensions is 2 mg mL^−1^. **b** Bandgap for *h*-ZIS and Pt_0.3_-ZIS. **c** Comparison and relationship of VBM, *E*_f_, and *E*_vac_ of UPS spectra. **d** UPS spectra of *h*-ZIS and Pt_0.3_-ZIS in the valence band region. **e** Schematic illustration of the band structure of *h*-ZIS and Pt_0.3_-ZIS. **f** Normalized time profiles of transient absorption at 1150 nm during the TDR of *h*-ZIS, Pt_0.3_-ZIS, and Pt_3.0_-ZIS.

To shed light on the origin of enhanced activity, three elementary processes in photocatalysis HER, namely light absorption, charge separation, and interfacial H_2_ catalysis, are taken into consideration. The optical absorption properties were examined by ultraviolet-visible (UV-vis) diffuse reflectance spectra. An absorption edge at around 440 nm corresponding to the bandgap of about 2.85 eV, is observed for Pt_0.3_-ZIS. It is slightly larger than that of pristine *h*-ZIS (2.79 eV), revealing a blue-shift absorption edge of *h*-ZIS upon Pt stabilizing (Fig. 4a and b). These results are similar to those achieved from the photocurrent action spectra of different photocatalysts film electrodes (Supplementary Fig. 36). Even though the light absorption is enhanced in the visible range with further increasing the amount of Pt (Pt_3.0_-ZIS), the measured photocurrent action spectrum of Pt_3.0_-ZIS is quite different from its UV-vis absorbance, in which there is almost no photocurrent at 500 nm. Additionally, the AQE of Pt_3.0_-ZIS was also recorded (Supplementary Fig. 37 and Table S7). Consistent with the photocurrent action, the AQE of Pt_3.0_-ZIS does not follow well with the UV-vis spectrum and only a little amount of H_2_ is generated at 500 nm. Considering that the Pt colloids exhibit broadband optical absorption from ultraviolet to the visible light region, we can conclude that Pt nanoparticles formed in Pt_3.0_-ZIS could extend the light absorption, but have limited contributions to the photocatalytic performances of *h*-ZIS^36^. The digital images demonstrate an obvious color change from ivory to pale yellow for *h*-ZIS and Pt_0.3_-ZIS, and finally to dark brown for Pt_3.0_-ZIS. The relationship between VBM, *E*_f_, and *E*_vac_, and the UPS spectra of different photocatalysts are shown in Fig. 4c. The vacuum level (*E*_vac_) should be located 21.2 eV above the cutoff energy (*E*_cutoff_) of the spectrum. The relative locations of valence band maximum (VBM) are calculated to be −6.14 eV (*h*-ZIS) and −6.12 eV (Pt_0.3_-ZIS) compared with E_vac_ according to UPS spectra (Fig. 4d). As a result, *h*-ZIS and Pt_0.3_-ZIS display the conduction band minimum (CBM) potential of −3.35 and −3.27 eV, respectively (Fig. 4e). The detailed band positions are illustrated in Supplementary Table 9. The elevation of CBM endows the photoexcited electrons in Pt_0.3_-ZIS with a higher reduction ability to react with hydrogen ions and form molecular hydrogen in HER compared with *h*-ZIS^4^. This favorable feature of the band structure is advantageous to prohibit the recombination of electron-hole pairs, and is responsible for the enhanced photocatalytic performance of Pt_0.3_-ZIS.

The electron dynamics involved in photocatalysis were revealed by time-resolved diffuse reflectance (TDR) spectroscopy, a robust technique to provide direct evidence for the effect of Pt loading on charge separation in semiconductors^37,38^. The pump light with a central wavelength of 420 nm was used, which is effective for photoinduced an interband transition in *h*-ZIS. It turns out that probing in the wavelength range of 900–1200 nm yielded similar TDR spectra, featuring free or trapped photoexcited electrons (Supplementary Fig. 38)^6,39,40^. And a set of representative data obtained at 1150 nm combined with the biexponential fitting results are illustrated. In Fig. 4f, the two-time constants for *h*-ZIS are τ_1_ = 12.5 ps (52.7%) and τ_2_ = 567 ps (47.3%), respectively, and the weighted average lifetime is 554 ps. In comparison, the characterized two-time constants for Pt_0.3_-ZIS (τ_1_ = 0.259 ps (97.0%) and τ_2_ = 16.6 ps (3.0%)) and Pt_3.0_-ZIS (τ_1_ = 0.782 ps (89.2%) and τ_2_ = 31.6 ps (10.8%)) lead to much shorter average lifetime of 11.1 and 26.4 ps, respectively. In general, the average recovery lifetime is considered as a crucial indicator for evaluating the separation efficiency of photoexcited electron-hole pairs, and such a shortened lifetime indicates the opening of an additional pathway for electron transfer after Pt deposition^41,42^. Based on the mean transient decay times of *h*-ZIS (*τ*_ZIS_), Pt_0.3_-ZIS (*τ*_0.3_), and Pt_3.0_-ZIS (*τ*_3.0_), we can determine the injection rate through the equation as ${k}_{{{{{{\rm{ET}}}}}}}=(1/{{\tau }}_{x})-(1/{{\tau }}_{{{{{{\rm{ZIS}}}}}}})$, where *x* represents the weight ratio of Pt^43,44^. It is calculated that the *k*_ET_ of Pt_0.3_-ZIS is 8.8 × 10^7^ s^−1^, to be ~2.4 times faster than that of Pt_3.0_-ZIS (3.6 × 10^7^ s^−1^). In addition, as a more significant parameter for photocatalytic activity, the efficiency of electron injection (*η*_inj_) from *h*-ZIS to Pt is calculated as ${{\eta }}_{{{{{{\rm{inj}}}}}}}=1-{{\tau }}_{x}/{{\tau }}_{{{{{{\rm{ZIS}}}}}}}$^43,44^, and Pt_0.3_-ZIS affords a higher *η*_inj_ (*η*_inj_ = 98.0%) than that of Pt_3.0_-ZIS (*η*_inj_ = 95.2%).

Additionally, steady-state photoluminescence (PL) spectra were recorded (Supplementary Fig. 39). Loading Pt onto *h*-ZIS results in significant PL quenching for Pt-ZIS, and Pt_0.3_-ZIS exhibits the lowest PL intensity among the photocatalysts, demonstrating an improvement in charge separation^45^. Concurrently, the photocurrent intensity of Pt_0.3_-ZIS is around 6.5 and 3.9 times compared with that of *h*-ZIS and Pt_3.0_-ZIS, respectively (Supplementary Fig. 40a). EIS Nyquist plots of *h*-ZIS, Pt_0.3_-ZIS, and Pt_3.0_-ZIS together with simulated equivalent electrical circuits are also provided in Supplementary Fig. 40b and c, respectively, in which *R*_ct_ is interfacial charge-transfer resistance^46^. Based on the model, Pt_0.3_-ZIS shows the smallest semicircle diameter and *R*_ct_ value (Supplementary Table 10), proving the lowest resistance of interfacial charge transfer in Pt_0.3_-ZIS^47^. The efficient charge separation in Pt_0.3_-ZIS was also confirmed by photocatalytic activation of peroxymonosulfate (PMS) to degrade antibiotic ornidazole (ONZ) pollutants (Supplementary Fig. 41). For Pt_0.3_-ZIS, the degradation efficiency of ONZ is 1.8 and 1.7 times and the utilization efficiency of PMS is 2.4 and 1.8 times than *h*-ZIS and Pt_3.0_-ZIS, respectively. The effective electrons injection from Pt_0.3_-ZIS to PMS molecules generate more reactive species, contributing to the higher photocatalytic performance than *h*-ZIS and Pt_3.0_-ZIS. These results disclose that more rapid and efficient directional migration of photogenerated electrons is realized by isolated Pt atoms decoration, partly accounting for Pt_0.3_-ZIS with the greatly enhanced photocatalytic performance^38,48^.

**Fig. 5:**
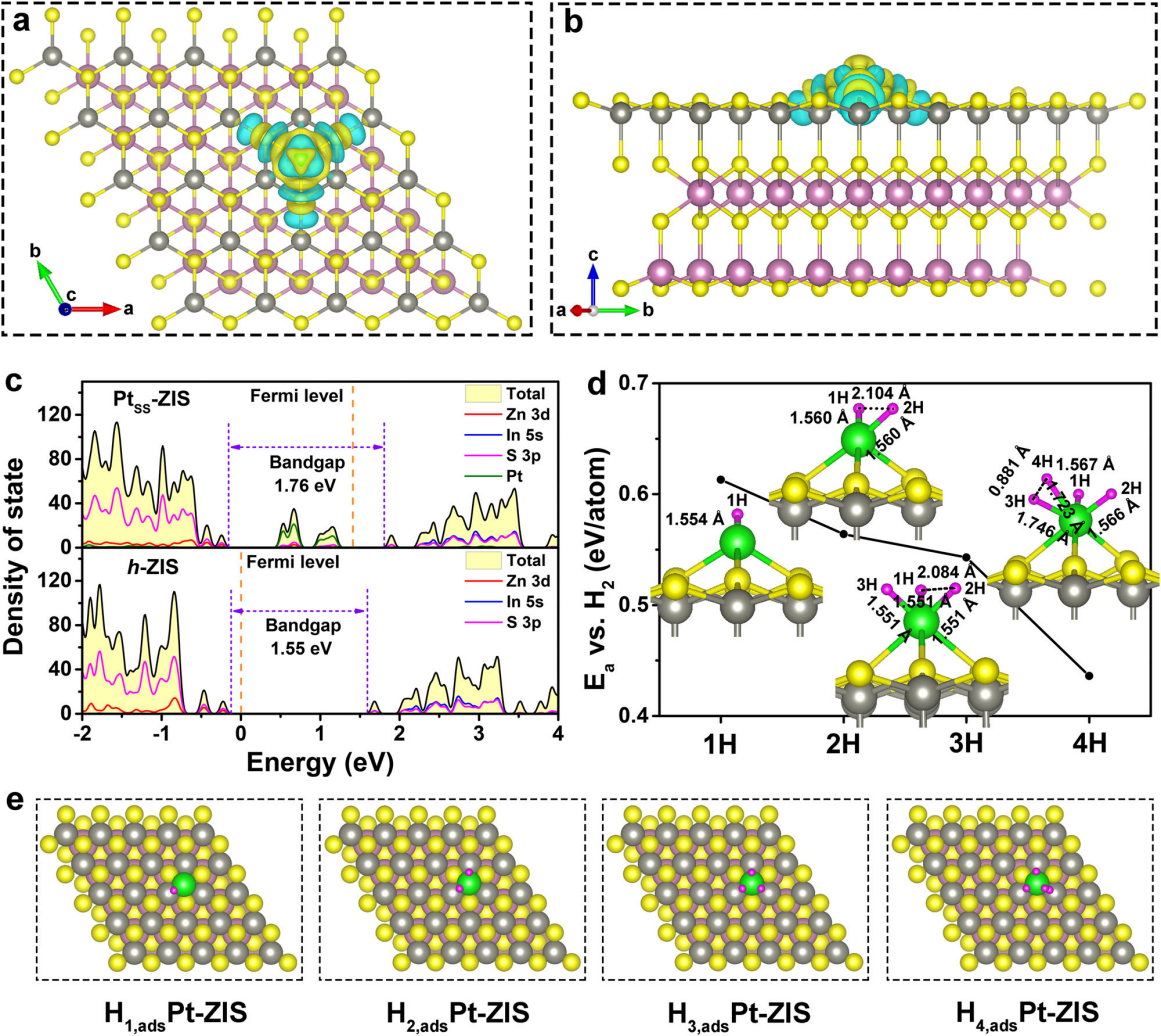
Density functional theory (DFT) calculations. **a** Top and **b** side view of calculated charge difference surfaces of Pt_SS_-ZIS with yellow and cyan colors represent positive and negative electron density isosurfaces, respectively. The value of isosurface is 0.002e/bohr^3^. **c** Density of states of the *h*-ZIS and Pt_SS_-ZIS. **d** Calculated adsorption energies of H atoms as a function of the H coverages (from one H atom to four H atoms) on the single Pt atom photocatalysts with *h*-ZIS as support. The H–H and Pt–H distances are shown in the figure. The adsorption energies (E_a_) were calculated by: ${{{{{{\rm{E}}}}}}}_{{{{{{\rm{a}}}}}}}=\left[{{{{{{\rm{E}}}}}}}_{{{{{{{\rm{Pt}}}}}}}_{{{{{{\rm{SS}}}}}}}-{{{{{\rm{ZIS}}}}}}}+\frac{{{{{{\rm{n}}}}}}}{2}{{{{{{\rm{E}}}}}}}_{{{{{{{\rm{H}}}}}}}_{2}}-{{{{{{\rm{E}}}}}}}_{{{{{{{\rm{Pt}}}}}}}_{{{{{{\rm{SS}}}}}}}-{{{{{\rm{ZIS}}}}}}+{{{{{\rm{nH}}}}}}}\right]/{{{{{\rm{n}}}}}}$. The yellow, gray, pink, green, and purple spher**e**s represent the S, Zn, In, Pt, and H atoms, respectively. **e** Side view of Pt_SS_-ZIS schematic structure with one H atom, two H atoms, three H atoms, and four H atoms chemisorbed on Pt atom, termed as H_1,ads_Pt-ZIS, H_2,ads_Pt-ZIS, H_3,ads_Pt-ZIS, and H_4,ads_Pt-ZIS, respectively.

DFT calculations were further carried out to dive fundamental insight into the effect of atomical Pt decoration. The charge density difference isosurface images reveal a strong charge redistribution at Pt-bonding region after the presence of protrusion-like single Pt atom on the basal plane of *h*-ZIS, and the calculated Bader charge shows that 0.06 *e* is transferred from Pt to S atoms in the *h*-ZIS substrate, confirming the strong interaction between Pt and *h*-ZIS (Fig. 5a and b)^30^. It is also observed that the Pt–S_3_ coordination has obvious charge transfer along the z direction. When the Pt adsorbate hybridizes with the *p* band of S, the adsorbate state split into localized bonding and antibonding states. In the projected density of states (PDOS) profile for Pt_SS_-ZIS, the dominant feature is Pt *5d*-S *3p* bonding resonances below the Fermi level and forming hybridized electronic states (Fig. 5c). Such states are considered as the electron acceptor states that could endow Pt_SS_-ZIS with metallic conductive character to inhibit the recombination of electron-hole pairs^49^. Moreover, the antibonding states of Pt_SS_-ZIS with the position all above the Fermi level involve in constructing conduction band, which probably leads to the upshift of CBM^19^. This enlarged bandgap for Pt_SS_-ZIS is consistent with the UV-vis absorption spectra and UPS spectra. Hence, theoretical calculations suggest that the covalent Pt–S coordination bond within Pt_SS_-ZIS forms additional charge-transfer channels to improve the charge mobility, causing an enhanced photocatalytic activity.

To disclose the underlying interfacial catalytic contribution of H_2_O to H_2_ from Pt single site, the adsorption of H atom on Pt atom was investigated. It is demonstrated that the adsorption strength of H decreases with an increase in the number of adsorbed H atoms, eventually leading to a minimum value when four H atoms are adsorbed (Fig. 5d). The single H atom exhibits adsorption energy (*E*_a_) of 0.613 eV, while it decreases to 0.564 eV per H in the case of two H atoms. As the number of interacting H atoms increases, the Pt to H interaction for bond formation becomes a compromise between the H–H electrostatic repulsion and the orbital hybridization of Pt–H^50^. Specifically, when three H atoms adsorb onto a single Pt atom, they locate symmetrically around Pt site, leading to corresponding adsorption energy of 0.543 eV per H. With the number of H atoms reaching four, the adsorption configuration is one in which a H_2_ dimer and two isolated H atoms are formed with an adsorption energy of 0.436 eV per H (Fig. 5d and e). The bond length of Pt–3H is elongated from 1.551 to 1.746 Å, and that of Pt–4H is 1.723 Å, which are much longer than that of Pt-1H (1.567 Å) and Pt-2H (1.566 Å) (the detailed distance of H–H atoms and Pt–H atoms are labeled in Fig. 5d). Moreover, the distance between 3H and 4H is only 0.881 Å, close to that in H_2_ molecule (0.740 Å). These phenomena indicate the weakened adsorption strength of 3H and 4H on Pt single site, thus promoting the desorption of 3H and 4H and ease of H_2_ production on Pt atom.

Finally, we performed the H adsorption free energy ($\Delta {G}_{{{{{{\rm{H}}}}}}}^{* }$) to examine the HER activity for different sites and the number of H atoms. Our simulations indicate that the adsorbed H on Pt, neighboring S, neighboring Zn, and second-neighboring Zn move to the tilted top site toward adjacent Zn atom on the surface after relaxation with an identical $\Delta {G}_{{{{{{\rm{H}}}}}}}^{* }$ of −0.36 eV, and the $\Delta {G}_{{{{{{\rm{H}}}}}}}^{* }$ of second-neighboring S is 0.61 eV (Supplementary Figs. 42, 43, and Table 11). Furthermore, the $\Delta {G}_{{{{{{\rm{H}}}}}}}^{* }$ gradually increases for the second (−0.26 eV) and third (−0.23 eV) adsorbed H atoms on Pt single site, and it becomes positive for the fourth adsorbed H atom (+0.10 eV) (Supplementary Fig. 44). Commonly, one material is regarded as a good HER catalyst when the value of $\Delta {G}_{{{{{{\rm{H}}}}}}}^{* }$ is close to thermoneutral ($\Delta {G}_{{{{{{\rm{H}}}}}}}^{* }$ ≈ 0)^51^. Therefore, we suggest that the Pt single atom would be the active site for HER and the catalyst that forms in situ states might be H_3,ads_Pt-ZIS^31^. DFT simulations were also carried out on Pt_SS_-ZIS-V_S_. Unlike Pt_SS_-ZIS, H atom tends to be adsorbed right above Pt atom after structural relaxation, suggesting that it is difficult for Pt atom in Pt_SS_-ZIS-V_S_ to adsorb more H atom (Supplementary Fig. 45). Additionally, the calculated $\Delta {G}_{{{{{{\rm{H}}}}}}}^{* }$ for H atom on Pt_SS_-ZIS-V_S_ is -0.22 eV, which is larger than that of H_3,ads_Pt_SS_-ZIS (+0.10 eV). These results prove the recently reported tip enhancement effect that protrusion-like single atoms could continuously enrich protons, which issues an improvement in proton mass transfer, thus boosting the kinetics of H_2_ production on the Pt single atom^22,26,50,52^. Active blocking experiment by introducing thiocyanate ion (SCN^−1^) into the catalyst system dictates a drastically decreased H_2_ generation rate from 350.1 to 27.44 μmol h^−1^ with the increase of KSCN concentration, confirming that Pt single atoms indeed serve as the centers for HER (Supplementary Fig. 46). Based on calculations, the adsorbed Pt single sites onto the surface of *h*-ZIS manifests a fast formation and release of molecular hydrogen, leading to an outstanding catalytic activity.

## Discussion

By combining experimental results with theoretical calculations, the high catalytic performance of Pt_0.3_-ZIS accompanied with long durability is confirmed. The enhanced H_2_ generation rate is due to the atomic protrusion-like Pt atoms with triple roles in the photocatalytic HER. First, single Pt atoms immobilized onto *h*-ZIS could tune the band structure of *h*-ZIS on upshifting the CBM, providing a larger reduction driving force. Second, the atomically dispersed Pt are acted as electron wells to accelerate charge separation and transportation. Third, the tridimensional protrusions induce effective proton mass transfer to the active Pt site and an almost thermoneutral $\Delta {G}_{{{{{{\rm{H}}}}}}}^{* }$ for HER, which is also supported by the smallest overpotential of Pt_0.3_-ZIS among *J–V* curves (Supplementary Fig. 47). A reasonable photocatalytic mechanism for HER from water is proposed (Supplementary Fig. 48). Upon light irradiation, the electron and hole pairs are generated and then migrate from the interior to the surface of *h*-ZIS. Due to the covalent Pt–S coordination bond, electrons are injected from the neighboring S atoms into Pt single atoms efficiently, followed by the reaction with adsorbed protons to generate H_2_. Simultaneously, the holes in *h*-ZIS are consumed by TEOA.

In summary, compared to the conventional defect-trapped-SSCs, atomically dispersed Pt sites are immobilized onto the basal plane of *h*-ZIS nanosheets to generate catalysts by a facile photochemical strategy. The efficient water reduction activity over Pt_0.3_-ZIS proceeds via regulated band structure, improved charge separation, reduced H_2_ evolution overpotential, and advanced protons mass transfer. The demonstration herein of constructing tridimensional protrusions through immobilizing ultralow content Pt SSCs onto 2D *h*-ZIS nanosheets presents a promising, cost- and energy-efficient avenue for boosting photocatalysis H_2_ evolution, and this prototype potentially would stimulate innovative ideas of enabling future ambient HER catalysts of industrial interest. The phenomenon of triggering tip enhancement by high-curvature nano-textures could function as a general prescription to enhance the performances of catalysts achieved in other reactions, such as organic pollutants degradation, O_2_ reduction, CO_2_ reduction, and N_2_ fixation.

## Methods

### Synthesis of hexagonal ZnIn_2_S_4_ (*h*-ZIS) nanosheets

In a typical synthesis, 68 mg ZnCl_2_ (Aladdin, 99.95%), 293 mg InCl_3_·4H_2_O (Aladdin, 99.9%), and 300 mg trisodium citrate (Aladdin, 99.0%) are dissolved into 25 mL of deionized water and 5 mL of glycol (Shanghai LingFeng Chemical Reagent Co. LTD., AR). After being drastically stirred for 30 min at room temperature, 150 mg thioacetamide (TAA, SCR, AR) is then added into the solution. After another 30 min stirring, the heterogeneous solution was transferred into a 50 mL Teflon-lined stainless steel autoclave and maintained at 120 °C for 12 h in an oven. After natural cooling, the products were collected by centrifugation, rinsed two times with ethanol and distilled water, and then freeze-dried.

### Synthesis of hexagonal ZnIn_2_S_4_ thin layers with S-vacancy (*h*-ZIS-V_S_)

The as-obtained *h*-ZIS (100 mg) was dissolved into 50 mL storage bottle containing 0.1 M NaBH_4_ (Sinopharm Chemical ReagentCo., Ltd, AR). The mixture was heated at 60 °C in a water bath. After 5 min, the resultant dispersion was centrifuged, and then freeze-dried.

### Synthesis of Pt-loaded *h*-ZIS and *h*-ZIS-V_S_ atomic layers (Pt-ZIS and Pt-ZIS-V_S_)

In a typical procedure of photochemical loading Pt on *h*-ZIS, 20 mg thin layers *h*-ZIS and different amounts of H_2_PtCl_6_ (4 mg mL^−1^) were dispersed in an aqueous solution containing 45 mL H_2_O and 5 mL triethanolamine (TEOA, XiLong Scientific, AR). Subsequently, the suspension is bubbled with argon gas through the reactor for 30 min to completely remove the dissolved oxygen and ensure that the reactor is in an anaerobic condition. The dispersion was kept stirring with a magnetic stirrer during visible light irradiation (*λ* > 420 nm). After the light treatment for 60 min, the sample was centrifuged and washed by deionized water twice and then freeze-dried. By alerting the volume of H_2_PtCl_6_ solution, the Pt content relative to *h*-ZIS was adjusted to about 0.1, 0.3, 0.7, 1.4, and 3.0 wt%, which were named as Pt_0.1_-ZIS, Pt_0.3_-ZIS, Pt_0.7_-ZIS, Pt_1.4_-ZIS, and Pt_3.0_-ZIS, respectively. For the synthesis of Pt_0.3_-ZIS-V_S_, the procedure was similar to that of Pt_0.3_-ZIS except changing *h*-ZIS to *h*-ZIS-V_S_.

### Preparation of Pt_0.3_-ZIS thin films

Typically, Pt_0.3_-ZIS (30 mg) powder was dispersed into ethanol (2 mL) and then sonicated for 10 min to obtain a colloidal solution. The film was prepared by drop-casting 400 μL of the colloidal solution onto roughened glass (1.5 × 2 cm^2^). Then the film was dried in a vacuum oven at the temperature of 60 °C.

### Photocatalytic hydrogen production

Twenty milligrams of photocatalysts was dispersed in 45 mL aqueous solution containing 10 vol% TEOA using an ultrasonic bath. Subsequently, the suspension was bubbled with argon gas through the reactor for 30 min to completely remove the dissolved oxygen and ensure that the reactor was in an anaerobic condition. The samples were irradiated under visible light using a 300 W Xenon lamp for H_2_ generation (PLS-SXE300D, Beijing Perfectlight Technology Co., Ltd, 300 mW cm^−2^). The reaction temperature is kept at about 8 °C. The visible light is filtered with a nominal 420 nm cutoff filter. The volume of H_2_ was measured by Shimadzu GC-8A gas chromatograph equipped with an MS-5A column and thermal conductivity detector. The apparent quantum efficiency (AQE) was calculated using the following equation,

1 $${{{{{\rm{AQE}}}}}}\,\left( \% \right)=\frac{{{{N}}}_{{{e}}}}{{{{N}}}_{{{p}}}}{{\times }}100 \% =\frac{2\times {{{n}}}_{{{{H}}}_{2}}\times {{{N}}}_{{{A}}}\times {{h}}\times {{c}}}{{{S}}\times {{P}}\times {{t}}\times {{\lambda }}}{{\times }}100 \%$$

where *N*_*p*_ is the total incident photons, *N*_*e*_ is the total reactive electrons, ${n}_{{H}_{2}}$ is the amount of H_2_ molecules, *N*_*A*_ is Avogadro constant, *h* is the Planck constant, *c* is the speed of light, *S* is the irradiation area, *P* is the intensity of irradiation light, *t* is the photoreaction time, and *λ* is the wavelength of the monochromatic light. For the stability test, the photocatalyst was continuously irradiated for 50 h. The turnover frequency (TOF) was calculated according to the following equation:

2 $${{{{{\rm{TOF}}}}}}=\frac{{n}_{({{{{{{\rm{H}}}}}}}_{2})}}{{n}_{({{{{{\rm{Pt}}}}}})}\cdot \tau }$$

## Supplementary information

Supplementary Information

Description of Additional Supplementary Files

Supplementary Movie 1

Supplementary Movie 2

## Data Availability

All data relevant to this study are available from the corresponding author upon reasonable request. The source data are provided as a Source data file. Source data are provided with this paper.

## Source data

Source Data
