# Supplementary material for: Protruding Pt single-sites on hexagonal ZnIn2S4 to accelerate photocatalytic hydrogen evolution
Source: Nat Commun. 2022 Mar 11;13:1287. doi: 10.1038/s41467-022-28995-1 (PMC8917206; doi:10.1038/s41467-022-28995-1)
Supplement: Supplementary file 1 — Supplementary Information [file 41467_2022_28995_MOESM1_ESM.pdf]

Supplementary Information for

# Protruding Pt single-sites on hexagonal $ZnIn_2S_4$ to accelerate photocatalytic hydrogen evolution

Shi et al.

# Table of Content

1. Supporting Texts ....................................................................................4

Characterizations and Measurements. ....................................................................4

Photoelectrochemical measurements. ....................................................................4

Time-resolved diffuse reflectance transient absorption measurements. ................5

In situ CO adsorption analysis. ....................................................................6

Photocatalytic activation of PMS for ONZ degradation. ....................................6

X-Ray absorption fine structure. ....................................................................6

Density functional theory (DFT) calculations. ....................................................7

2. Supplementary Figures ....................................................................................9

Supplementary Figure 1. ....................................................................9

Supplementary Figure 2. ....................................................................9

Supplementary Figure 3. ....................................................................10

Supplementary Figure 4. ....................................................................11

Supplementary Figure 5. ....................................................................11

Supplementary Figure 6. ....................................................................12

Supplementary Figure 7. ....................................................................12

Supplementary Figure 8. ....................................................................13

Supplementary Figure 9. ....................................................................14

Supplementary Figure 10. ....................................................................15

Supplementary Figure 11. ....................................................................16

Supplementary Figure 12. ....................................................................17

Supplementary Figure 13. ....................................................................17

Supplementary Figure 14. ....................................................................18

Supplementary Figure 15. ....................................................................19

Supplementary Figure 16. ....................................................................20

Supplementary Figure 17. ....................................................................22

Supplementary Figure 18. ....................................................................23

Supplementary Figure 19. ....................................................................24

Supplementary Figure 20. ....................................................................25

Supplementary Figure 21. ........................................................................ 25
Supplementary Figure 22. ........................................................................ 26
Supplementary Figure 23. ........................................................................ 26
Supplementary Figure 24. ........................................................................ 27
Supplementary Figure 25. ........................................................................ 28
Supplementary Figure 26. ........................................................................ 29
Supplementary Figure 27. ........................................................................ 30
Supplementary Figure 28. ........................................................................ 31
Supplementary Figure 29. ........................................................................ 31
Supplementary Figure 30. ........................................................................ 32
Supplementary Figure 31. ........................................................................ 32
Supplementary Figure 32. ........................................................................ 33
Supplementary Figure 33. ........................................................................ 33
Supplementary Figure 34. ........................................................................ 34
Supplementary Figure 35. ........................................................................ 34
Supplementary Figure 36. ........................................................................ 35
Supplementary Figure 37. ........................................................................ 36
Supplementary Figure 38. ........................................................................ 36
Supplementary Figure 39. ........................................................................ 37
Supplementary Figure 40. ........................................................................ 37
Supplementary Figure 41. ........................................................................ 38
Supplementary Figure 42. ........................................................................ 39
Supplementary Figure 43. ........................................................................ 40
Supplementary Figure 44. ........................................................................ 40
Supplementary Figure 45. ........................................................................ 41
Supplementary Figure 46. ........................................................................ 41
Supplementary Figure 47. ........................................................................ 42
Supplementary Figure 48. ........................................................................ 42
3. Supplementary Tables ........................................................................ 43
Supplementary Table 1 ........................................................................ 43
Supplementary Table 2 ........................................................................ 44

Supplementary Table 3.......................................................................................44
Supplementary Table 4.......................................................................................45
Supplementary Table 5.......................................................................................45
Supplementary Table 6.......................................................................................46
Supplementary Table 7.......................................................................................46
Supplementary Table 8.......................................................................................47
Supplementary Table 9.......................................................................................49
Supplementary Table 10.....................................................................................49
Supplementary Table 11.....................................................................................50
4. Supplementary References.....................................................................................51

# 1. Supporting Texts

**Characterizations and Measurements.** *h*-ZIS and Pt-ZIS were characterized using X-ray diffraction (XRD, Rigaku Rint-2500, CuKα source), transmission electron microscopy (TEM, Tecnai G2 F30), and high-resolution TEM (TEM, Tecnai G2 F30). High-angle annular dark-field scanning TEM (HAADF-STEM) observations were performed by FEI Themis Z. The absorption properties are obtained from UV–vis diffuse reflectance spectra, which the photocatalysts powders were put on a JASCO V-570 UV-visible/NIR spectrophotometer in absorption mode with the white standard of $BaSO_4$ as a reference. X-ray photoelectron spectroscopy (XPS) was performed with a Kratos AXIS Ultra DLD spectrometer. The adventitious carbon (C 1s located at 284.6 eV) was used as reference to binding energy. UPS spectra were measured using He I excitation (21.2 eV) and recorded with a constant pass energy of 1 eV in the ultrahigh vacuum (UHV) chamber of the XPS instrument. The weight percentage of Ni was measured by inductively coupled plasma by PerkinElmer NexION 300X. Atomic force microscopy (AFM) study was carried out on a Bruker BioScope Resolve bio-AFM with advanced correlation stage. Raman spectra was recorded by HR Evolution (HORIBA Scientific). The contents of the metals were measured by inductively coupled plasma optical emission spectrometry (ICP-OES, Agilent 720ES). The sulfur vacancy was recorded by ESR (Bruker EMXplus). Time-revolved photoluminescence spectra were performed on Edinburgh FS5 spectrophotometer. The concentration of ONZ and the degradation products were determined by the high-performance liquid chromatography (HPLC, Shimadzu LC-16) and liquid chromatography/time-of-flight/mass spectrometer (LC-TOF-MS, AB Sciex, LC-Triple-TOF5600).

**Photoelectrochemical measurements.** Electrochemical and photoelectrochemical measurements were performed in three-electrode quartz cells. Platinum wire was used as the counter electrode, and Ag/AgCl electrode was used as the reference electrode. For loading sample film electrodes on glassy carbon, 4 mg *h*-ZIS or Pt-ZIS was added into solution containing $H_2O$ and $CH_3CH_2OH$ with volume ratio of 1:1. Then 50 μL Nafion (Aladdin) was

added into the solution. After ultrasonic vibration for 10 min, 3 μL solution was taken out and drop on the surface of glassy carbon. The electrode was used for photoelectrochemical measurements after drying. 0.5 M $Na_2SO_4$ (Shanghai LingFeng Chemical Reagent Co. LTD., 99.0%) aqueous solution was used as the electrolyte. Electrochemical impedance spectroscopy (EIS) measurements were carried out in the same three-electrode system over a range from 0.01 to $1\times10^5$ Hz with an AC amplitude of 5 mV. The potential (vs. Ag/AgCl) was converted to the reversible hydrogen electrode (RHE) according to the Nernst equation:

$$E_{RHE} = E + 0.05916pH + E_0 \quad (1)$$

where $E_{RHE}$ was the potential vs. RHE, $E_0 = 0.1976$ V at 25 °C, and $E$ was the measured potential vs. Ag/AgCl.

The photocurrent action spectra of photocatalysts under different incident light wavelength were recorded through the home-made photoelectrochemical system under onset potential (Newport CS260 monochromator and CHI660E electrochemical workstation). The slit width of monochromator was set to be 2 nm, and the sweep rate was 2 nm $s^{-1}$. The incident photo to current conversion efficiency (IPCE) was calculated using the following equation:

$$IPCE\% = (I_{photo} \times 1240)/(\lambda \times I_0) \quad (2)$$

where $I_{photo}$ is the photocurrent density and $I_0$ is the intensity of incident light.

**Time-resolved diffuse reflectance transient absorption measurements.** The femtosecond diffuse reflectance transient absorption spectra were measured by the pump and probe method using a regeneratively amplified titanium sapphire laser (Spectra-Physics, Spitfire Pro F, 1kHz) pumped by a Nd:YLF laser (Spectra-Physics, Empower 15). The seed pulse was generated by a titanium sapphire laser (Spectra-Physics, Mai Tai VFSJW; fwhm 80 fs). Second harmonic oscillation of the output (420 nm, 4 μJ $pulse^{-1}$) was used as the excitation pulse. A white light continuum pulse, generated by focusing the residual of the fundamental light on a sapphire crystal after the computer controlled optical delay, was divided into two parts and used as the probe and the reference lights, of which the latter was used to compensate the laser fluctuation. Both probe and reference lights were directed to the sample powder coated on the FTO substrate,

and the reflected lights were detected by a linear InGaAs array detector equipped with the polychromator (Solar, MS3504). All measurements were carried out at room temperature.

**In situ CO adsorption analysis.** For $h$-ZIS, $Pt_{0.3}$-ZIS, and $Pt_{3.0}$-ZIS, the photocatalysts were pretreated with 20 mL $min^{-1}$ of Ar at 100 °C for 1 h, and then cooled down naturally. The CO adsorption on these samples was performed at 25 °C. 1% CO/Ar was introduced into the FTIR cell at a flow rate of 40 mL $min^{-1}$. After CO saturation, a Ar purge at a flow rate of 20 mL $min^{-1}$ was performed to remove gas phase CO from the cell. A temperature programmed desorption process was carried out to completely remove CO adsorption on photocatalysts. All the spectra were recorded using 64 scans with a resolution of 4 $cm^{-1}$.

**Photocatalytic activation of PMS for ONZ degradation.** In a typical experiment, 4 mg catalyst (0.2 g $L^{-1}$) was dispersed in 20 mL ONZ (5 mg $L^{-1}$) solution, and then for stirring with 30 min in dark. Next, 10 mg PMS (0.5 g $L^{-1}$) was added to the suspension and it was irradiated with visible light by using 300 W Xenon lamp (PLS-SXE300D, Beijing Perfectlight Technology Co., Ltd) with a 420 nm cut-off filter. During experiments, 0.5 mL suspension was collected at a certain time interval and added in 0.5 mL of $Na_2S_2O_3$ (2 mM) solution. The concentration of ONZ was measured by HPLC (Shimadzu, LC-16). To measure the utilization of PMS, the PMS concentration was analyzed by a UV–vis spectrophotometer (Shimadzu, UV-1800) equipped with quartz cuvettes of 1 cm light path, the test wavelength was 352 nm. The PMS utilization as the stoichiometric efficiency was calculated by correlating the PMS decomposition and ONZ degradation performance with stoichiometric efficiency= $\Delta[ONZ]/\Delta[PMS]$, where Δ[ONZ] and Δ[PMS] are the total decomposed amounts of ONZ and PMS, respectively.

**X-Ray absorption fine structure.** The X-ray absorption fine structure spectra were collected at TLS07A1 station in Taiwan National Synchrotron Radiation Research Center (NSRRC) in fluorescence mode at room temperature using a Si (111) double-crystal monochromator. The storage ring of BSRF was operated at 1.5 GeV with a maximum current of 250 mA in decay mode. The data collection was carried out in transmission mode using ionization chamber. The

X-ray absorption fine structure (XAFS) raw data were background-subtracted, normalized, and Fourier transformed by the standard procedures with the ATHENA program implemented in the IFEFFIT software packages[1]. To obtain the quantitative structural parameters around central atoms, least-squares curve parameter fitting analysis of the EXAFS $\chi(k)$ data was performed using the ARTEMIS module of IFEFFIT software packages. All fits are performed in the $R$ space with $k$-weight of 2.

**Density functional theory (DFT) calculations.** All calculations were performed by using the density functional theory (DFT) within the generalized-gradient approximation (GGA) with the exchange-correlation functional of Perdew-Burke-Ernzerhof (PBE). This has been implemented in the Vienna Ab Initio Simulation Package (VASP), which spans reciprocal space with a plane-wave basis and uses the projector-augmented wave (PAW) method[2,3]. A cutoff of $E_{cut}$ = 500 eV, energy convergence of $1\times10^{-4}$ eV, forced convergence of 0.01 eV $A^{-1}$, and Monkhorst–Pack k-point mesh of 2×2×1 have been used. The slab model was used for all computations. A vacuum of approximately 15 Å was placed above the slabs to separate the interaction between periodic images. For the $ZnIn_2S_4$ (001) surface slab model, a 4×4 single atomic layer with 64 S atoms, 16 Zn atoms and 32 In atoms was used. During the relaxation, all atomic positions were allowed to relax. For the Pt-$ZnIn_2S_4$ (001) slab model, in which Pt was placed above different atom, a 3×3, 4×4, and 5×5 $h$-ZIS supercell was used as the substrates to present different Pt coverage, corresponding to 5.12%, 2.88%, and 1.84%, respectively. During the relaxation, the Pt position and the surface region of $h$-ZIS were allowed to relax. To further determine the favorable atomic adsorption site of Pt on $h$-ZIS, the adsorption energy ($E_{ads\text{-}Pt}$) is calculated as:

$$E_{\mathrm{ads-Pt}} = E_{(h-\mathrm{ZIS})} + E_{\mathrm{Pt}} - E_{(\mathrm{Pt_{SS}-ZIS})} \quad (3)$$

where $E_{(\mathrm{Pt_{SS}-ZIS})}$ is the total energy for $h$-ZIS with adsorbed Pt atom, $E_{(h-\mathrm{ZIS})}$ is the energy for $h$-ZIS, and $E_{\mathrm{Pt}}$ is the energy of single Pt atom[4].

The adsorption energy of Pt at sulfur vacancy is calculated as:

$$E_{\mathrm{ads-Pt}} = E_{(h-\mathrm{ZIS-V_S})} + E_{\mathrm{Pt}} - E_{(\mathrm{Pt_{SS}-ZIS-V_S})} \quad (4)$$

where $E_{(\mathrm{Pt_{SS}-ZIS-V_S})}$ is the total energy for *h*-ZIS-$V_S$ with adsorbed Pt atom, $E_{(h-\mathrm{ZIS-V_S})}$ is the energy for *h*-ZIS-$V_S$, and $E_{\mathrm{Pt}}$ is the energy of single Pt atom.

The descriptor of HER activity, the adsorption free energy of $H^*$ ($\Delta G_{\mathrm{H}}^*$), can be obtained by correcting the adsorption energy of $H^*$. Differential binding energy was used to describe the stability of H atoms, defined by:

$$\Delta E_{\mathrm{H}} = E(\mathrm{ZnIn_2S_4} + \mathrm{n\ H}) - E(\mathrm{ZnIn_2S_4} + \mathrm{(n\text{-}1)\ H}) - 1/2E(\mathrm{H_2}) \quad (5)$$

where $E(ZnIn_2S_4$ + n H) is the total energy of the $ZnIn_2S_4$ photocatalyst with n H atoms, $E(ZnIn_2S_4$ + (n-1) H) represents the total energy of the photocatalyst with (n-1) H atoms, and $E(H_2)$ is the energy of a gas phase hydrogen molecule. For metal catalysts, $\Delta G_{\mathrm{H}}^*$ was used to calculate:

$$\Delta G_{\mathrm{H}}^* = \Delta E_{\mathrm{H}} + \Delta \mathrm{ZPE\text{-}T}\Delta \mathrm{S} \quad (6)$$

where ΔZPE and ΔS are the zero point energy change and entropy change of adsorption H, respectively.

## 2. Supplementary Figures

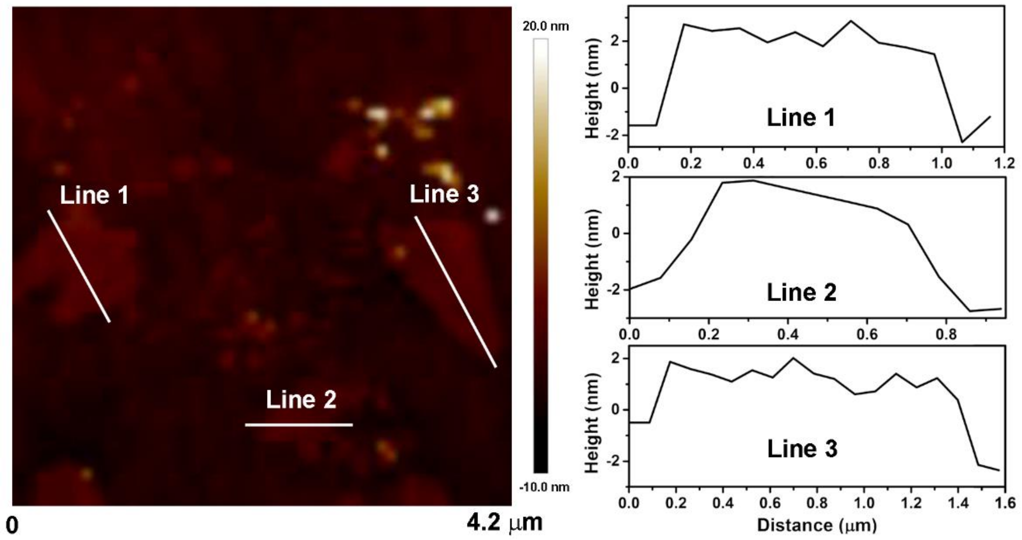


**Supplementary Figure 1.** AFM image and the corresponding height profiles of pristine *h*-ZIS material.

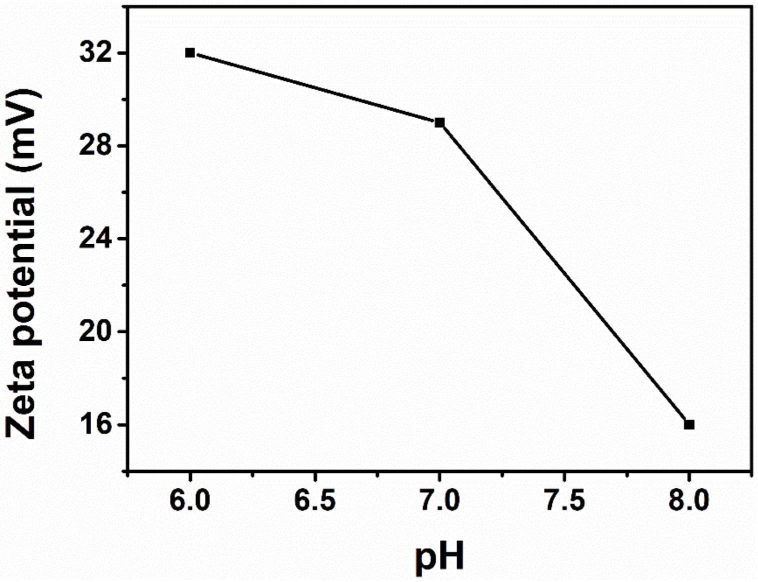


**Supplementary Figure 2.** Zeta potential of *h*-ZIS nanosheets dispersed in water of different pH values, showing a positive surface potential in a neutral environment.

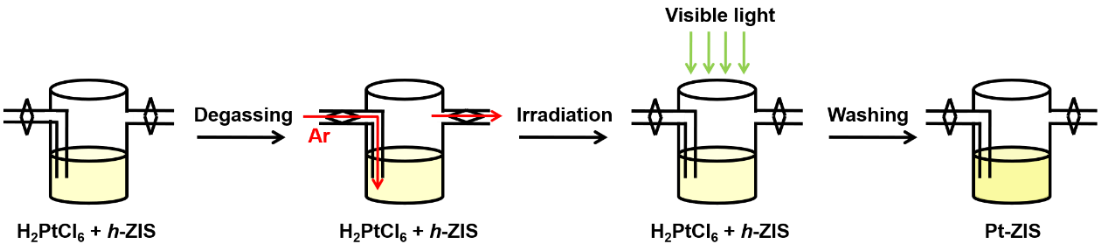


**Supplementary Figure 3. Scheme for the synthesis of Pt-ZIS.** $H_2PtCl_6$ was introduced into *h*-ZIS dispersion solution to allow the adsorption of Pt species. Then the mixture was irradiated by the visible light ($\lambda$ > 420 nm). After irradiation for 60 min, the catalyst was collected and washed thoroughly with water.

Once exposed to visible light, electron-hole pairs were generated on *h*-ZIS nanosheets. Electrons would transfer to the surface of *h*-ZIS and reduce Pt species. When the concentration of $H_2PtCl_6$ was low, Pt atom preferred to bond with three sulfur atoms to form a Pt-$S_3$ structure, as supported by the DFT simulation. While with the increasing of $H_2PtCl_6$, Pt nanoclusters or nanoparticles were generated.

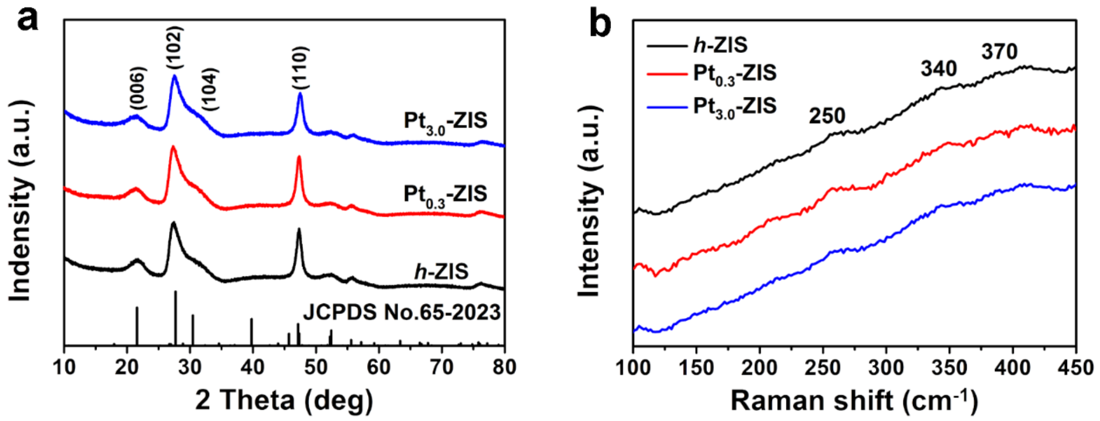


**Supplementary Figure 4. Structure characterizations of photocatalysts.** (a) XRD patterns and (b) Raman spectra of *h*-ZIS, $Pt_{0.3}$-ZIS, and $Pt_{3.0}$-ZIS. The XRD peaks at 21.7°, 27.7°, 30.4°, and 47.2° correspond to the (006), (102), (104), and (110) planes of hexagonal ZIS, respectively[5]. The vibrational modes located at 250, 340, and 370 $cm^{-1}$ are attributed to the longitudinal optical mode ($LO_1$), longitudinal optical mode ($LO_2$), and $A_{1g}$ mode of crystalline *h*-ZIS, respectively[6].

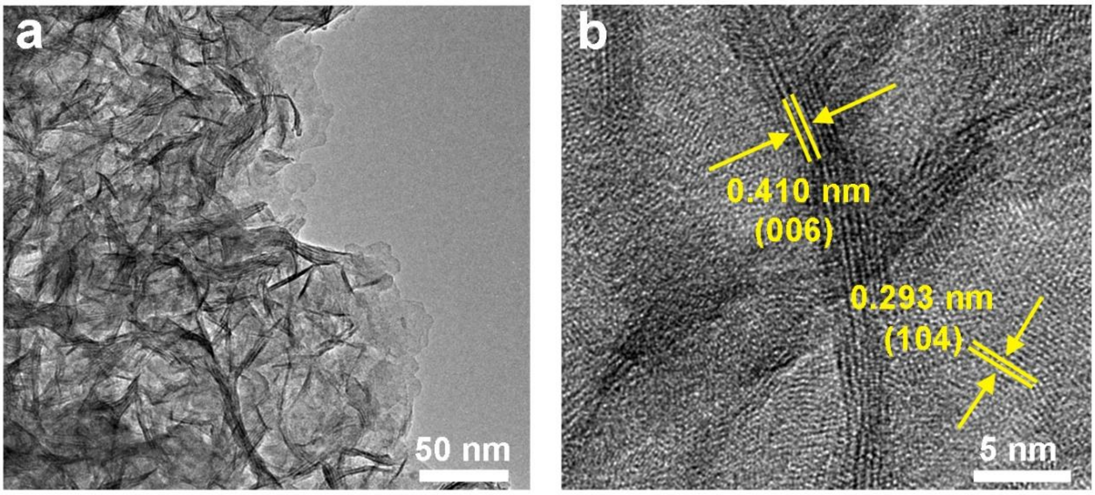


**Supplementary Figure 5. Morphology of *h*-ZIS.** (a) TEM and (b) HRTEM images of pristine *h*-ZIS.

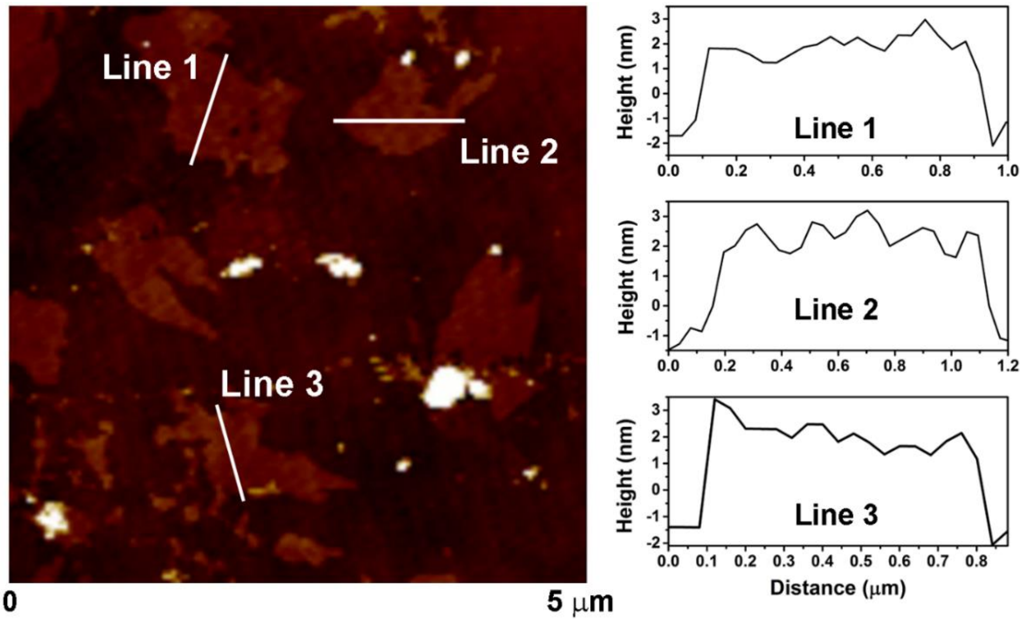


**Supplementary Figure 6.** AFM image and the corresponding height profiles of $Pt_{0.3}$-ZIS photocatalyst.

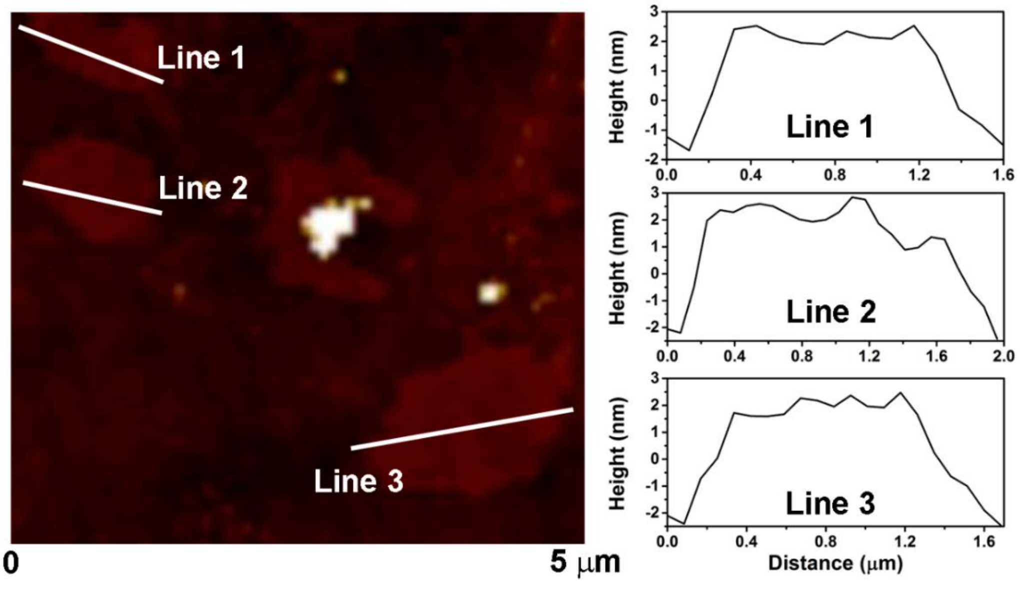


**Supplementary Figure 7.** AFM image and the corresponding height profiles of $Pt_{3.0}$-ZIS photocatalyst.

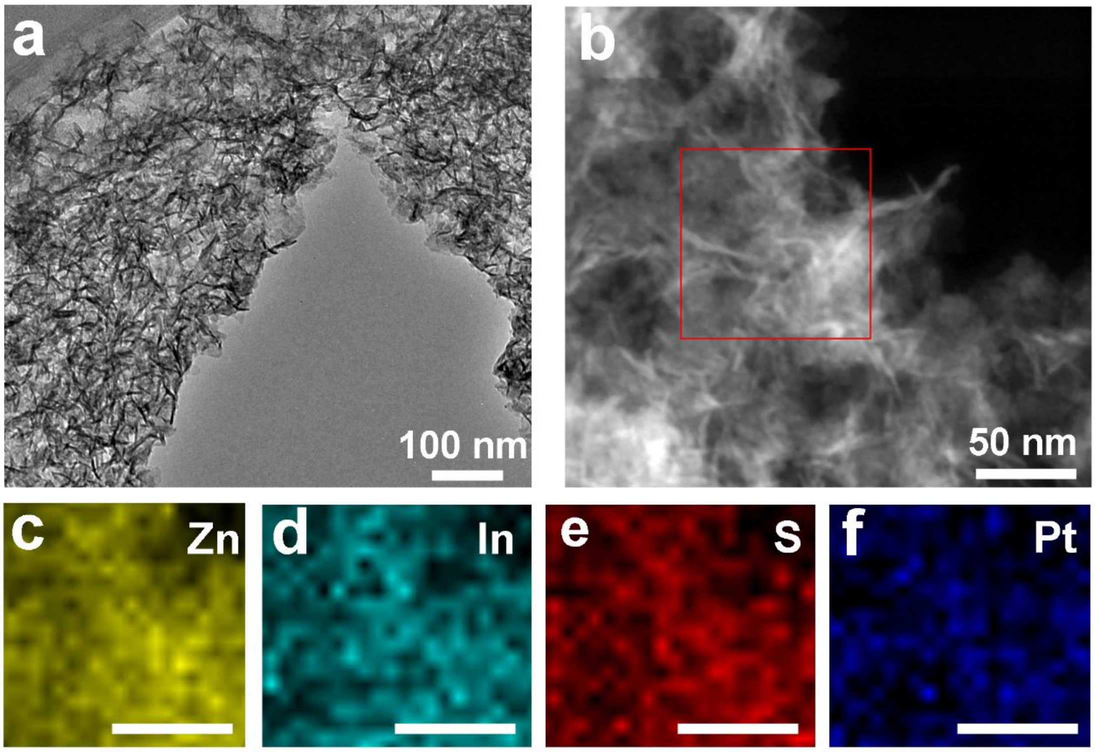


**Supplementary Figure 8.** (a, b) TEM images and (c-f) element mapping for Zn, In, S, and Pt elements of $Pt_{0.1}$-ZIS. The scale bar is 50 nm.

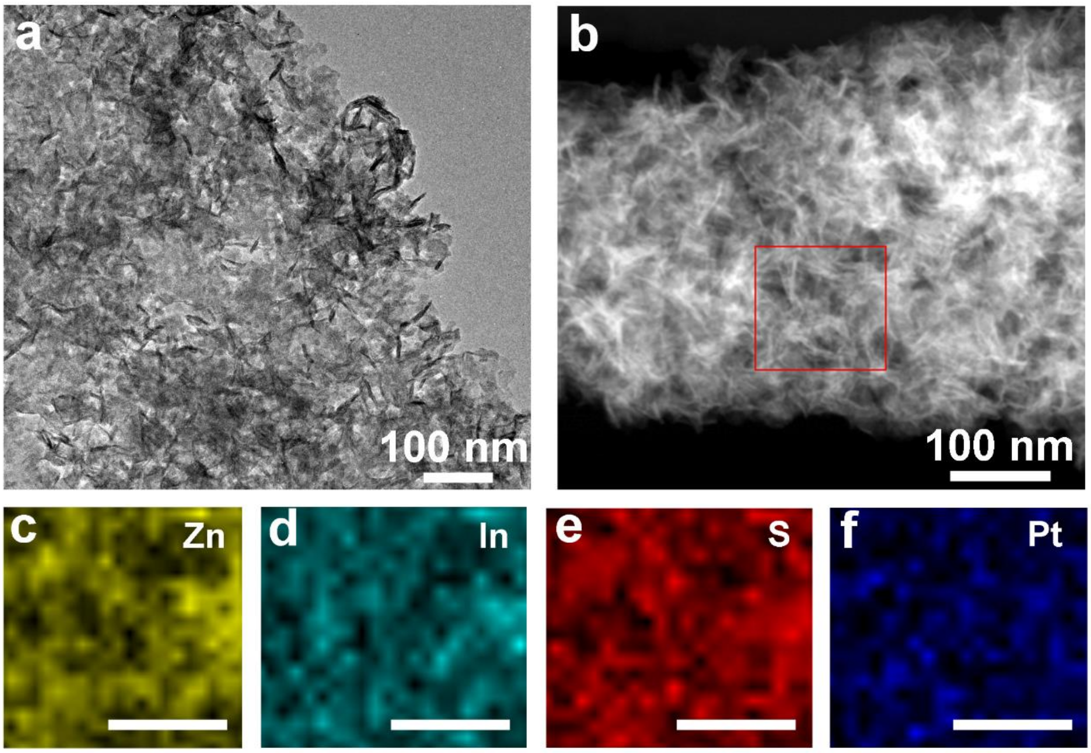


**Supplementary Figure 9.** (a, b) TEM images and (c-f) element mapping for Zn, In, S, and Pt elements of $Pt_{0.7}$-ZIS. The scale bar is 50 nm.

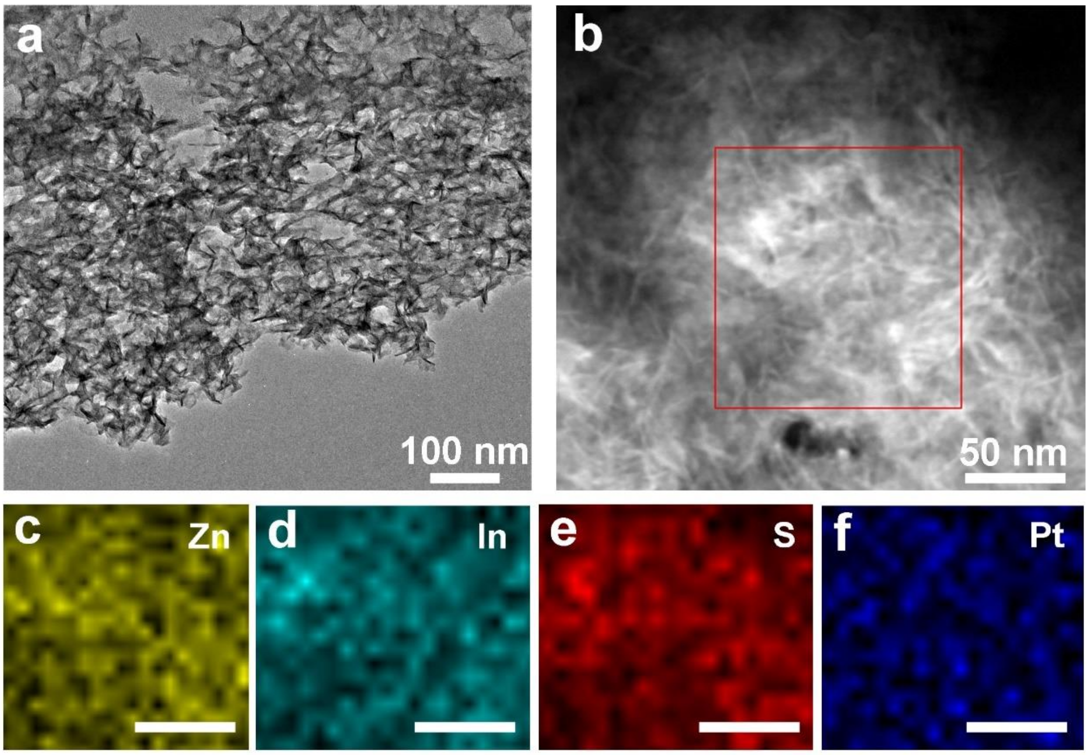


**Supplementary Figure 10.** (a, b) TEM images and (c-f) element mapping for Zn, In, S, and Pt elements of $Pt_{1.4}$-ZIS. The scale bar is 50 nm.

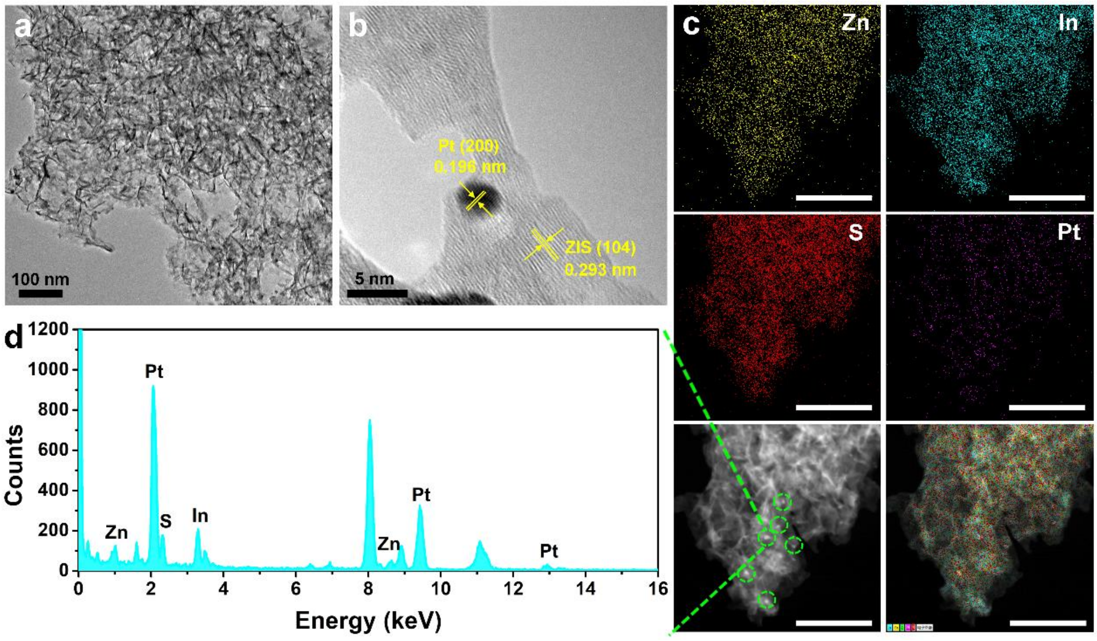


**Supplementary Figure 11.** (a) TEM and (b) HRTEM image of $Pt_{3.0}$-ZIS. (c) Elemental mapping of Zn, In, S, and Pt. (d) Corresponding EDS-point spectrum shown in green circle. The scale bar is 100 nm.

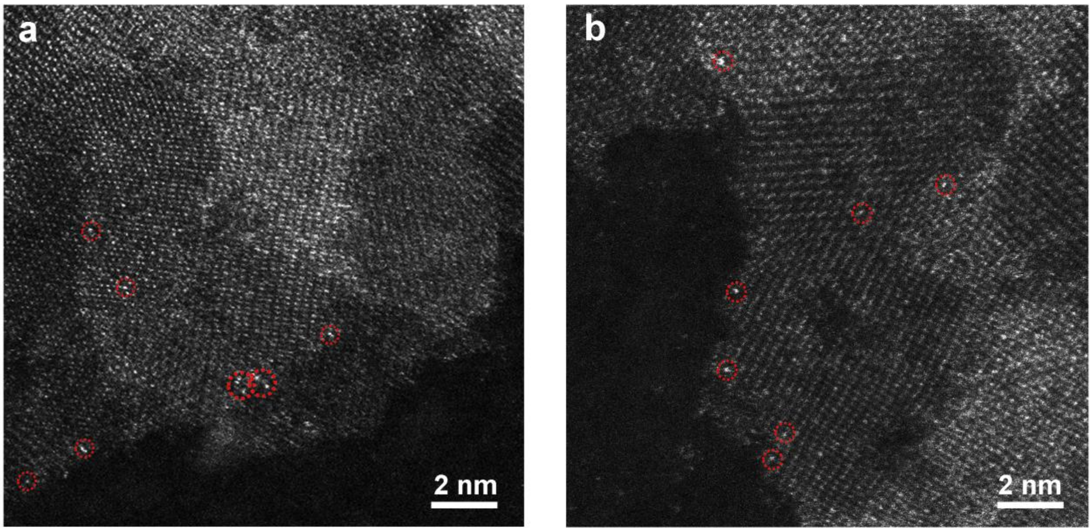


**Supplementary Figure 12.** (a, b) The additional HAADF-STEM images of $Pt_{0.3}$-ZIS. The images clearly show the intact structure and atomic dispersed Pt on the surface of *h*-ZIS.

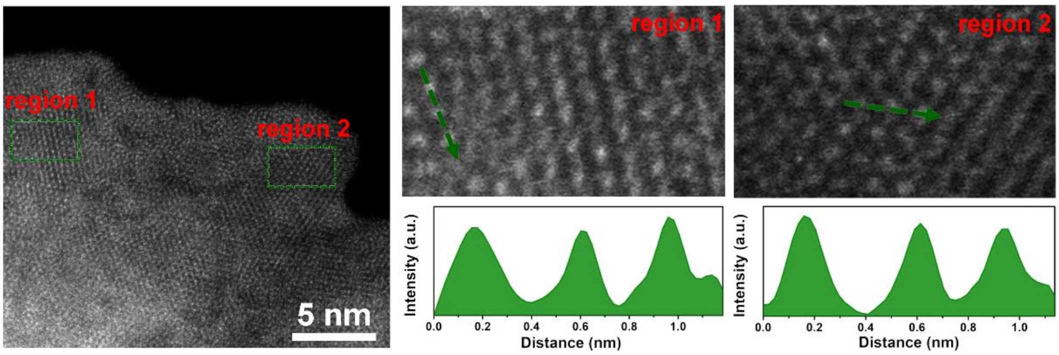


**Supplementary Figure 13.** HAADF-STEM image of pristine *h*-ZIS, and strength profiles from the areas labeled by green line.

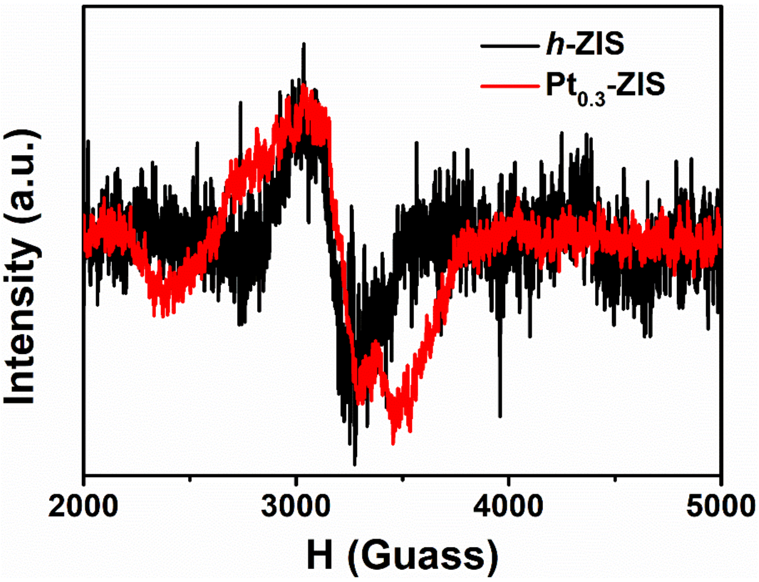


**Supplementary Figure 14.** ESR spectra of *h*-ZIS and $Pt_{0.3}$-ZIS.

ESR spectra of pristine *h*-ZIS and $Pt_{0.3}$-ZIS were utilized to investigate the sulfur vacancies before and after Pt single-sites loading. The almost similar intensities of two spectra indicate that the ZIS nanosheets keep an intact structure with little sulfur vacancies and thus Pt single atoms could not be immobilized at the sulfur vacancies.

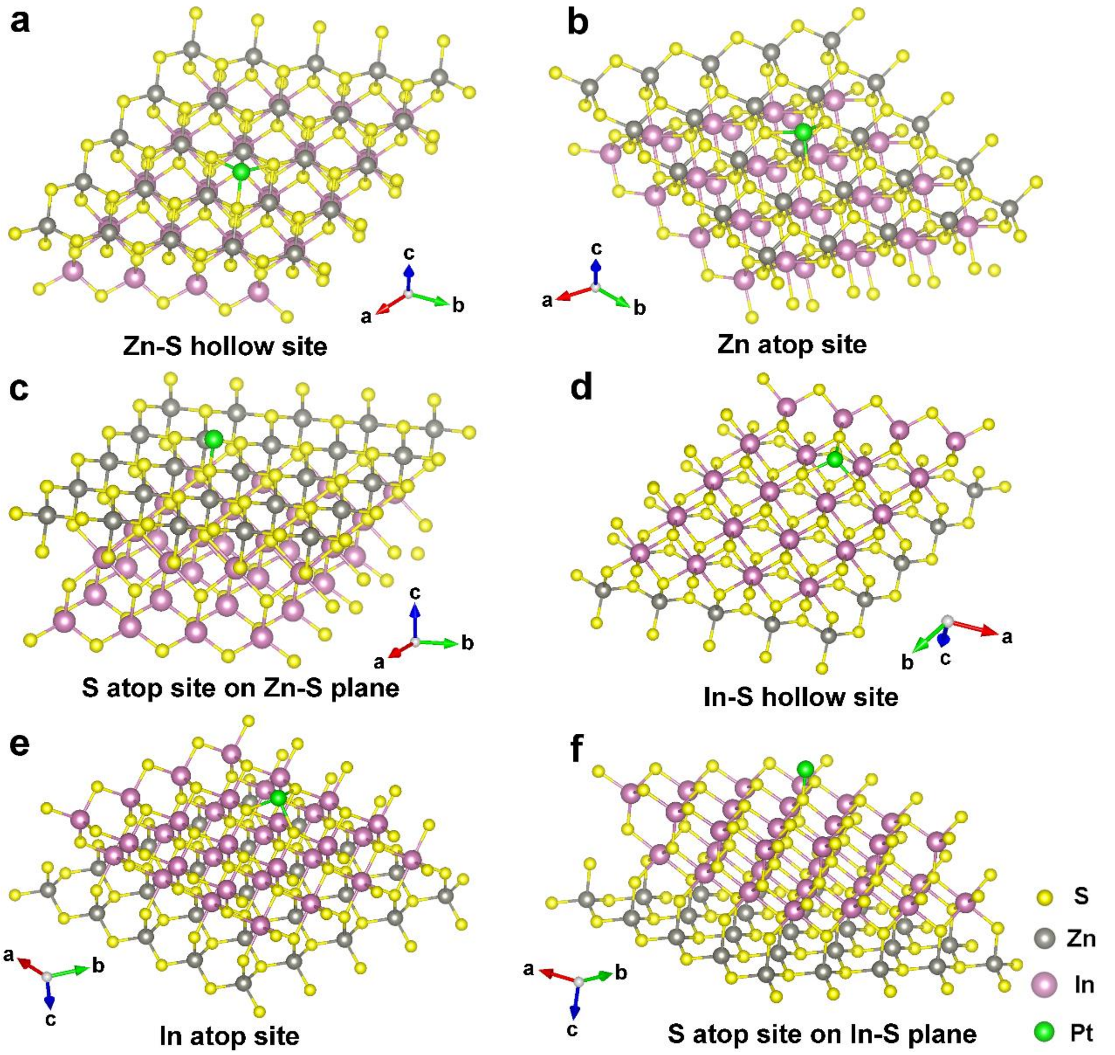


**Supplementary Figure 15.** A single Pt atom chemisorbs on Zn-S hollow site (a), Zn atop site (b), S atop site on Zn-S plane (c), In-S hollow site (d), In atop site (e), and S atop site on In-S plane (f), respectively.

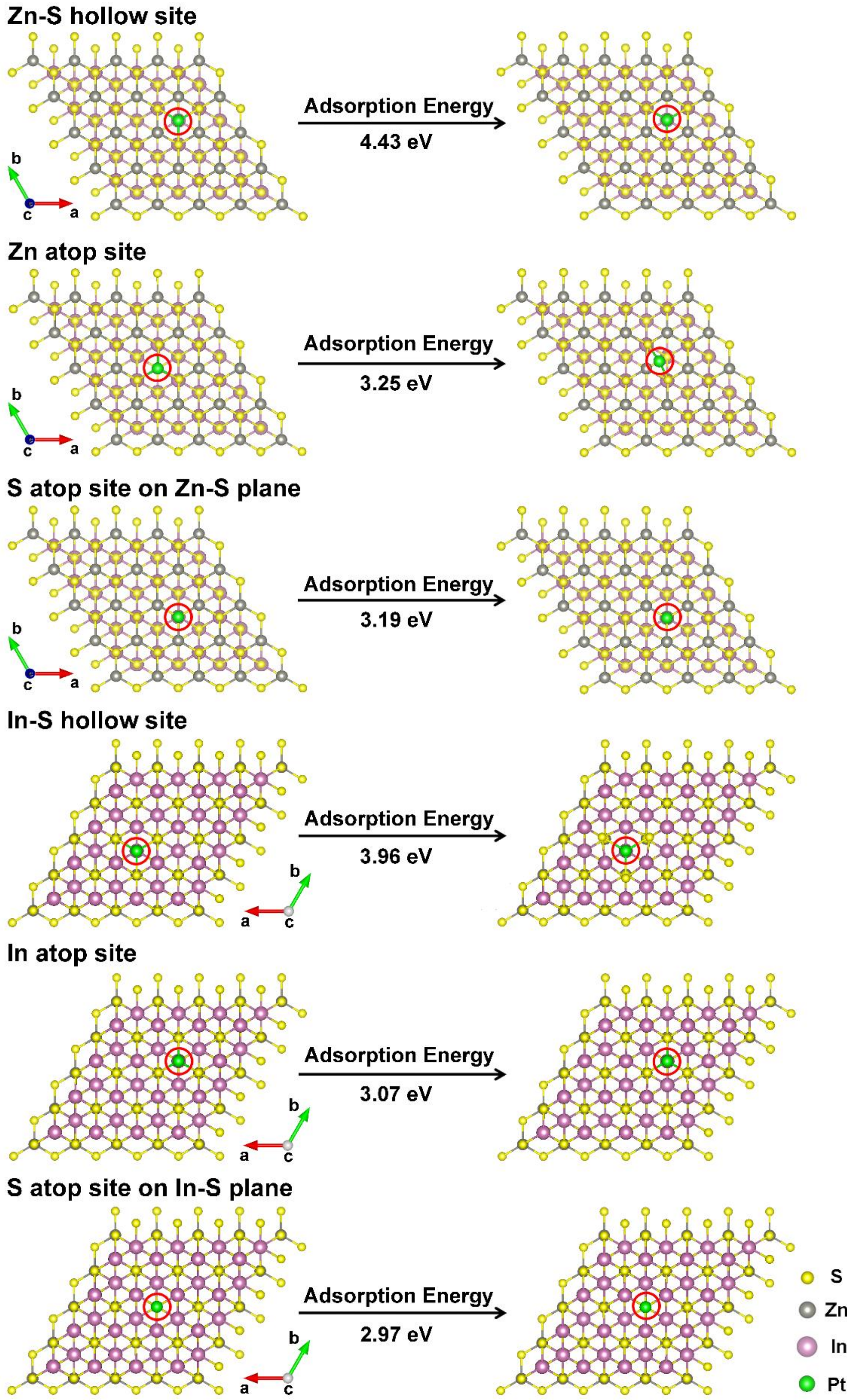


**Supplementary Figure 16.** The adsorption energy of Pt atom on Zn-S hollow site, Zn atop site, S atop site on Zn-S plane, In-S hollow site, In atop site, and S atop site on In-S plane, respectively. The Pt atom is labeled by red circles.

According to DFT calculation, Pt single atom at the Zn-S hollow site shows the adsorption energy ($E_{\mathrm{ads-Pt}}$) of 4.43 eV, which is significantly larger in comparison with the other adsorption sites. This calculation result proves that Zn-S hollow site would be the most stable location for Pt single atom occupation. This result indicates that the Zn atop position is unstable for anchoring Pt atom.

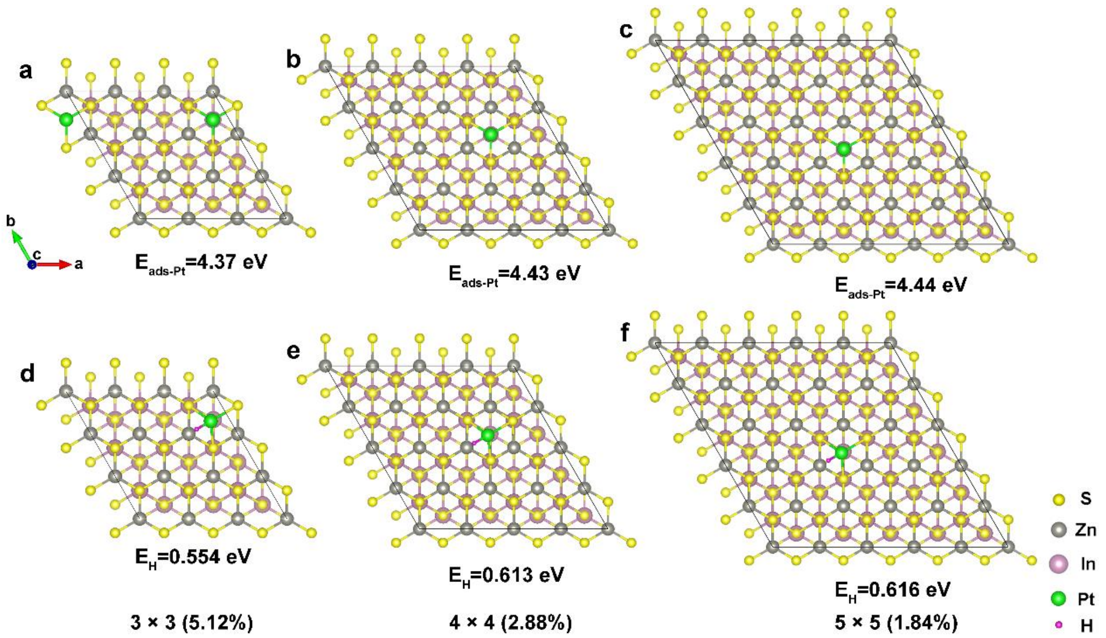


**Supplementary Figure 17.** Pt adsorption models of *h*-ZIS with different crystal slabs and Pt coverage. **a** 3×3 (5.12%), **b** 4×4 (2.88%), and **c** 5×5 (1.84%). H adsorption models of $Pt_{SS}$-ZIS with different crystal slabs. **d** 3×3, **e** 4×4, and **f** 5×5 *h*-ZIS supercell.

As shown in Supplementary Fig. 17, the adsorption energy ($E_{\mathrm{ads-Pt}}$) of Pt atom on *h*-ZIS is dependent on the Pt coverage, which gradually increases with the Pt coverage varying from 5.12% to 2.88% and finally to 1.84% (corresponding to 3×3, 4×4, and 5×5 *h*-ZIS models, respectively). The smaller difference between 5×5 and 4×4 models (0.01 eV) compared with that between 4×4 and 3×3 models (0.06 eV) demonstrate the convergence of adsorption energy of Pt single atom on *h*-ZIS. However, due to the large number of atoms in 5×5 *h*-ZIS supercell (175 atoms in total), it takes relative long time for $Pt_{SS}$-ZIS structural relaxation. More importantly, we find that the calculated adsorption energy ($\Delta E_{\mathrm{H}}$) for one H atom on Pt single site using 4×4 *h*-ZIS supercell (0.613 eV) as model is almost similar with that when 5×5 *h*-ZIS supercell (0.616 eV) is utilized, indicating that 4×4 *h*-ZIS supercell is suitable to simulate the adsorption behaviors of H atoms. Therefore, a 4×4 *h*-ZIS (involving 16 Zn atoms, 32 In atoms, and 64 S atoms) supercell is constructed as the model for the simulations.

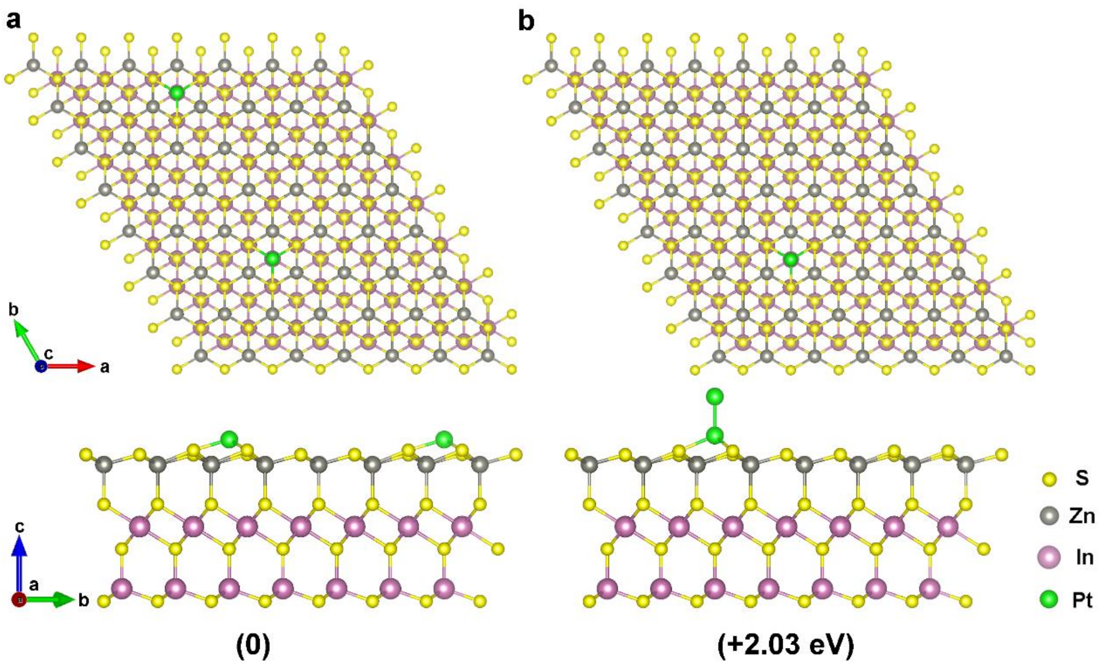


**Supplementary Figure 18.** Illustration of Pt isolated (a) and dimer (b) configurations on *h*-ZIS. The energy of the isolated configuration was taken as a zero reference for comparison.

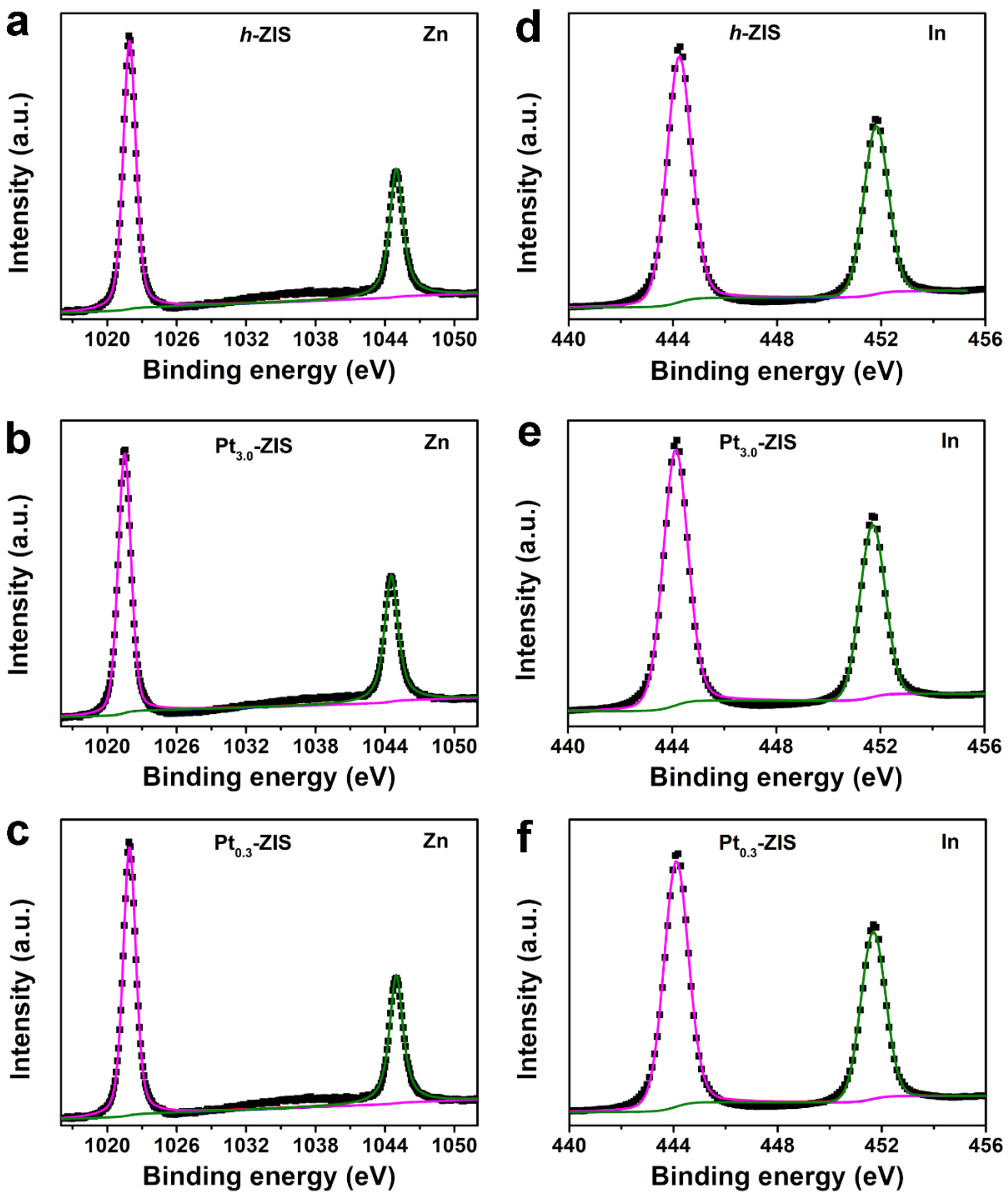


**Supplementary Figure 19.** (a-c) XPS spectra of Zn *2p* in *h*-ZIS, $Pt_{3.0}$-ZIS, and $Pt_{0.3}$-ZIS. (d-f) XPS spectra of In *3d* in *h*-ZIS, $Pt_{3.0}$-ZIS, and $Pt_{0.3}$-ZIS.

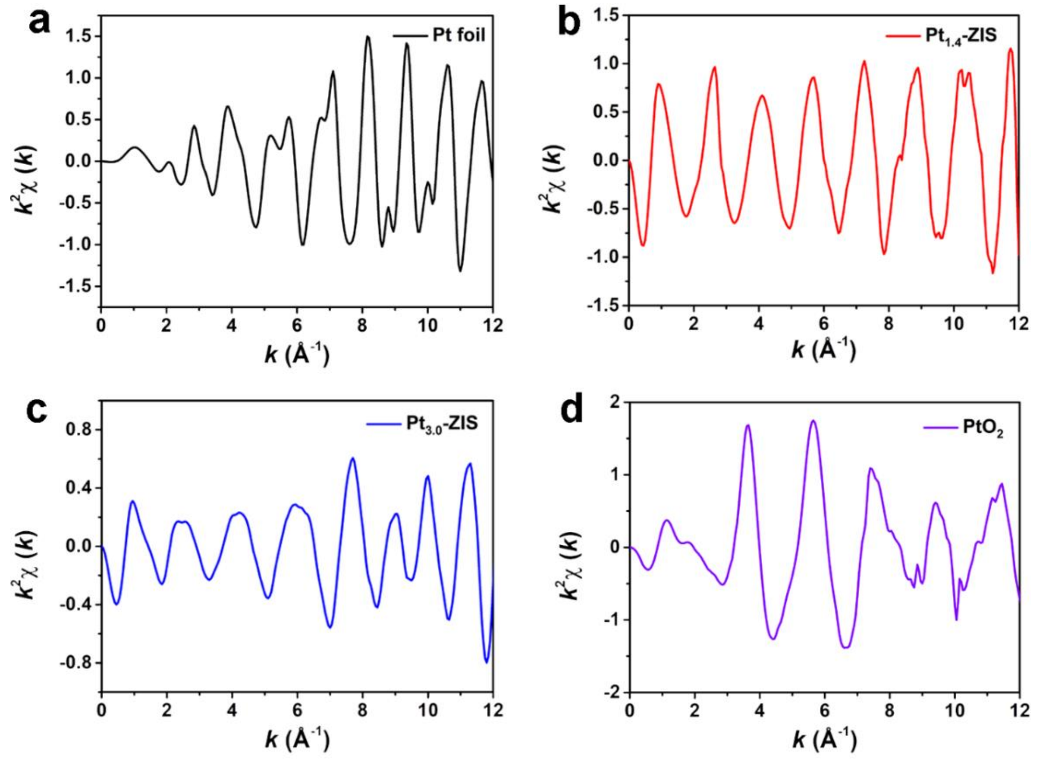


**Supplementary Figure 20.** The $k_2$-weighted EXAFS spectra in Pt k-space for Pt foil (a), $Pt_{1.4}$-ZIS (b), $Pt_{3.0}$-ZIS (c), and $PtO_2$ (d), respectively.

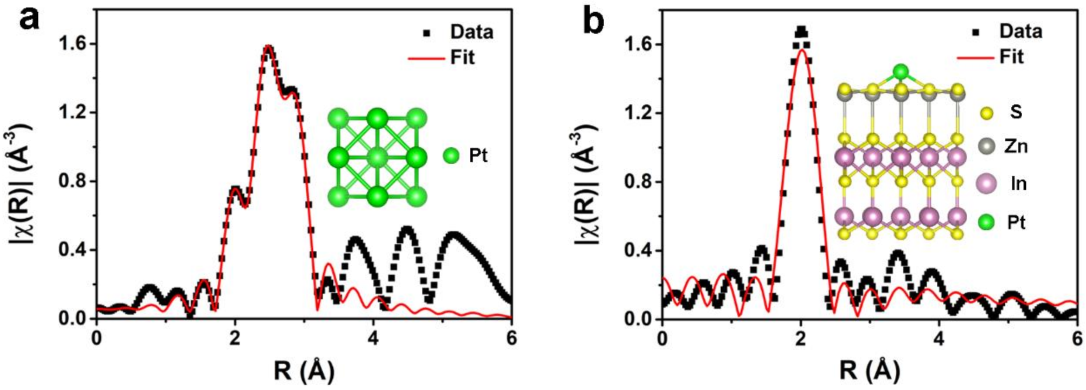


**Supplementary Figure 21.** FT-EXAFS curves between the experimental data and the fitting result of Pt foil (a), and $Pt_{1.4}$-ZIS (b). The configuration of $Pt_{1.4}$-ZIS for fitting was achieved from the DFT simulation.

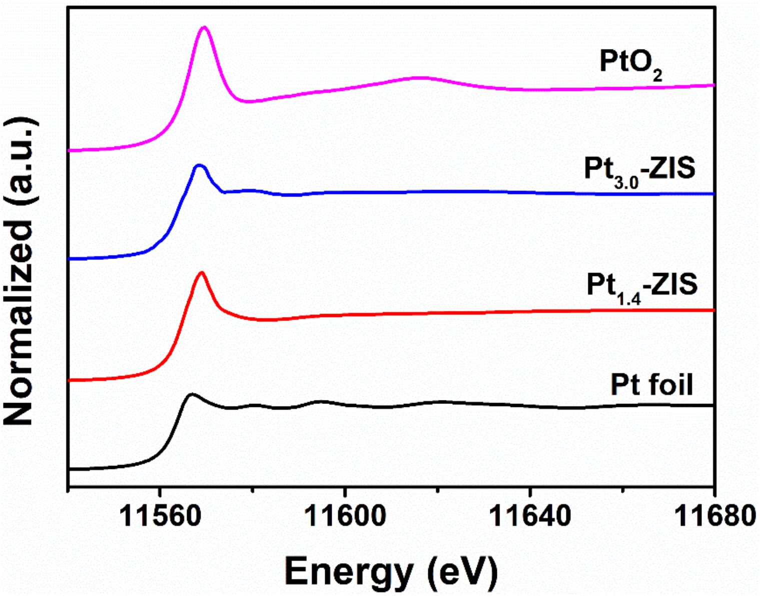


**Supplementary Figure 22.** Normalized XANES spectra at the Pt $L_3$-edge of Pt foil, $Pt_{1.4}$-ZIS, $Pt_{3.0}$-ZIS, and $PtO_2$.

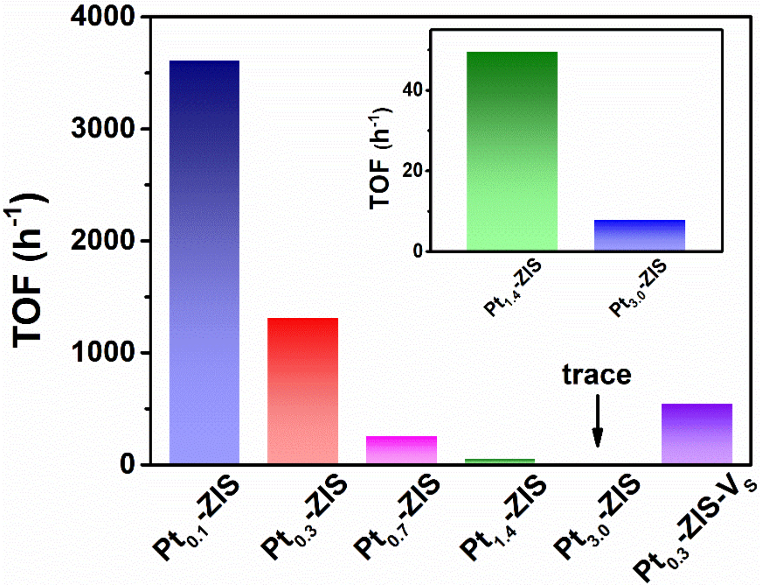


**Supplementary Figure 23.** Calculated TOFs of different Pt-ZIS photocatalysts.

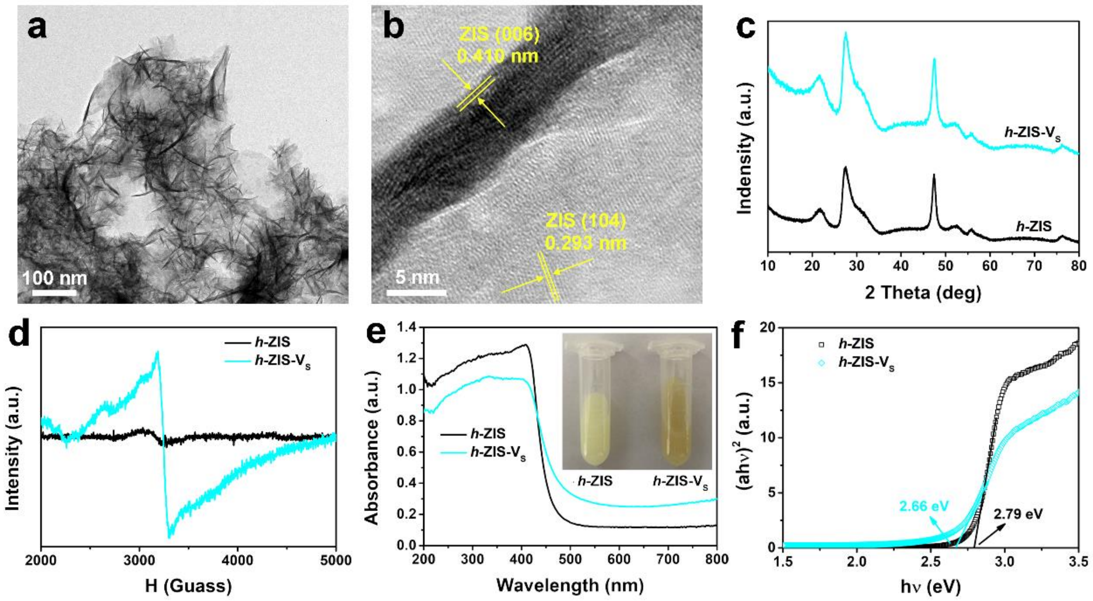


**Supplementary Figure 24.** TEM (a) and HRTEM (b) images of *h*-ZIS-VS. XRD patterns (c), ESR (d) and UV–vis diffuse reflectance spectra (e) of *h*-ZIS and *h*-ZIS-$V_S$. (f) Bandgap for *h*-ZIS and *h*-ZIS-$V_S$.

According to TEM and HRTEM images, *h*-ZIS maintains a sheet-like structure after $NaBH_4$ treatment, and the lattice fringe of 0.410 and 0.293 nm attribute to the (006) and (104) facet of *h*-ZIS, respectively. Additionally, no peak position shift could be observed in the XRD pattern, indicating that *h*-ZIS-$V_S$ keep a hexagonal crystal structure after forming sulfur vacancies. The strong ESR response of *h*-ZIS-$V_S$ compared with pristine *h*-ZIS reveals the formation of sulfur vacancies. The optical properties of *h*-ZIS-$V_S$ were examined by UV–vis diffuse reflectance spectra. Pure *h*-ZIS exhibits an absorption edge at approximately 440 nm, while the light absorption edge of *h*-ZIS-$V_S$ is redshifted and the intensity of visible light absorption is also strengthened due to the existence of $V_S$. Meanwhile, the optical bandgap of *h*-ZIS and *h*-ZIS-$V_S$ can be calculated to be 2.79 and 2.66 eV using Kubelka-Munk function. This result is consistent with the color changes.

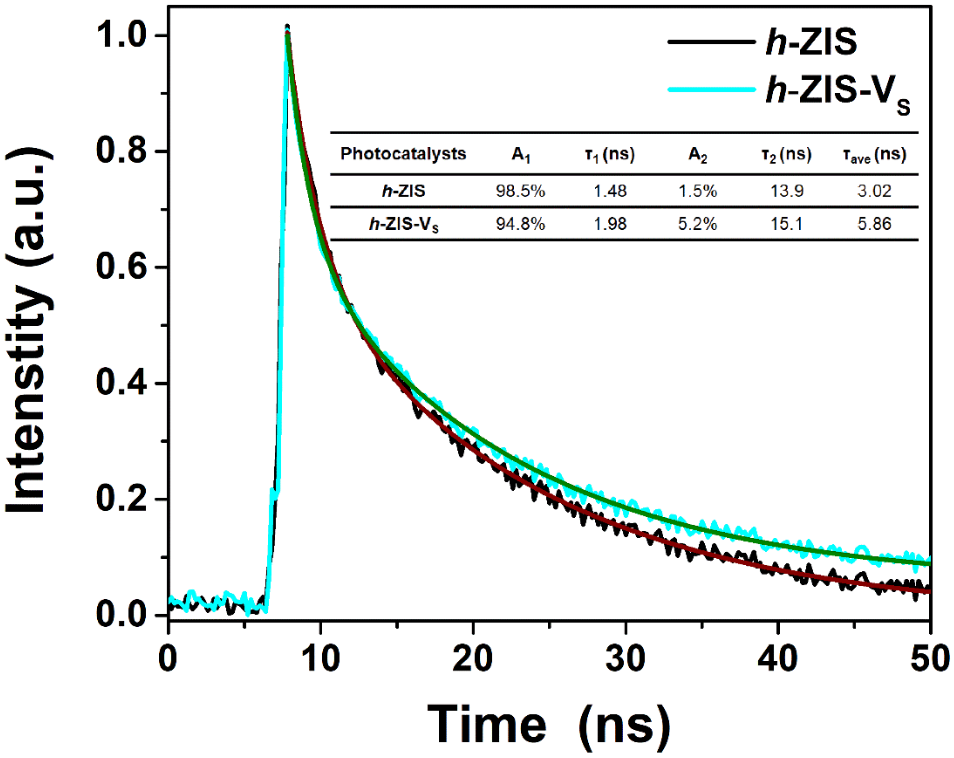


**Supplementary Figure 25.** Time-resolved PL decay of *h*-ZIS and *h*-ZIS-V$_S$. The average fluorescence lifetime is calculated according to the equation: $\tau_{ave} = \sum_{i=1}^{i=n} A_i\tau_i^2 \,/\, \sum_{i=1}^{i=n} A_i\tau_i$.

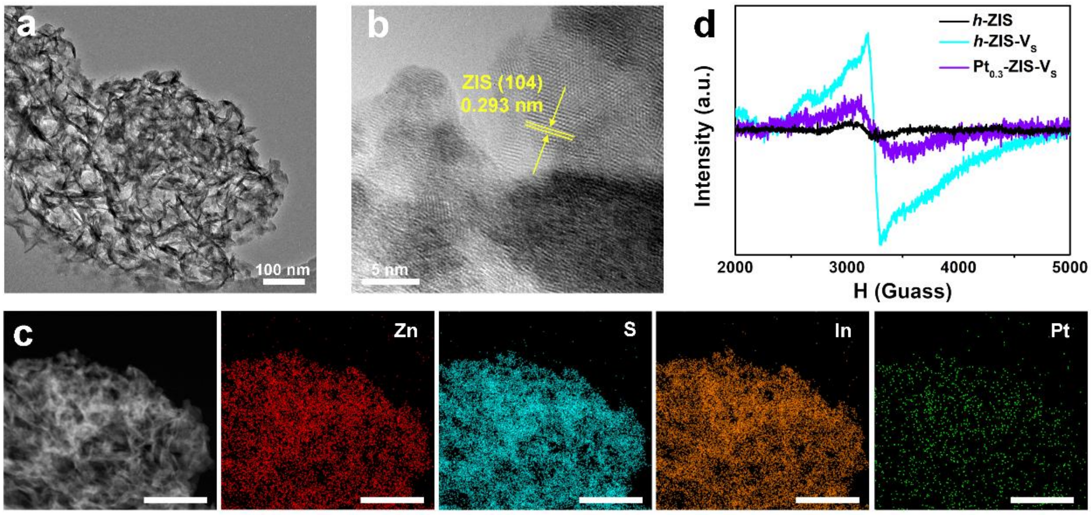


**Supplementary Figure 26.** TEM (a) and HRTEM (b) images of $Pt_{0.3}$-ZIS-$V_S$. (c) element mapping for Zn, S, In, and Pt elements of $Pt_{0.3}$-ZIS-$V_S$. The scale bar is 100 nm. (d) ESR spectra of *h*-ZIS, *h*-ZIS-$V_S$, and $Pt_{0.3}$-ZIS-$V_S$.

$Pt_{0.3}$-ZIS-$V_S$ maintains a sheet-like structure and the Pt atoms are homogeneously dispersed on the surface of *h*-ZIS-$V_S$ without any aggregation. After Pt is introduced, the ESR response is much lower than that of *h*-ZIS-$V_S$ because the vacancy is occupied by the Pt single-site.

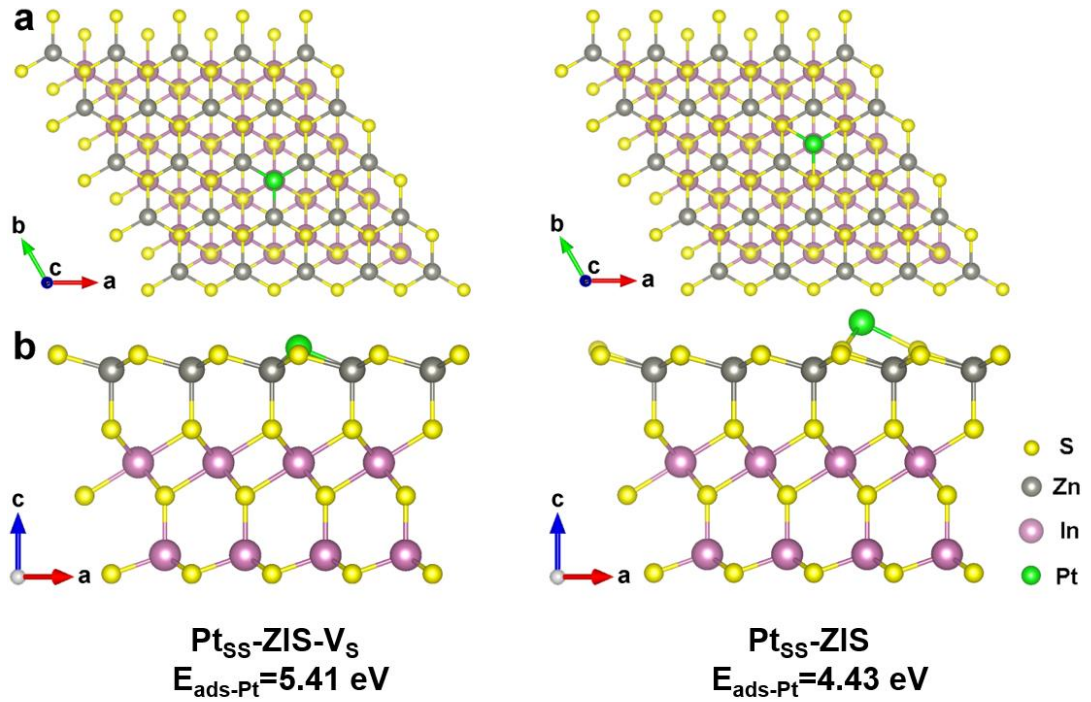


**Supplementary Figure 27.** Top view (a) and side view (b) of Pt single atom at sulfur vacancy in *h*-ZIS-$V_S$ and Zn-S hollow site in *h*-ZIS.

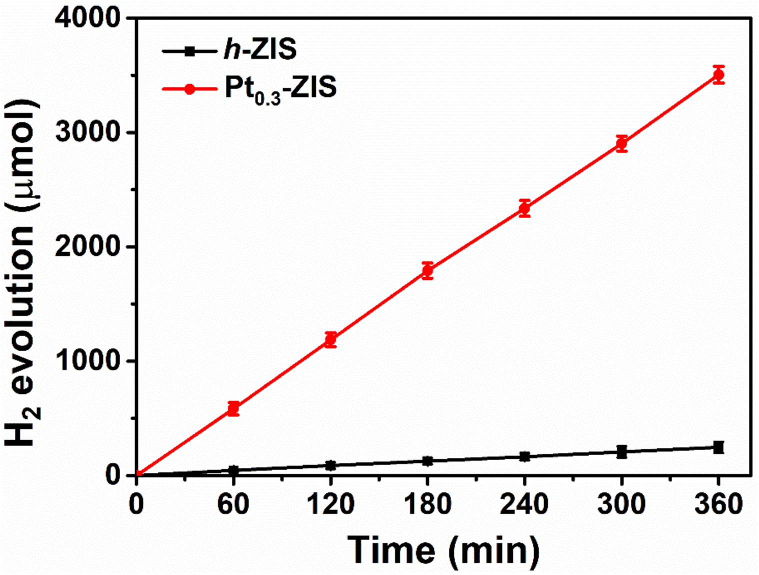


**Supplementary Figure 28.** Simulated solar light photocatalytic $H_2$ evolution activities of *h*-ZIS and $Pt_{0.3}$-ZIS, and each sample was measured three times.

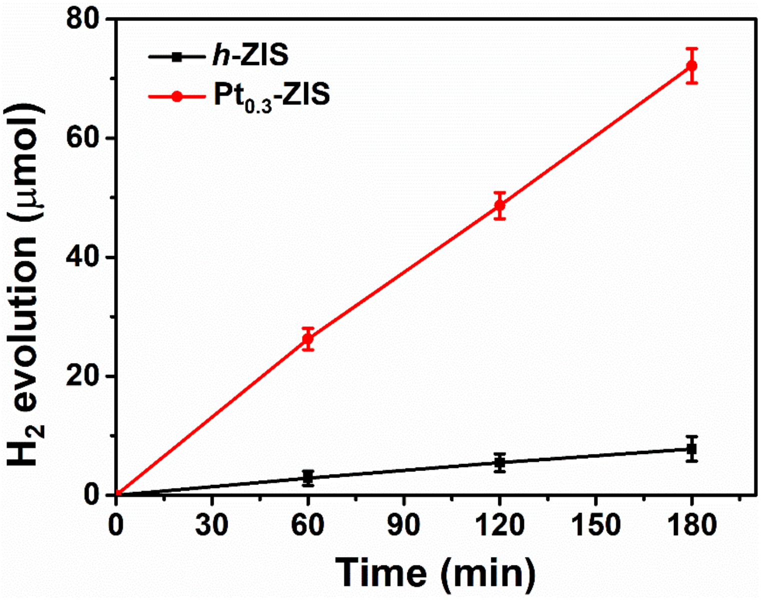


**Supplementary Figure 29.** Visible light ($\lambda$ > 420 nm) photocatalytic $H_2$ evolution activities of *h*-ZIS and $Pt_{0.3}$-ZIS in pure water, and each sample was measured three times.

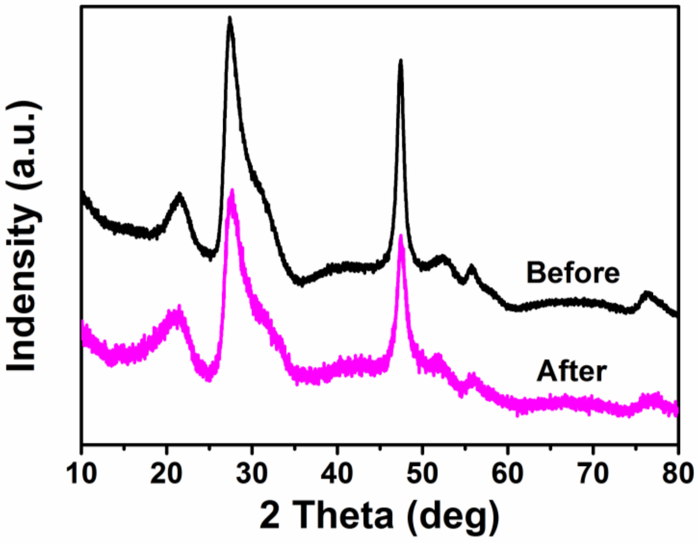


**Supplementary Figure 30.** XRD patterns of $Pt_{0.3}$-ZIS before and after long-term stability test.

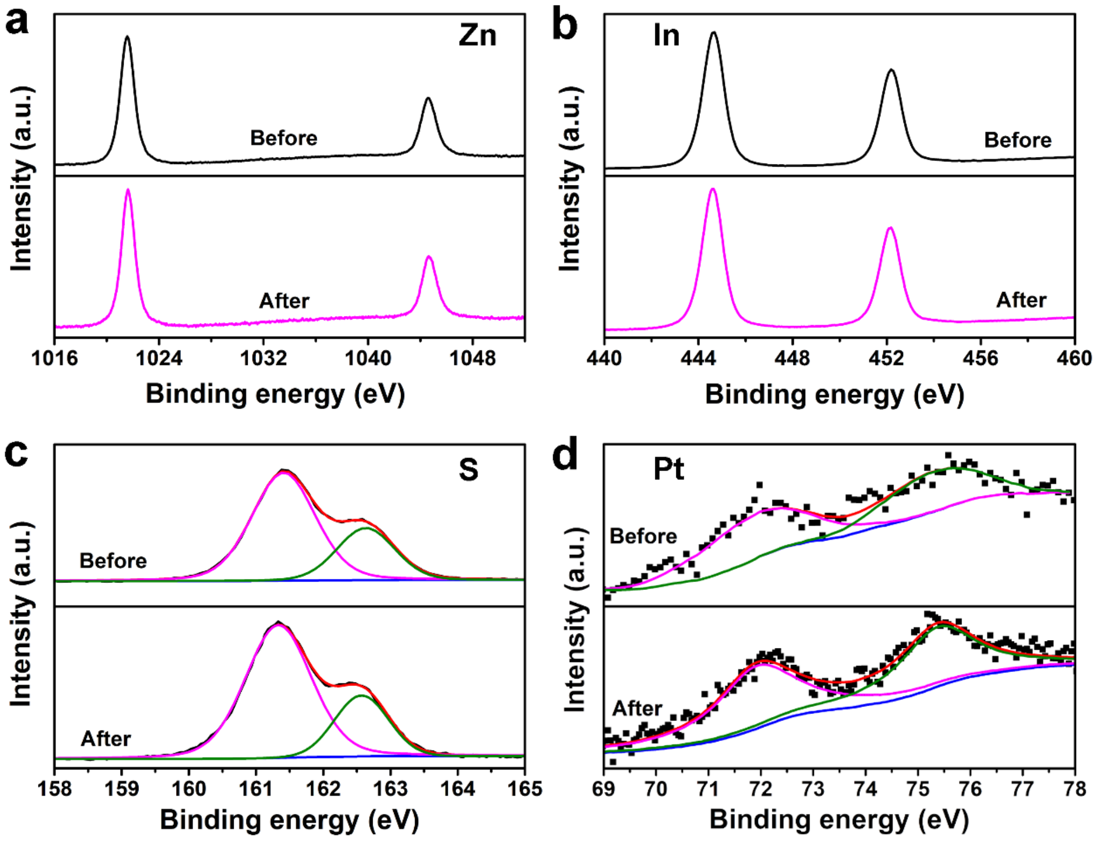


**Supplementary Figure 31.** XPS spectra of Zn *2p* (a), In *3d* (b), S *2p* (c), and Pt *4f* (d) in $Pt_{0.3}$-ZIS before and after long-term stability test.

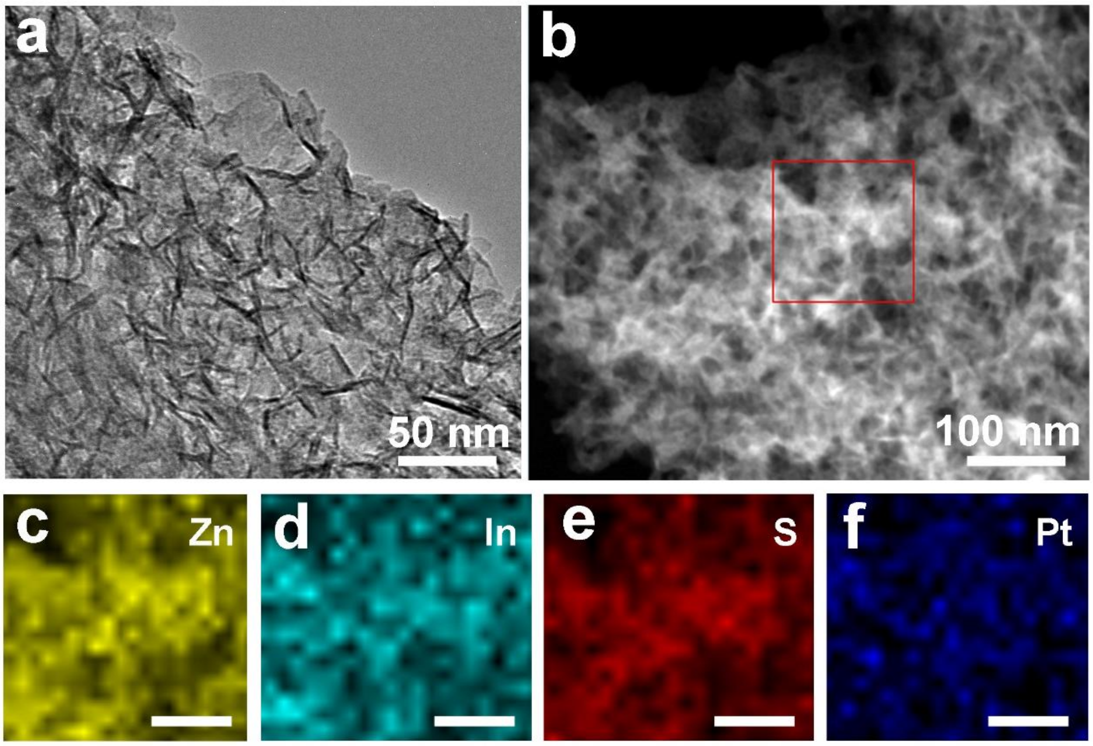


**Supplementary Figure 32.** (a, b) TEM image and (c-f) Elemental mapping for Zn, In, S, and Pt of $Pt_{0.3}$-ZIS after long-term stability test. The scale bar is 50 nm.

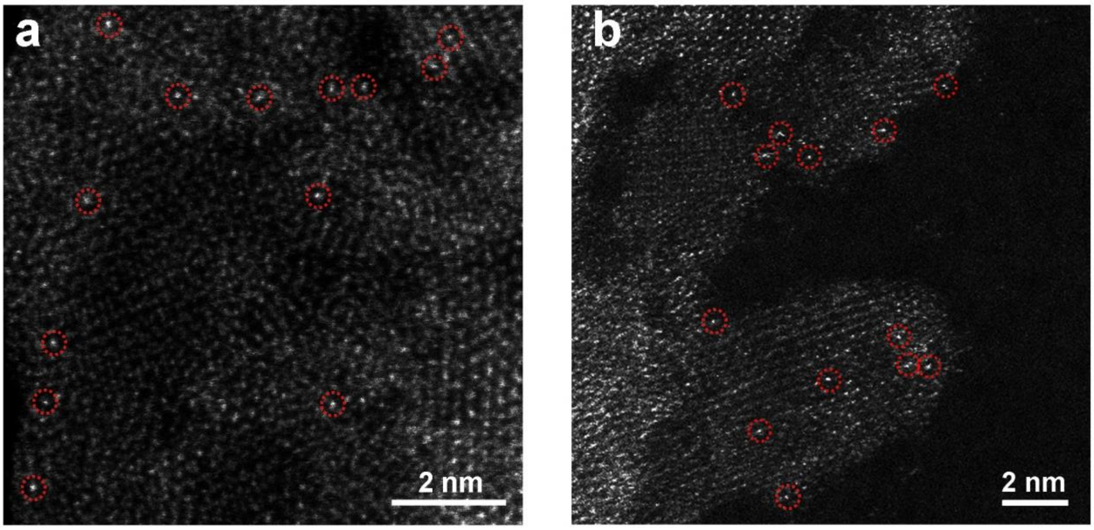


**Supplementary Figure 33.** (a, b) High resolution STEM images of $Pt_{0.3}$-ZIS after long-term stability test.

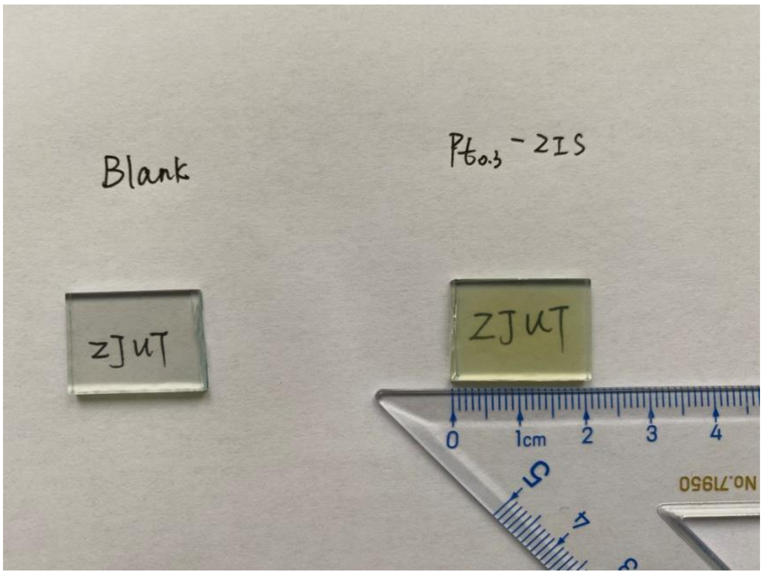


**Supplementary Figure 34.** Digital image of the $Pt_{0.3}$-ZIS thin film on glass.

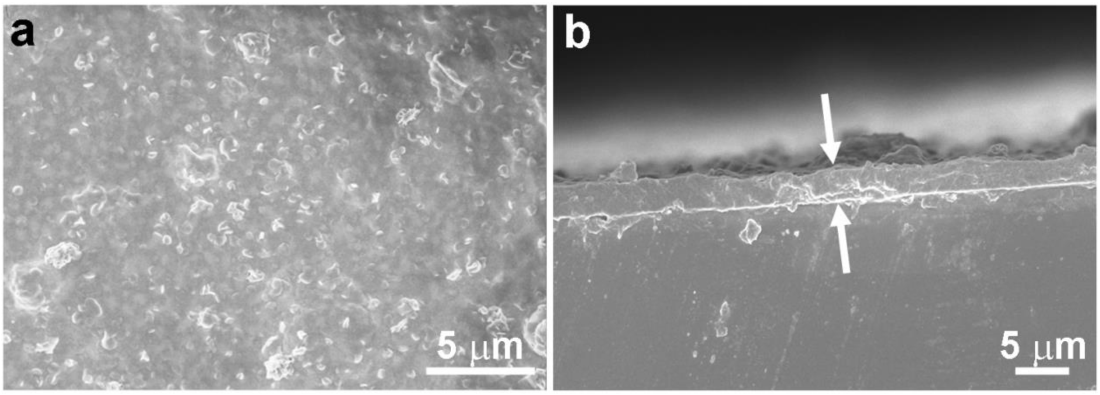


**Supplementary Figure 35.** Top-view (a) and side-view (b) SEM images of the $Pt_{0.3}$-ZIS thin film prepared by drop-casting of $Pt_{0.3}$-ZIS ethanol dispersion onto glass substrate.

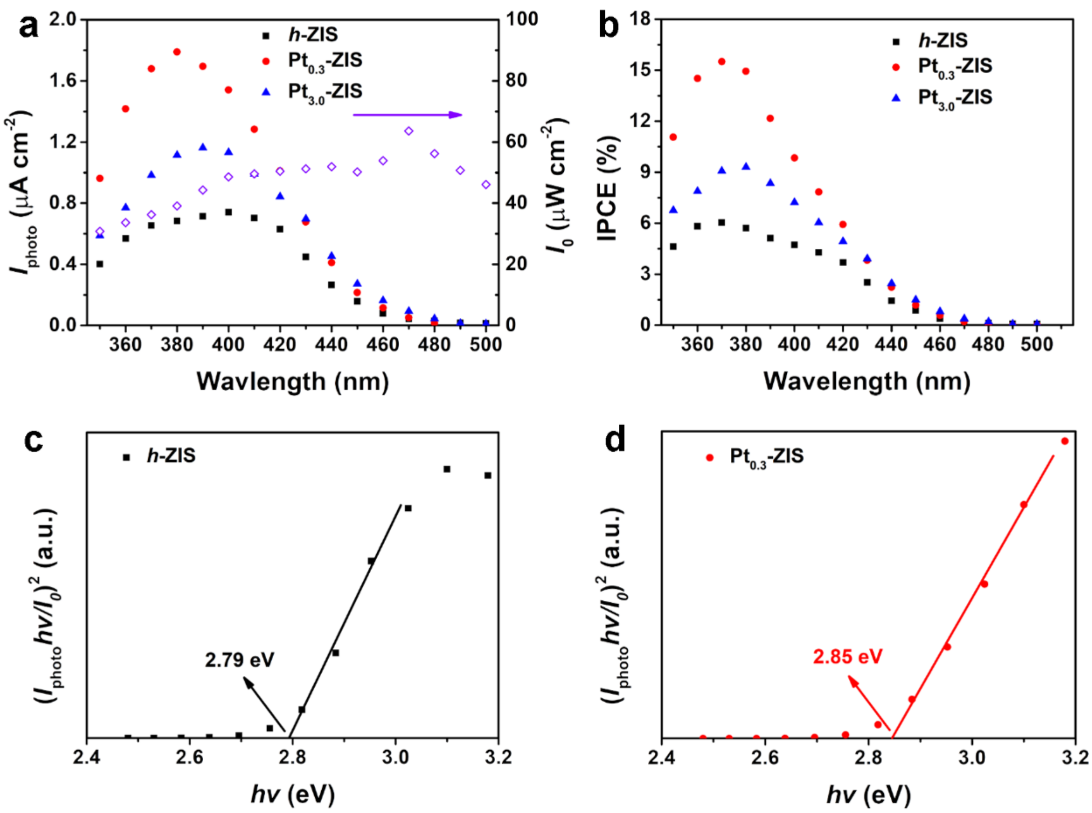


**Supplementary Figure 36.** Photocurrent action spectra (a) and IPCE (b) of *h*-ZIS, $Pt_{0.3}$-ZIS, and $Pt_{3.0}$-ZIS film electrode in the 0.1 M $Na_2SO_3$ solution as electrolyte. Bandgap for *h*-ZIS (c) and $Pt_{0.3}$-ZIS (d). The bandgap was calculated based on the equation as $(I_{\text{photo}}hv/I_0)^2 = A(hv - E_{\text{g}})$, where $I_{\text{photo}}$ is the photocurrent and $I_0$ is the intensity of incident light.

As shown in Supplementary Fig. 36, the photocurrent density of *h*-ZIS is greatly enhanced after photodepositing Pt, and $Pt_{0.3}$-ZIS exhibits the highest photocurrent and IPCE at 380 nm, demonstrating the most efficient charge separation. Additionally, the bandgap of *h*-ZIS and $Pt_{0.3}$-ZIS were also calculated, in which it increases from 2.79 eV (*h*-ZIS) to 2.85 eV ($Pt_{0.3}$-ZIS). This result is similar with the blue-shifted UV-vis absorption spectrum of $Pt_{0.3}$-ZIS.

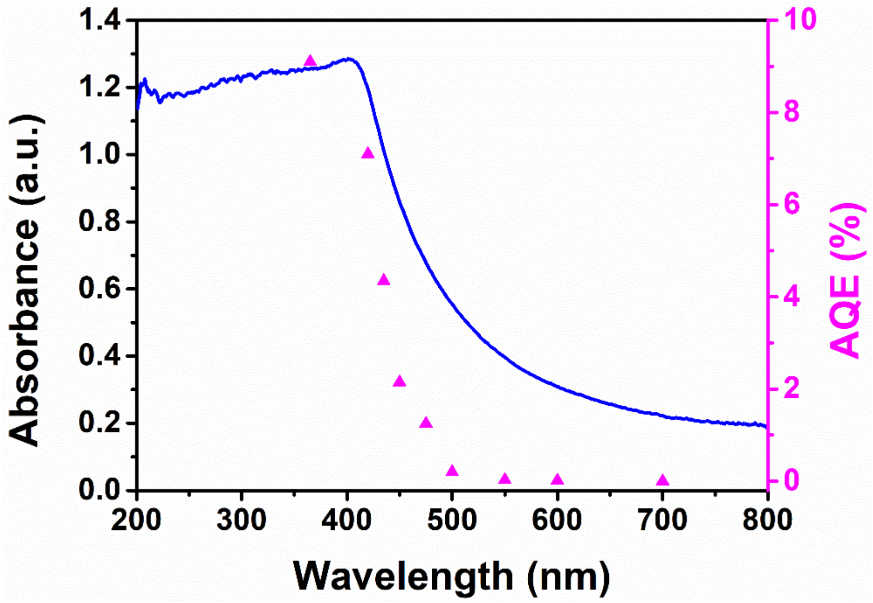


**Supplementary Figure 37.** Wavelength dependence of the AQE for $Pt_{3.0}$-ZIS.

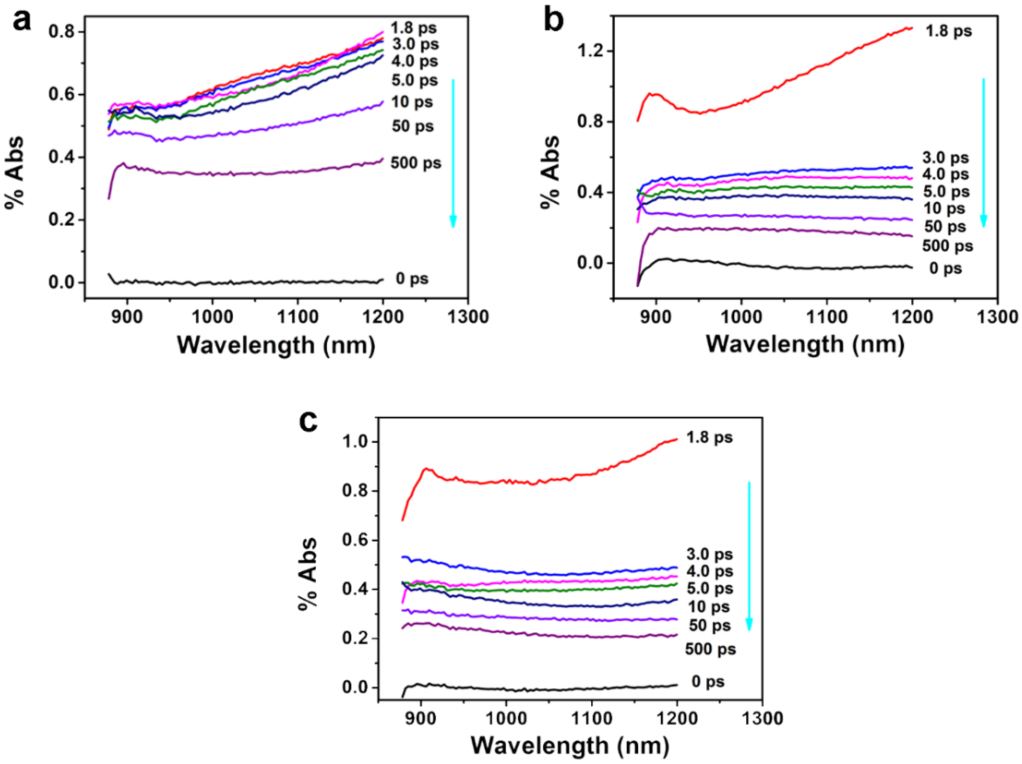


**Supplementary Figure 38.** Time-resolved diffuse reflectance (TDR) spectra observed after 420 nm laser flash photolysis of *h*-ZIS (a), $Pt_{0.3}$-ZIS (b), and $Pt_{3.0}$-ZIS (c).

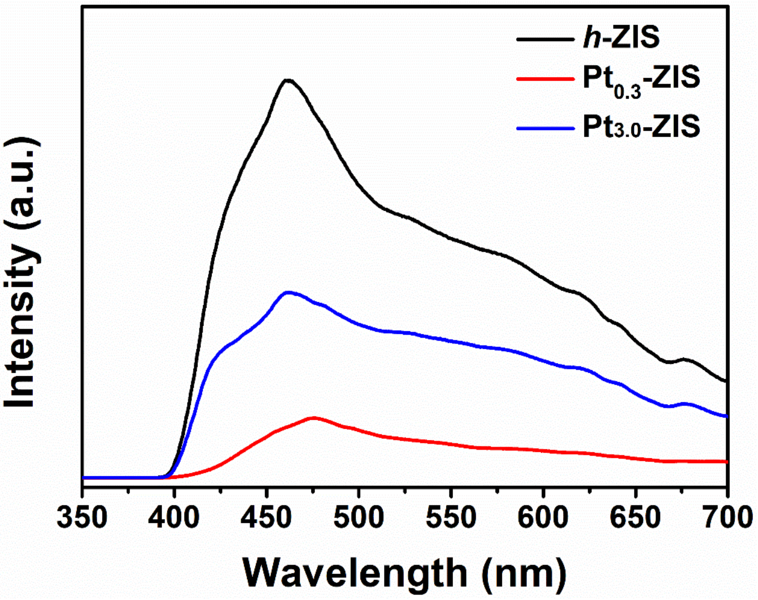


**Supplementary Figure 39.** Steady-state photoluminescence spectra of *h*-ZIS, $Pt_{0.3}$-ZIS, and $Pt_{3.0}$-ZIS.

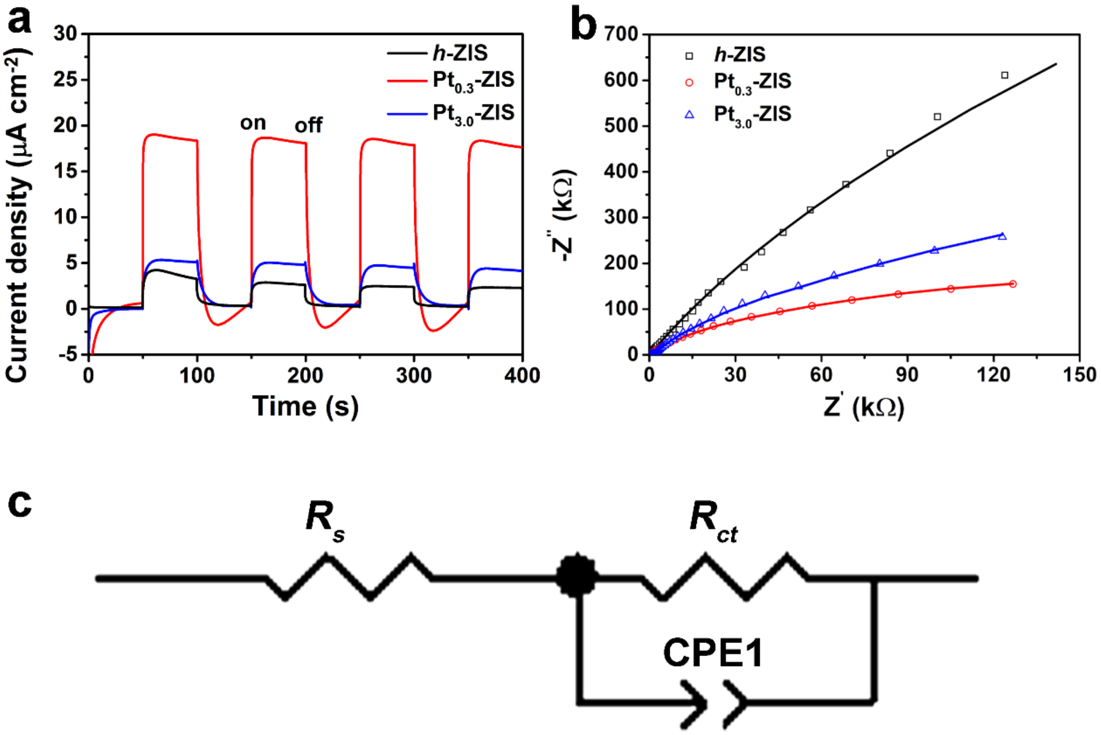


**Supplementary Figure 40.** Transient photocurrent responses of photocatalysts with visible light on/off irradiation (a), electrochemical impedance spectroscopy (b), and the corresponding equivalent circuit (c) of *h*-ZIS, $Pt_{0.3}$-ZIS, and $Pt_{3.0}$-ZIS.

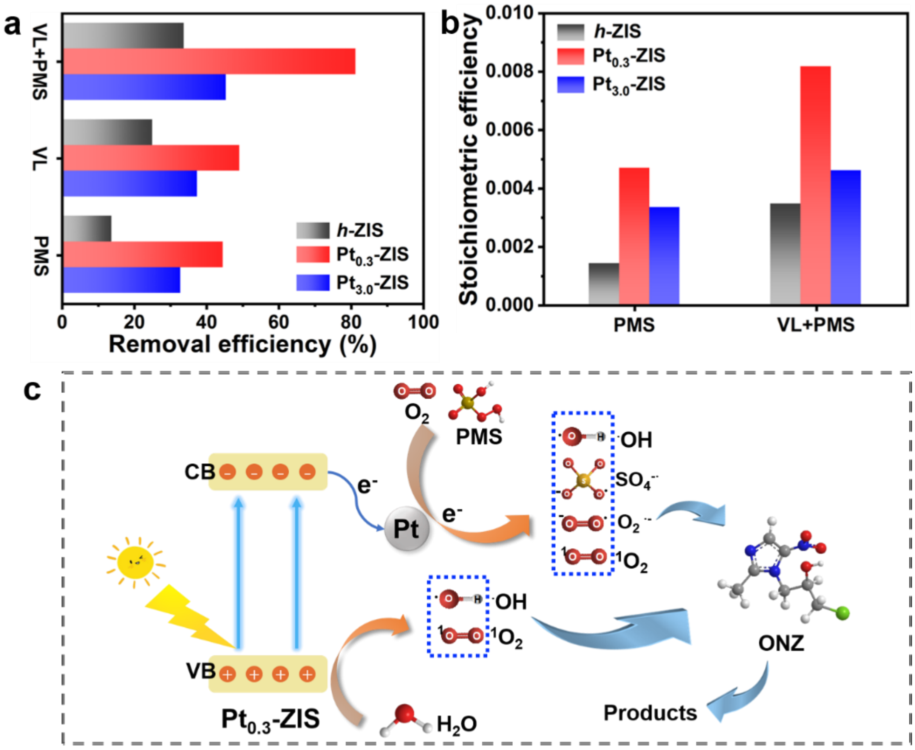


**Supplementary Figure 41.** (a) Degradation of ONZ with *h*-ZIS, $Pt_{0.3}$-ZIS, and $Pt_{3.0}$-ZIS catalysts under different system. (b) PMS stoichiometric efficiency under different system. (c) Possible degradation mechanism of ONZ in $Pt_{0.3}$-ZIS/VL/PMS system.

The efficient charge separation in $Pt_{0.3}$-ZIS was also confirmed by photocatalytic activation of peroxymonosulfate (PMS) to degrade antibiotic ornidazole (ONZ) pollutants. With PMS, the efficiencies of *h*-ZIS, $Pt_{0.3}$-ZIS, and $Pt_{3.0}$-ZIS to degrade ONZ were 13.7, 44.6 and 32.8%, respectively. Under VL irradiation, the efficiencies of *h*-ZIS, $Pt_{0.3}$-ZIS, and $Pt_{3.0}$-ZIS catalysts to degrade ONZ were 25.1, 49.2 and 37.5%, respectively. When VL and PMS were both presented, the efficiencies of *h*-ZIS, $Pt_{0.3}$-ZIS, and $Pt_{3.0}$-ZIS to degrade ONZ was greatly enhanced, especially that of $Pt_{0.3}$-ZIS to degrade ONZ increased to 81.5% within 40 min. The PMS utilization as the stoichiometric efficiency was calculated by correlating the PMS decomposition and ONZ degradation performance. In the absence of VL, the stoichiometric efficiencies of PMS for *h*-ZIS, $Pt_{0.3}$-ZIS, and $Pt_{3.0}$-ZIS are 0.0014, 0.0049 and 0.0033, respectively. Under VL irradiation, the utilization efficiencies of PMS are all improved, especially that of $Pt_{0.3}$-ZIS achieved the highest efficiency (0.0082).

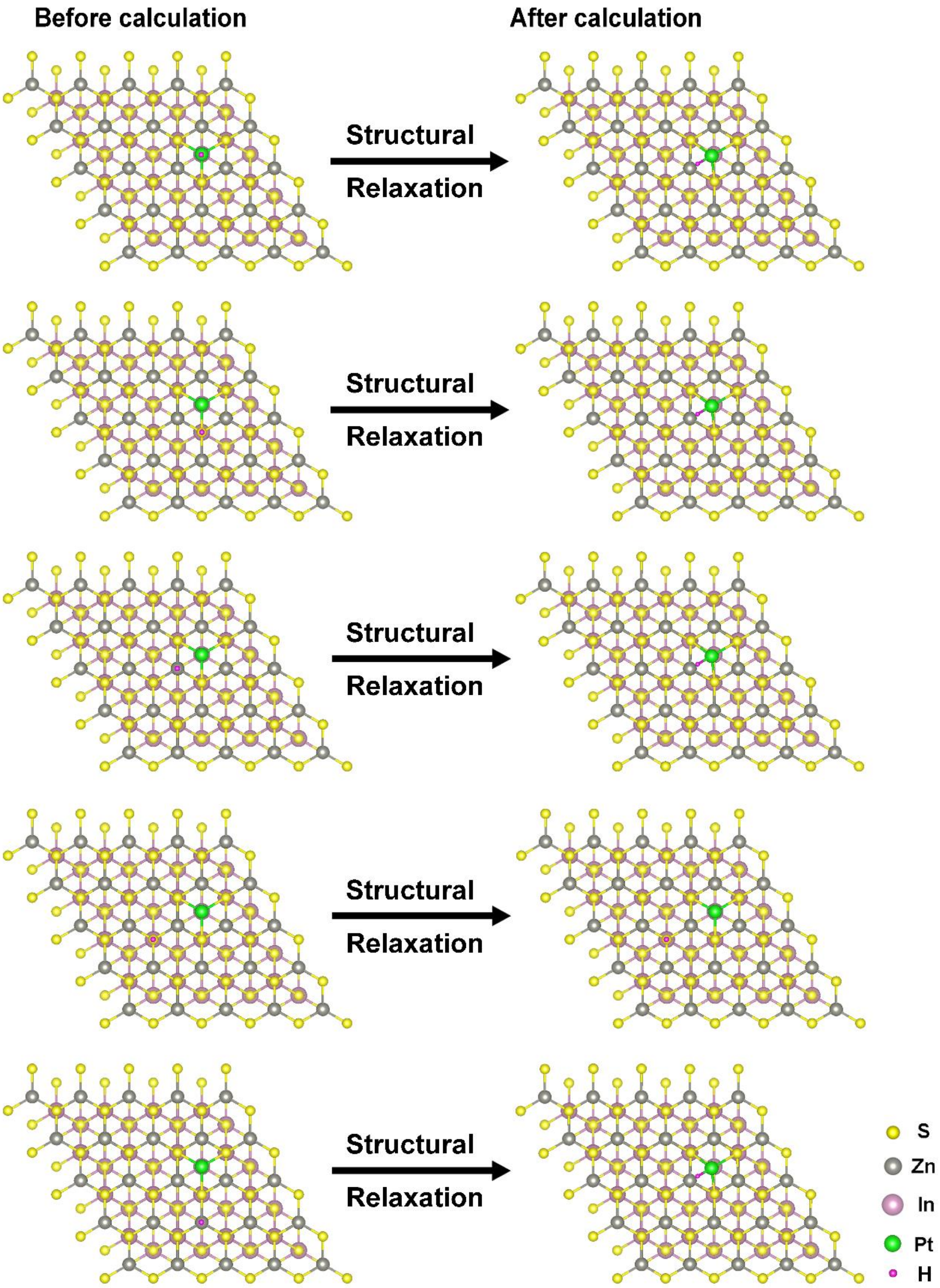


**Supplementary Figure 42.** Top view of H atom on Pt site, neighboring S site, neighboring Zn site, second-neighboring S site, and second-neighboring Zn site before and after structural relaxation.

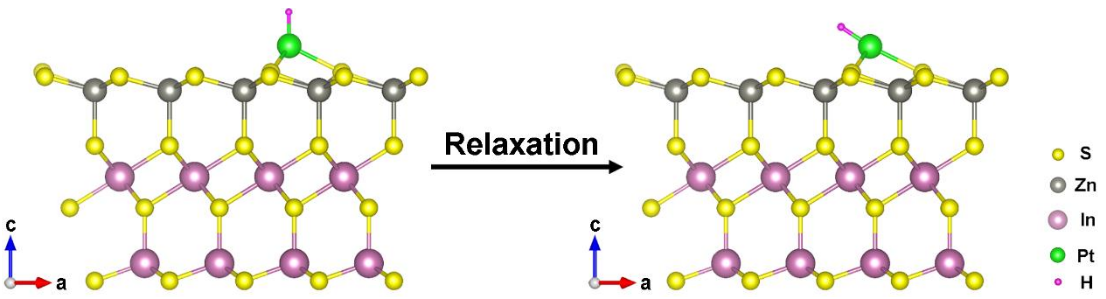


**Supplementary Figure 43.** Side view of H atom on Pt site before and after relaxation.

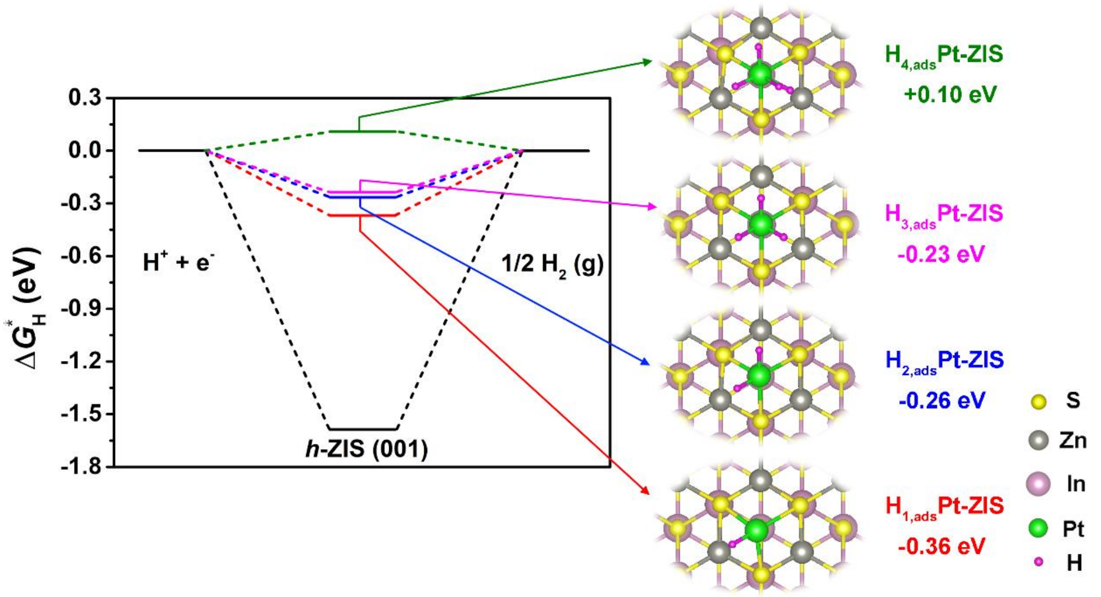


**Supplementary Figure 44.** Calculated free-energy diagram of the HER at the equilibrium potential and assuming that Pt is the active site. The inset shows the model of H adsorbed on the Pt site.

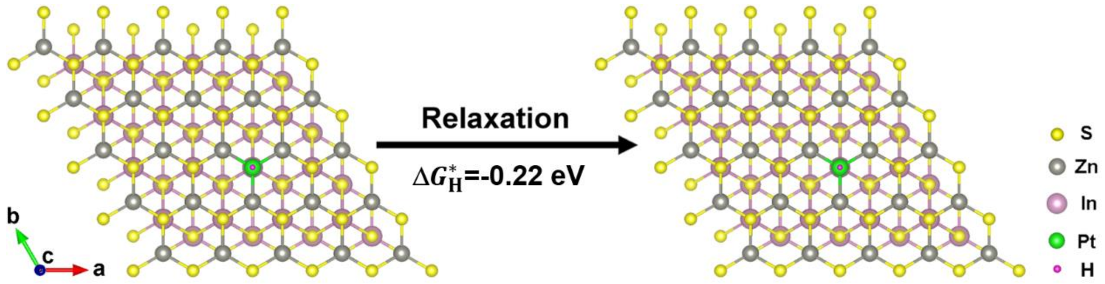


**Supplementary Figure 45.** Top view of H atom on Pt site before and after structural relaxation for $Pt_{SS}$-ZIS-$V_S$.

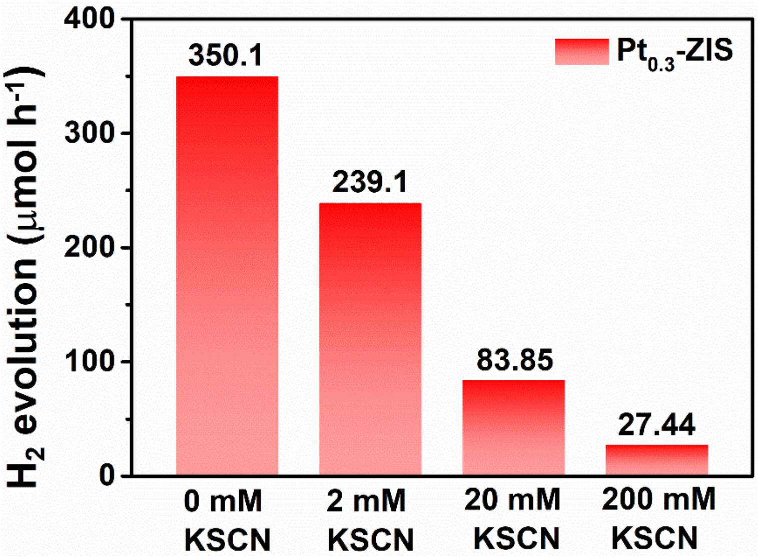


**Supplementary Figure 46.** Photocatalytic $H_2$ evolution of $Pt_{0.3}$-ZIS without and with KSCN in different concentrations.

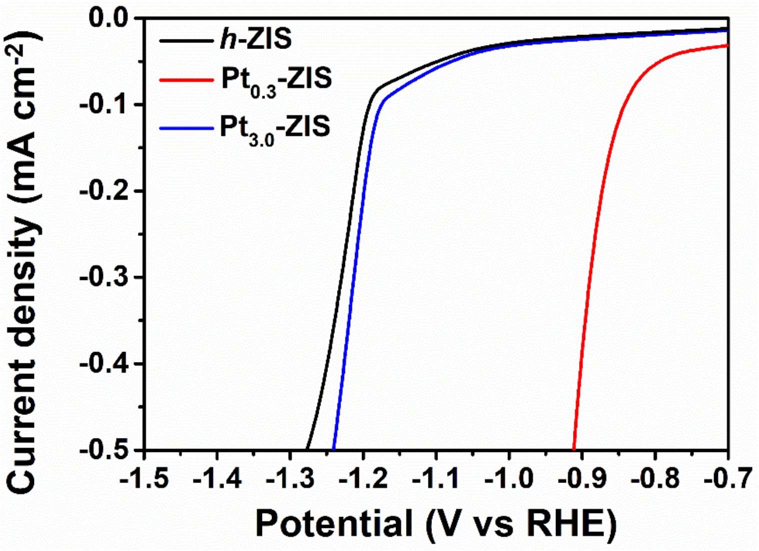


**Supplementary Figure 47.** *J-V* curves of *h*-ZIS, $Pt_{0.3}$-ZIS, and $Pt_{3.0}$-ZIS in 0.5 M NaOH aqueous solution without light irradiation.

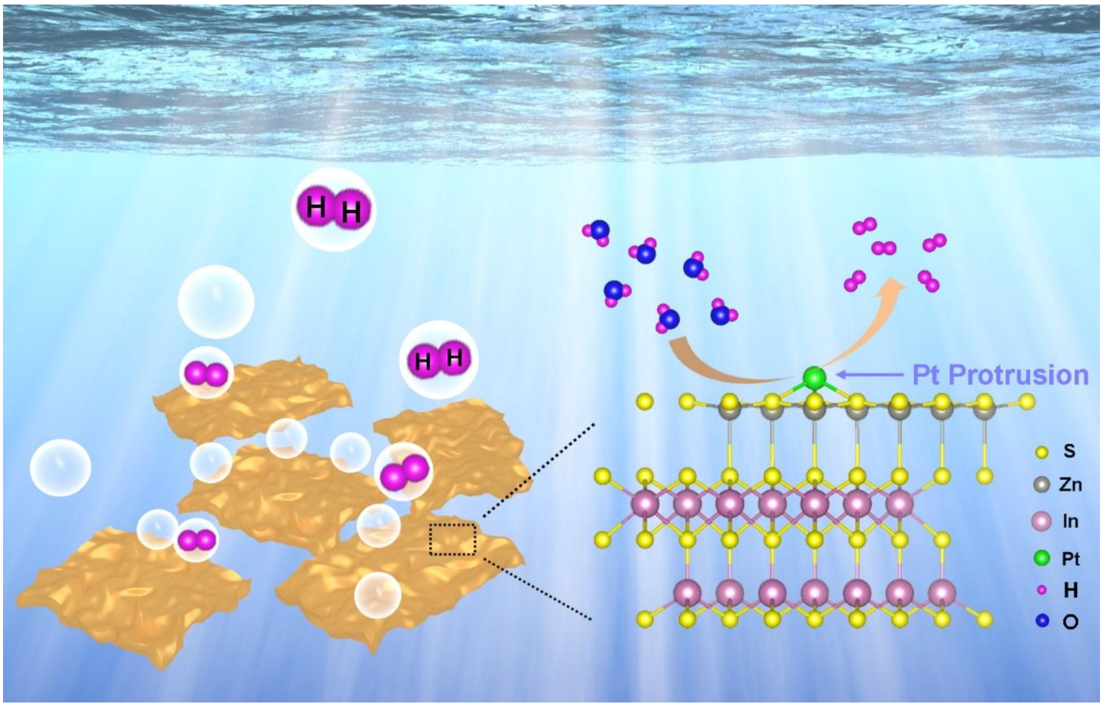


**Supplementary Figure 48.** Scheme diagram of Photocatalytic HER processes for $Pt_{SS}$-ZIS.

## 3. Supplementary Tables

**Supplementary Table 1.** The different contents of metal atoms in *h*-ZIS and Pt-ZIS measured by ICP-OES and their theoretical calculations.

| **Sample** | **$H_2PtCl_6 \cdot H_2O$ (mL)** | **Pt (wt%) ICP-OES** | **Pt (wt%) theoretical** |
|---|---|---|---|
| *h*-ZIS | 0 | 0 | 0 |
| $Pt_{0.1}$-ZIS | 0.0133 | 0.08 | 0.10 |
| $Pt_{0.3}$-ZIS | 0.0398 | 0.26 | 0.30 |
| $Pt_{0.7}$-ZIS | 0.0930 | 0.68 | 0.70 |
| $Pt_{1.4}$ -ZIS | 0.186 | 1.33 | 1.40 |
| $Pt_{3.0}$-ZIS | 0.398 | 2.88 | 3.00 |
| $Pt_{0.3}$-ZIS-$V_S$ | 0.0398 | 0.28 | 0.30 |

**Supplementary Table 2.** Pt *4f* XPS fitting data of Pt/C, $Pt_{3.0}$-ZIS, and $Pt_{0.3}$-ZIS.

| **Catalysts** | **Pt $4f_{7/2}$** | | | **Pt $4f_{5/2}$** | | |
|---|---|---|---|---|---|---|
| | **Peak (eV)** | **FWHM** | **Area** | **Peak (eV)** | **FWHM** | **Area** |
| **Pt/C** | 70.90, 71.77, 73.19 | 1.42, 1.52, 2.65 | 11786, 9124, 6078 | 74.3, 74.9, 76.3 | 1.53, 1.80, 2.70 | 8839, 7332, 5812 |
| **$Pt_{3.0}$-ZIS** | 70.90, 72.09 | 1.39, 1.12 | 1677, 4552 | 74.4, 75.4 | 1.50, 1.07 | 1270, 3389 |
| **$Pt_{0.3}$-ZIS** | 72.10 | 2.33 | 953 | 75.4 | 2.23 | 715 |

**Supplementary Table 3.** S *2p* XPS fitting data of *h*-ZIS, $Pt_{3.0}$-ZIS, and $Pt_{0.3}$-ZIS.

| **Catalysts** | **S $2p_{3/2}$** | | | **S $2p_{1/2}$** | | |
|---|---|---|---|---|---|---|
| | **Peak (eV)** | **FWHM** | **Area** | **Peak (eV)** | **FWHM** | **Area** |
| ***h*-ZIS** | 161.8 | 1.10 | 34802 | 163.0 | 1.05 | 15888 |
| **$Pt_{3.0}$-ZIS** | 161.5 | 1.09 | 45300 | 162.7 | 1.03 | 18828 |
| **$Pt_{0.3}$-ZIS** | 161.4 | 1.11 | 38818 | 162.6 | 1.04 | 16251 |

**Supplementary Table 4.** Zn *2p* XPS fitting data of *h*-ZIS, $Pt_{3.0}$-ZIS, and $Pt_{0.3}$-ZIS.

| Catalysts | Zn $2p_{3/2}$ | | | Zn $2p_{1/2}$ | | |
|---|---|---|---|---|---|---|
| | **Peak (eV)** | **FWHM** | **Area** | **Peak (eV)** | **FWHM** | **Area** |
| ***h*-ZIS** | 1021.9 | 1.29 | 92932 | 1045.0 | 1.38 | 55185 |
| **$Pt_{3.0}$-ZIS** | 1021.5 | 1.31 | 110741 | 1044.6 | 1.39 | 68412 |
| **$Pt_{0.3}$-ZIS** | 1031.7 | 1.31 | 97986 | 1044.8 | 1.39 | 61868 |

**Supplementary Table 5.** In *3d* XPS fitting data of *h*-ZIS, $Pt_{3.0}$-ZIS, and $Pt_{0.3}$-ZIS.

| Catalysts | In $3d_{5/2}$ | | | Zn $3d_{3/2}$ | | |
|---|---|---|---|---|---|---|
| | **Peak (eV)** | **FWHM** | **Area** | **Peak (eV)** | **FWHM** | **Area** |
| ***h*-ZIS** | 444.2 | 1.11 | 216668 | 541.8 | 1.11 | 141209 |
| **$Pt_{3.0}$-ZIS** | 444.1 | 1.12 | 270440 | 451.7 | 1.14 | 170485 |
| **$Pt_{0.3}$-ZIS** | 444.1 | 1.11 | 232328 | 451.7 | 1.11 | 148713 |

**Supplementary Table 6.** Fitting results of *R* space of Pt foil and $Pt_{1.4}$-ZIS.

| Sample | Shell | N | R (Å) | $\sigma^2$ ($10^{-3}$ Å$^2$) | $\Delta E_0$ (eV) | R-factor |
|---|---|---|---|---|---|---|
| **Pt foil** | Pt-Pt | 12 (fixed) | 2.762 ± 0.002 | 4.4 ± 0.3 | 7.51 ± 0.39 | 0.003 |
| **$Pt_{1.4}$-ZIS** | Pt-S | 2.6 ± 0.2 | 2.456 ± 0.057 | 6.2 ± 1.2 | 11.90 ± 4.26 | 0.024 |

**Supplementary Table 7.** $H_2$ evolution at each wavelength of $Pt_{0.3}$-ZIS and $Pt_{3.0}$-ZIS for 1 h.

| Wavelength (nm) | $H_2$ evolution (μmol) | | Light intensity (mW cm$^{-2}$) | AQE (%) | |
|---|---|---|---|---|---|
| | **$Pt_{0.3}$-ZIS** | **$Pt_{3.0}$-ZIS** | | **$Pt_{0.3}$-ZIS** | **$Pt_{3.0}$-ZIS** |
| 365 | 44.84 | 6.48 | 0.4588 | 63.0 | 9.1 |
| 420 | 116.2 | 16.37 | 1.292 | 50.4 | 7.1 |
| 435 | 74.66 | 11.33 | 1.409 | 28.67 | 4.35 |
| 450 | 51.27 | 11.92 | 2.898 | 9.25 | 2.15 |
| 475 | 15.21 | 5.98 | 6.332 | 1.19 | 1.25 |
| 500 | 2.61 | 3.48 | 8.188 | 0.15 | 0.26 |
| 550 | 0.81 | 1.16 | 16.50 | 0.021 | 0.030 |
| 600 | 0.68 | 0.82 | 17.78 | 0.015 | 0.018 |
| 700 | 0.00 | 0.00 | 21.0 | 0.00 | 0.00 |

**Supplementary Table 8.** Comparison of representative ZIS and $Pt_{SS}$-based photocatalysts and their $H_2$ evolution behaviors.

| **Catalysts** | **Condition** | **$H_2$ (mmol $g^{-1}$ $h^{-1}$)** | **Mass Activity (mmol $mg_{Pt}^{-1}$ $h^{-1}$)** | **AQE** |
|---|---|---|---|---|
| $Pt_{0.3}$-ZIS (0.3 wt%) (this work) | TEOA (10 vol%) $\lambda > 420$ nm | 17.50 | 5.834 | 50.4% (420 nm) |
| $Pt_{0.3}$-ZIS (0.3 wt%) (this work) | TEOA (10 vol%) Simulated solar light | 29.20 | 9.733 | 50.4% (420 nm) |
| PtSA/$Cs_2SnI_6$[7] (0.12 wt%) | HI solution containing 20 vol% $H_3PO_2$ $\lambda \geq 420$ nm | 0.430 | 0.3583 | - |
| Ni SA-NG/$SrTiO_3$(Al)/$CoO_x$[8] | Pure water 280 W Xenon lamp full arc | 0.498 | - | - |
| Ni-a/$TiO_2$[9] | Methanol (10 vol%) Full spectrum irradiation | 1.890 | - | - |
| O-doped ZIS[10] | $Na_2S/Na_2SO_3$ $\lambda > 420$ nm | 2.120 | - | - |
| RGO/ZIS[11] | TEOA (10 vol%) $\lambda > 420$ nm | 2.641 | - | 4.4% (420 nm) |
| $MoS_2$/CQDs/ZIS[12] | TEOA (10 vol%) $\lambda > 420$ nm | 2.696 | - | 25.6% (420 nm) |
| ZIS/g-$C_3N_4$[13] | TEOA (10 vol%) $\lambda > 420$ nm | 2.780 | - | 3.8% (420 nm) |
| PtSA-CN620[14] (1.72 wt%) | TEOA (10 vol%) $\lambda > 400$ nm | 3.020 | 0.1756 | 0.544% (420 nm) |
| CNFs@$ZnIn_2S_4$[15] | $Na_2S/Na_2SO_3$ $\lambda > 420$ nm | 3.167 | - | 25.35% (420 nm) |
| J-$ZnIn_2S_4$[16] | $Na_2S/Na_2SO_3$ $\lambda > 420$ nm | 3.807 | - | 18.67% (420 nm) |
| $Mo_{10}G_1/ZnIn_2S_4$[17] | $Na_2S/Na_2SO_3$ $\lambda > 420$ nm | 4.167 | - | - |

| | | | | |
|---|---|---|---|---|
| $Ni_{0.7}$-ZIS[4] | TEOA (10 vol%) λ > 420 nm | 4.215 | - | 17.1% (420 nm) |
| $Co_9S_8$@ $ZnIn_2S_4$[18] | TEOA (20 vol%) λ > 400 nm | 6.25 | - | - |
| Pt-CN[19] (0.16 wt%) | TEOA (10 vol%) 300 W Xe lamp | 6.36 | 3.975 | - |
| $MoS_2$ QDs@Vs-M-ZIS[20] | Lactic acid (10 vol%) Simulated solar light | 6.884 | - | 63.87% (420 nm) |
| $Pt_1/TiO_2$-A[21] (0.6 wt%) | Methanol (20 vol%) 300 W Xenon lamp | 8.45 | 1.408 | - |
| Pt-CdS-N[22] (0.014 wt%) | Lactic acid (10 vol%) λ > 400 nm | 10.29 | 73.50 | - |
| SA-Cu-TCN[23] (1.0 wt%) | Methanol (15 vol%) λ > 420 nm | 10.60 | 1.060 | 9.2% (420 nm) |
| Co/NGC@ZIS[24] | TEOA (17 vol%) λ > 400 nm | 11.27 | - | 5.07% (420 nm) |
| PtSA-MNSs[25] (12.0 wt%) | Ascorbic acid (0.1 M) λ > 420 nm | 11.32 | 0.094 | - |
| M-ZIS-S[26] | TEOA (10 vol%) λ > 420 nm | 13.48 | - | 53.68% (365 nm) |
| ML-$MoS_2/MAPbI_3$-MCs[27] | $HI/H_3PO_2$ λ > 420 nm (100 mW $cm^{-1}$) | 13.60 | - | 11.6% (450 nm) |
| Pt@CdS[28] (0.27 wt%) | Lactic acid (20 vol%) λ > 420 nm | 19.77 | 7.322 | - |
| MC-ZIS-2[29] | TEOA (10 vol%) λ ≥ 400 nm | 22.11 | - | 71.6% (420 nm) |
| SA-Pt/g-$C_3N_4$-87[30] (8.7 wt%) | TEOA (10 vol%) λ > 420 nm | 22.65 | 0.260 | 22.5% (420 nm) |
| $Rh/Cr_2O_3$/CoOOH/$SrTiO_3$:Al[31] | Pure water 300 W Xenon lamp | 35.4 | - | 95.7% (350 nm) |
| $RhCrO_x/SrTiO_3$: Al[32] | Pure water 300 W Xenon lamp (λ = 300-500 nm) | 37.5 | - | 56% ± 3% (365 nm) |

**Supplementary Table 9.** Band energies of $h$-ZIS, $Pt_{0.3}$-ZIS, and $Pt_{3.0}$-ZIS by UPS.

| Method | Material | Bandgap | $E_f$ | VBM | CBM |
|---|---|---|---|---|---|
| **UPS vacuum** | $h$-ZIS | 2.79 | -3.56 | -6.14 | -3.35 |
| | $Pt_{0.3}$-ZIS | 2.85 | -3.44 | -6.12 | -3.27 |
| | $Pt_{3.0}$-ZIS | - | -3.68 | -6.42 | - |
| **UPS V vs. NHE** | $h$-ZIS | 2.79 | -0.88 | 1.70 | -1.09 |
| | $Pt_{0.3}$-ZIS | 2.85 | -1.00 | 1.68 | -1.17 |
| | $Pt_{3.0}$-ZIS | - | -0.76 | 1.98 | - |

The relationship between the absolute electron potential of an electrode ($E_{\mathrm{abs}}$) and the standard electrode potential ($E^0$) is expressed as: $E_{\mathrm{abs}} = -E^0 - 4.44$, where the electron energy is 0 eV in a vacuum[33].

**Supplementary Table 10.** The simulated $R_s$ and $R_{ct}$ value from the equivalent circuit in Supplementary Fig. 40.

| Photocatalysts | $h$-ZIS | $Pt_{0.3}$-ZIS | $Pt_{3.0}$-ZIS |
|---|---|---|---|
| **$R_s$ (Ω)** | 544.8 | 1340 | 740.5 |
| **$R_{ct}$ (kΩ)** | 7563 | 403.8 | 1246 |

**Supplementary Table 11.** Adsorption free energy of H atom on different sites in $Pt_{SS}$-ZIS.

| Position | Adsorption free energy (eV) |
|---|---|
| Pt site | -0.36 |
| neighboring S site | -0.36 |
| neighboring Zn site | -0.36 |
| second-neighboring S site | 0.61 |
| second-neighboring Zn site | -0.36 |

# 4. Supplementary References


1. Ravel, B. & Newville, M. ATHENA, ARTEMIS, HEPHAESTUS: data analysis for X-ray absorption spectroscopy using IFEFFIT. *J. Synchrotron Radiat.* **12**, 537-541 (2005)
2. Kresse, G. & Furthmüller, J. Efficient iterative schemes for ab initio total-energy calculations using a plane-wave basis set. *Phys. Rev. B* **54**, 11169-11186 (1996).
3. Kresse, G. & Joubert, D. From ultrasoft pseudopotentials to the projector augmented-wave method. *Phys. Rev. B* **59**, 1758-1775 (1999).
4. Cheng N, *et al.* Platinum single-atom and cluster catalysis of the hydrogen evolution reaction. *Nat. Commun.* **7**, 13638 (2016).
5. Shi, X. *et al.* Inert basal plane activation of two-dimensional $ZnIn_2S_4$ *via* Ni atom doping for enhanced co-catalyst free photocatalytic hydrogen evolution. *J. Mater. Chem. A* **8**, 13376-13384 (2020).
6. Wang, P. *et al.* Atomic insights for optimum and excess doping in photocatalysis: a case study of few-Layer Cu-$ZnIn_2S_4$. *Adv. Funct. Mater.* **29**, 1807013 (2019).
7. Zhou, P. *et al.* Single-atom Pt-$I_3$ sites on all-inorganic $Cs_2SnI_6$ perovskite for efficient photocatalytic hydrogen production. *Nat. Commun.* **12**, 4412 (2021).
8. Liu, Y. *et al.* Ni single atoms anchored on nitrogen-doped graphene as $H_2$-Evolution cocatalyst of $SrTiO_3$/$CoO_x$ for photocatalytic overall water splitting. *Carbon* **183**, 763-773 (2021).
9. Xiao, M. *et al.* Molten-salt-mediated synthesis of an atomic nickel co-catalyst on $TiO_2$ for improved photocatalytic $H_2$ evolution. *Angew. Chem. Int. Ed.* **59**, 7230-7234 (2020).
10. Yang, W. *et al.* Enhanced photoexcited carrier separation in oxygen-doped $ZnIn_2S_4$ nanosheets for hydrogen evolution. *Angew. Chem. Int. Ed.* **55**, 6716-6720 (2016).
11. Xia, Y., Li, Q., Lv, K., Tang, D. & Li, M. Superiority of graphene over carbon analogs for enhanced photocatalytic $H_2$-production activity of $ZnIn_2S_4$. *Appl. Catal. B: Environ.* **206**, 344-352 (2017).
12. Wang, B., Deng, Z., Fu, X. & Li, Z. $MoS_2$/CQDs obtained by photoreduction for assembly of a ternary $MoS_2$/CQDs/$ZnIn_2S_4$ nanocomposite for efficient photocatalytic hydrogen evolution under visible light. *J. Mater. Chem. A* **6**, 19735-19742 (2018).
13. Lin, B. *et al.* Preparation of 2D/2D g-$C_3N_4$ nanosheet@$ZnIn_2S_4$ nanoleaf heterojunctions with well-designed high-speed charge transfer nanochannels towards high-efficiency photocatalytic hydrogen evolution. *Appl. Catal. B: Environ.* **220**, 542-552 (2018).
14. Zhou, P. *et al.* Strengthening reactive metal-support interaction to stabilize high-density Pt single atoms on electron-deficient g-$C_3N_4$ for boosting photocatalytic $H_2$ production. *Nano Energy* **56**, 127-137 (2019).
15. Chen, Y. *et al.* Hierarchical core–shell carbon nanofiber@$ZnIn_2S_4$ composites for enhanced hydrogen evolution performance. *ACS Appl. Mater. Interfaces* **6**, 13841-13849 (2014).
16. Wang, J. *et al.* Cubic quantum dot/hexagonal microsphere $ZnIn_2S_4$ heterophase junctions for exceptional visible-light-driven photocatalytic $H_2$ evolution. *J. Mater. Chem. A* **5**, 8451-8460 (2017).
17. Yuan, Y.-J. *et al.* $MoS_2$-graphene/$ZnIn_2S_4$ hierarchical microarchitectures with an electron transport bridge between light-harvesting semiconductor and cocatalyst: A highly efficient photocatalyst for solar hydrogen generation. *Appl. Catal. B: Environ.* **188**, 13-22 (2016).
18. Wang, S., Guan, B. Y., Wang, X. & Lou, X. W. D. Formation of hierarchical $Co_9S_8$@$ZnIn_2S_4$

heterostructured cages as an efficient photocatalyst for hydrogen evolution. *J. Am. Chem. Soc.* **140**, 15145-15148 (2018).

19. Li, X. *et al.* Single-atom Pt as co-catalyst for enhanced photocatalytic $H_2$ evolution. *Adv. Mater.* **28**, 2427-2431 (2016).
20. Zhang, S. *et al.* $MoS_2$ quantum dot growth induced by S vacancies in a $ZnIn_2S_4$ monolayer: atomic-level heterostructure for photocatalytic hydrogen production. *ACS Nano* **12**, 751-758 (2018).
21. Sui, Y. *et al.* Atomically dispersed Pt on specific $TiO_2$ facets for photocatalytic $H_2$ evolution. *J. Catal.* **353**, 250-255 (2017).
22. Zhang, L. *et al.* Ultra-low content of Pt modified CdS nanorods: one-pot synthesis and high photocatalytic activity for $H_2$ production under visible light. *J. Mater. Chem. A* **3**, 23732-23742 (2015).
23. Xiao, X. *et al.* A Promoted charge separation/transfer system from Cu single atoms and $C_3N_4$ layers for efficient photocatalysis. *Adv. Mater.* **32**, 2003082 (2020).
24. Wang, S., Wang, Y., Zhang, S. L., Zang, S.-Q. & Lou, X. W. Supporting ultrathin $ZnIn_2S_4$ nanosheets on Co/N-doped graphitic carbon nanocages for efficient photocatalytic $H_2$ generation. *Adv. Mater.* **31**, 1903404 (2019).
25. Zuo, Q. *et al.* Ultrathin metal–organic framework nanosheets with ultrahigh loading of single Pt atoms for efficient visible-light-driven photocatalytic $H_2$ evolution. *Angew. Chem. Inter. Ed.* **58**, 10198-10203 (2019).
26. Du, C. *et al.* Half-unit-cell $ZnIn_2S_4$ monolayer with sulfur vacancies for photocatalytic hydrogen evolution. *Appl. Catal. B: Environ.* **248**, 193-201 (2019).
27. Zhao X*, et al.* Perovskite microcrystals with intercalated monolayer $MoS_2$ nanosheets as advanced photocatalyst for solar-powered hydrogen generation. *Matter*, **3**, 935-949 (2020).
28. Wu, X. *et al.* Surface step decoration of isolated atom as electron pumping: Atomic-level insights into visible-light hydrogen evolution. *Nano Energy* **45**, 109-117 (2018).
29. Du, C., Yan, B. & Yang, G. Self-integrated effects of 2D $ZnIn_2S_4$ and amorphous $Mo_2C$ nanoparticles composite for promoting solar hydrogen generation. *Nano Energy* **76**, 105031 (2020).
30. Zeng, Z. *et al.* Single-atom platinum confined by the interlayer nanospace of carbon nitride for efficient photocatalytic hydrogen evolution. *Nano Energy* **69**, 104409 (2020).
31. Takata T*, et al.* Photocatalytic water splitting with a quantum efficiency of almost unity. *Nature* **581**, 411-414 (2020).
32. Goto Y*, et al.* A Particulate photocatalyst water-splitting panel for large-scale solar hydrogen generation. *Joule* **2**, 509-520 (2018).
33. Chun W-J*, et al.* Conduction and valence band positions of $Ta_2O_5$, TaON, and $Ta_3N_5$ by UPS and electrochemical methods. *J. Phys. Chem. B* **107**, 1798-1803 (2003).
