# Supplementary material for: Protruding Pt single-sites on hexagonal ZnIn2S4 to accelerate photocatalytic hydrogen evolution
Source: Nat Commun. 2022 Mar 11;13:1287. doi: 10.1038/s41467-022-28995-1 (PMC8917206; doi:10.1038/s41467-022-28995-1)
Supplement: Supplementary file 2 — Description of Additional Supplementary Files [file 41467_2022_28995_MOESM2_ESM.pdf]

Description of Additional Supplementary Files

File Name: Supplementary Movie 1
Description: $Pt_{0.3}$-ZIS thin film irradiated under visible light ($\lambda > 420$ nm).

File Name: Supplementary Movie 2
Description: $Pt_{0.3}$-ZIS thin film irradiated under simulated solar light.
